# Dietary Inulin Supplementation Modifies Significantly the Liver Transcriptomic Profile of Broiler Chickens

**DOI:** 10.1371/journal.pone.0098942

**Published:** 2014-06-10

**Authors:** Natalia Sevane, Federica Bialade, Susana Velasco, Almudena Rebolé, Maria Luisa Rodríguez, Luís T. Ortiz, Javier Cañón, Susana Dunner

**Affiliations:** Nutrigenómica Animal, Departamento de Producción Animal, Facultad de Veterinaria, Universidad Complutense de Madrid, Madrid, Spain; Wageningen UR Livestock Research, Netherlands

## Abstract

Inclusion of prebiotics in the diet is known to be advantageous, with positive influences both on health and growth. The current study investigated the differences in the hepatic transcriptome profiles between chickens supplemented with inulin (a storage carbohydrate found in many plants) and controls. Liver is a major metabolic organ and has been previously reported to be involved in the modification of the lipid metabolism in chickens fed with inulin. A nutrigenomic approach through the analysis of liver RNA hybridized to the Affymetrix GeneChip Chicken Genome Array identified 148 differentially expressed genes among both groups: 104 up-regulated (≥1.4-fold) and 44 down-regulated (≤0.6-fold). Quantitative real-time PCR analysis validated the microarray expression results for five out of seven genes tested. The functional annotation analyses revealed a number of genes, processes and pathways with putative involvement in chicken growth and performance, while reinforcing the immune status of animals, and fostering the production of long chain fatty acids in broilers supplemented with 5 g of inulin kg^−1^ diet. As far as we are aware, this is the first report of a microarray based gene expression study on the effect of dietary inulin supplementation, supporting further research on the use of this prebiotic on chicken diets as a useful alternative to antibiotics for improving performance and general immunity in poultry farming, along with a healthier meat lipid profile.

## Introduction

Prebiotics (e.g. fructans including inulin-type fructans [inulin and fructooligosaccharides]) are nondigestible food ingredients, whose beneficial effects on the host result from the selective stimulation of growth and/or activity of members of the gut microbiota, specifically bifidobacteria and lactobacteria [1]. Inulin, generally extracted from chicory roots (*Cichorium intybus* L.), is a prebiotic formed by a chain of fructose molecules connected by β-(2–1) glycosidic bonds, terminated by one glucose molecule, which is not decomposed by digestive enzymes due to its chemical structure [2]. However, it is a perfect carbon source for health-promoting gut bacteria. Although the inclusion of prebiotics in the diet is known to be advantageous, their use in farm animals has been scarce [3]. Fructans supplementation is known to produce positive influences both on health and growth [4], [5]: in fish, they increase intestinal growth relative to whole body weight, potentially enhancing nutrient absorption [6], [7]; in broilers, a decrease in body fat deposition [8], serum cholesterol concentration and abdominal fat weight has been reported [4], [5], [7], [9]; in rodents and, to a lesser extent in humans, inulin-type fructans can alter lipid metabolism by reducing plasma triglyceride and cholesterol concentrations [10], [11]; in several animal models and in birds, these prebiotics also modify the hepatic metabolism of lipids [5]; finally, prebiotics have also other positive effects on health, improving body functions and bone health, decreasing disease risks, reinforcing immune functions, preventing infections and intestinal diseases, and enhancing bioavailability of minerals (calcium and magnesium) [7], [12], [13]. However, the mechanisms through which these effects develop are not clear: it is thought to be a direct effect of the prebiotic on the host immune system by triggering receptors in the gut epithelium, which induces an immune response and activates the immune system without it becoming overactive [14]; withal, many of the desired effects are brought about by the manipulation of the gut flora, with the prebiotics providing substrates that preferentially encourage beneficial strains of bacteria to proliferate [1].

In this study, we perform a nutrigenomic approach to understand the molecular mechanisms underlying inulin supplementation effects to assess its impact in the commercial broiler. We chose to study the liver transcriptome as it is a major metabolic organ involved in many physiological processes including energy metabolism, detoxification and innate immunity. Moreover, previous results obtained in chickens by Rebolé et al. [4] and Velasco et al. [5] pointed to the modification of the hepatic metabolism of lipids by inulin. The different expression patterns from a nutrigenomic point of view help understand the mechanisms by which inulin modulates both metabolism and general immunity. Results outlined below indicate major changes in transcription of a number of genes implicated in development and maintenance of different tissues, particularly muscle and nervous system, fatty acid and protein metabolism, and immune system, gene transcription, and cell development and maintenance processes in the liver.

## Material and Methods

A flow diagram of study design and results is shown in Fig. S1.

### Animals

The animal protocol was approved by the Animal Care and Ethics Committee of the Universidad Complutense de Madrid (Spain) (CEA-UCM/32). Birds were handled according to the principles for the care of animals in experimentation established by the Spanish Royal Decree 1201/2005 [15].

A total of 80 one-day-old female broiler chicks (Cobb 500 genetic line) obtained from a commercial hatchery (Cobb Espanola S.A., Alcalá de Henares, Spain) were randomly allocated into 16 pens with eight replicates per treatment and five chicks per pen as described by Velasco *et al*. [5]. The bird groups were assigned to two dietary treatments: 1) control diet without inulin; and 2) control diet plus 5 g of inulin kg^−1^ of diet, which gave the best results on decreasing blood concentrations of triacylglycerides and increasing the capacity of sunflower oil to enhance the ratio of polyunsaturated (PUFA) to saturated (SFA) fatty acids of intramuscular fat in broilers [5]. The control basal diet (Table 1) was formulated to be adequate in all nutrients [16] and was prepared in mash form. The inulin source used in the current study was a commercial product (Prebiofeed, Qualivet, Las Rozas, Spain) obtained from chicory (*C. intybus L.*) roots containing 746 g kg^−1^ inulin-type fructans as determined in our laboratory [4]; therefore, the amount of this product added to the corresponding control diet at the expense of the entire diet was 6.7 g of product kg^−1^ of diet to obtain 5 g of inulin kg^−1^ of diet. Diets in mash form and water were offered *ad libitum* through the 34 day feeding trial. Mortality was lower than 3%. At the end of the experiment, birds were weighed and killed by cervical dislocation and liver tissue (∼1 g) was placed in RNAlater (Ambion) and stored at 4°C for 24 h followed by long term storage at −20 °C prior to RNA extraction.

**Table 1:** Ingredients and nutrient composition of experimental control diet (g kg^−1^ as fed basis).

Ingredient	
Corn	451.8
Soybean meal (44% CP)	418.7
Sunflower oil	90.0
Calcium carbonate	10.0
Dicalcium phosphate	18.5
Sodium chloride	3.0
DL-Methionine	1.5
Antioxidant (butylated hydroxytoluene)	1.5
Vitamin and mineral premix^1^	5.0
Nutrient composition	
CP^2^	217.0
Lysine^2^	12.8
Methionine^2^	5.2
Methionine plus Cystine^3^	9.2
AME_n_ 3 (kcal kg^−1^)	3,152
Fatty acids^2^^,^ 4 (g kg^−1^ of total fatty acids)	
C16:0	85.2
C18:0	36.6
C18:1n-9	299.1
C18:2n-6	548.0
SFA	121.8
MUFA	308.8
PUFA	556.6
UFA	865.4
PUFA:SFA	4.6
UFA:SFA	7.1

1 Premix supplying (mg kg^−1^ diet): 3 retinol, 55 cholecalciferol, 25 *dl-α-*tocopheryl acetate, 2.5 menadione, 3 thiamine, 6 riboflavin, 7 pyridoxine, 0.2 folic acid, 0.02 cyanocobalamin, 0.2 biotin, 25 calcium pantothenate, 50 niacin, 1300 choline chloride, 60 Mn, 80 Fe, 50 Zn, 5 Cu, 0.1 Se, 0.18 I, 0.5 Co, 0.5 Mo.

2 Determined.

3 Calculated.

4 SFA  =  saturated fatty acids; MUFA  =  monounsaturated fatty acids; PUFA  =  polyunsaturated fatty acids; UFA  =  unsaturated fatty acid.

### RNA extraction, cDNA synthesis and microarray analysis

Total RNA was extracted from 25 mg of liver tissue using the RNeasy Tissue Mini Kit (QIAGEN, Izasa, Spain). Four pools were produced consisting each in four equivalent amounts of liver samples mixed together according to the supplemented and control groups (Fig S1). Each experimental group resulted in eight samples combined in two pools which were RNA extracted for hybridization in microarray. Changes in gene expression were analyzed by microarray technology using the Affymetrix GeneChip Chicken Genome Array. Briefly, 200 ng of total RNA from each sample were processed, labeled, fragmented, and hybridized to the GeneChipChicken Genome Array according to the manufacturer recommendations.

The microarray normalization was carried out using functions from the Babelomics [17]. Normalized data were further analyzed using R (version 3.0.2) and the Bioconductor Limma package [18]. Differential gene expression was measured by empirical Bayes t-statistics and *P-values* were adjusted for false discovery rate correction [19]. Only the genes with *P-value* ≤0.09 and log fold change greater or equal than 1.4-fold for up-regulated genes and lower or equal than 0.6-fold for down-regulated genes were screened out as differentially expressed genes.

### Gene ontology analysis and visual pathway analysis

The Database for Annotation, Visualization and Integrated Discovery (DAVID) v6.7b [20] was used to determine pathways and processes of major biological significance and importance through the Functional Annotation Cluster (FAC) tool based on the Gene Ontology (GO) annotation function. DAVID FAC analysis was conducted on two independent gene lists containing up-regulated genes (≥1.4-fold) and down-regulated genes (≤0.6-fold) at *P*≤0.09. High stringency ease score parameters were selected to indicate confident enrichment scores of functional significance and importance of the given pathways and processes investigated.

Kyoto Encyclopedia of Genes and Genomes (KEGG) pathway tool was used to visually map clusters of the same chicken genes involved in common pathways and processes for both pathway-specific and molecular overview purposes. KEGG pathway tools were utilized through DAVID online tools.

### Real-time PCR validation

To confirm microarray data, regulated genes in the liver tissue (*ITIH5*, *DIO2*, *GIMAP5*, *USP18*, *KIAA1754*, *CCDC79*) were selected for further validation by qRT-PCR. A total of 16 samples, eight corresponding to the inulin supplemented, and eight corresponding to the non supplemented chickens (all included in the four pools used to hybridize the microarray) were used. The total RNA was used for RT-cDNA synthesis using Superscript II First Strand cDNA Synthesis kit (Invitrogen). The resulting cDNA template was used to conduct real-time assays for all six target genes and four commonly reference genes, beta-actin (*ACTB*), glyceraldehyde-3-phosphate dehydrogenase (*GAPDH*), hypoxanthine phophoribosyl-transferase (*HPRT*) and glucose-6-phosphate dehydrogenase (*G6PDH*), by using an iCycler IQ Real-Time PCR thermocycler (Bio-Rad) and Dynamo HS SYBR Green qPCR Kit (Finnzymes, Vitro, Spain) as master mix. Primers were designed based on public available sequences (Table S1) using *primer 3* (http://bioinfo.ut.ee/primer3-0.4.0/primer3/). After the selection of the most adequate annealing temperature, standard curves and the sample assays were produced in triplicate for each gene, together with the no-template controls. The following experimental run protocol was used: quantification program consisting of 42 cycles of 95°C for 30 s, 30 s at annealing temperature and 40 s at 72°C, ending with a melting program of 155 cycles of 10 s at 55°C and continuous fluorescence measurement. The results were exported into Microsoft Excel's based software Gene Expression Macro Version 1.1 (Bio-Rad Laboratories, http://www.bio-rad.com/) to calculate and normalize the expression of each gene.

The expression stability and level of the reference genes were measured using three different statistical algorithms to rank the genes by their stability values, geNorm [21], NormFinder [22] and Bestkeeper [23], being this a necessary process to guarantee that the reference genes are constitutively expressed in the tissue and treatment in question for a correct normalization. The target gene data were analyzed using PROC GLM procedure of the SAS statistical package v. 9.1.3 [24] to estimate the 2Δ^Ct^ differences between treatments. Relative Expression Software (REST), which follows the Pfaffl method [25] was also used. This mathematical algorithm computes an expression ratio based on qRT-PCR efficiency and the crossing point deviation of the sample compared to a control group: R  =  [(E target gene)^ΔCt target gene (control-sample)^]/[(E Ref gene)^ΔCt Ref gene (control-sample)^], where E is PCR efficiency of the gene transcript determined by standard curve using a serial dilution of cDNA. Normalization of the expression levels of the target genes was performed through three reference genes (*GAPDH*, *G6PDH* and *ACTB*). When differences between samples of either experiment (with or without inulin) were subjected to random, a test was carried out and the alternative hypothesis accepted for a *P* value lower than 0.08.

## Results

### Transcriptome profile and differential expression

A total of four Affymetrix GeneChip Chicken Genome arrays were hybridized with RNA pools resulting from mixing equal amounts of four different RNA samples from each experiment. Comparative transcriptome profiling of liver RNA samples from experimental inulin fed group versus control cDNA pools identified 112 genes over-expressed according to the elected threshold ≥1.4-fold and 46 down-regulated ≤0.6-fold (*P*≤0.09) (Table S2) from a total of 38,450 probes corresponding to over 28,000 chicken genes. The widespread use of arbitrary fold change cut-offs of above 2 and significance *P-values* of <0.02 in the analysis of microarray results was discarded here as it leads data collection to look only at genes which vary wildly amongst other genes, and raises questions as to whether the biology or the statistical cutoff are more important within the interpretation [26]. In this paper we analyzed data giving priority to the biological signification of the results and set the fold change threshold at ≥1.4-fold for over-expressed genes and ≤0.6-fold for down-regulated genes. Among these 158 sequences, 139 shared significant homology with genes encoding proteins of known function, 9 shared homology with genes encoding proteins of unknown function, and 10 shared no significant homology with any database accession (Table S2). Out of the 148 chicken sequences with homologs, 104 were up-regulated and 44 were down-regulated in the presence of dietary inulin.

### Functional annotation analyses

**Figure 1:**
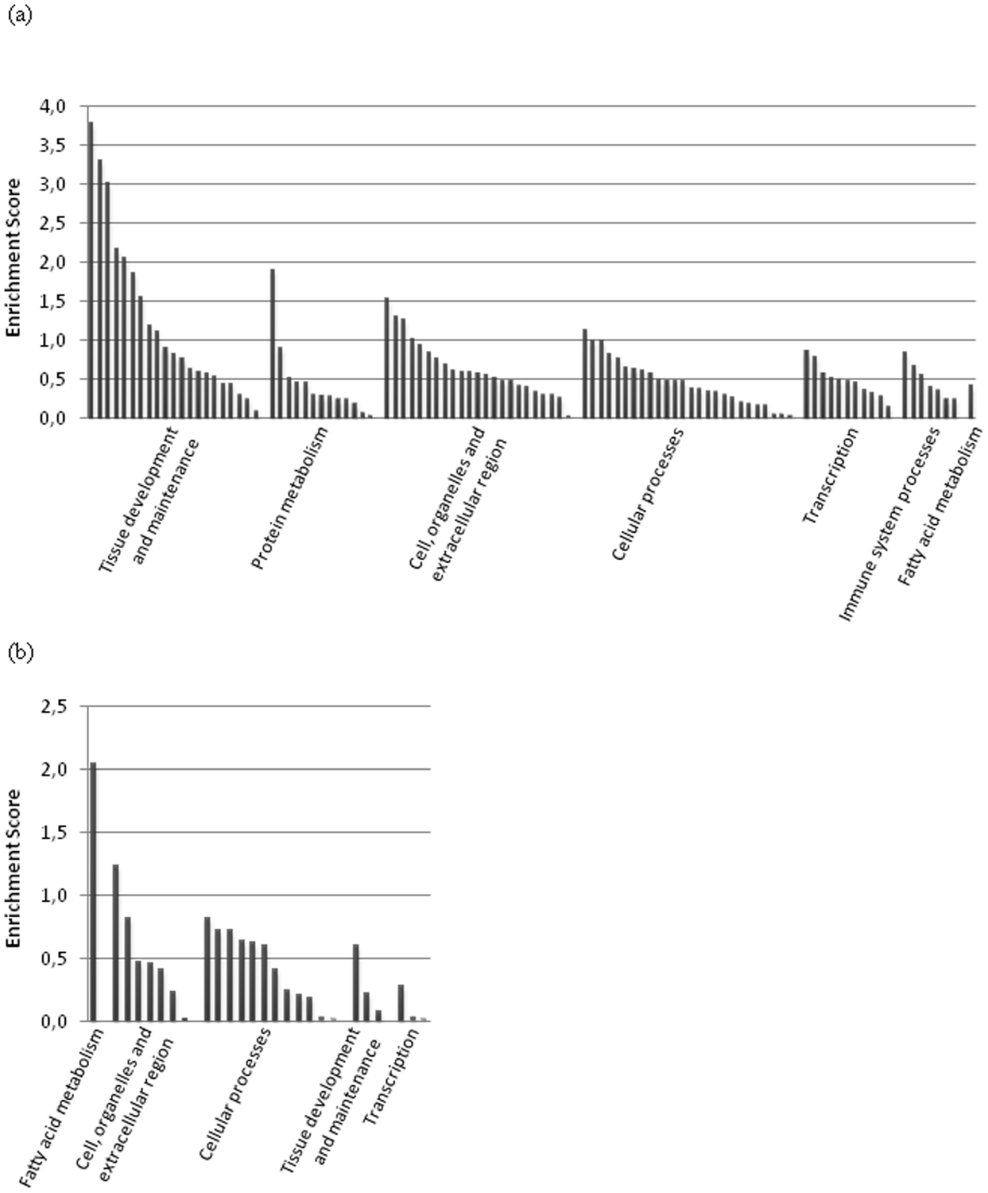
DAVID Functional Annotation Cluster (FAC) analysis of differentially expressed genes between chickens with and without 5 g of inulin kg^-1^ diet supplementation. DAVID FAC analysis was conducted on two independent gene lists containing 95 up-regulated genes (≥1.4-fold) and 35 down-regulated genes (≤0.6-fold) and P≤0.09. High stringency ease score parameters were selected, to indicate confident enrichment scores of functional significance and importance of the given pathways and processes investigated. (A) Grouped major FACs for up-regulated genes (≥1.4-fold). (B) Grouped major FACs for down-regulated genes (≤0.6-fold). Significance is determined by corresponding enrichment scores.

The expression data was analyzed using the DAVID FAC tool, obtaining enrichment scores per cluster under high stringency conditions as an indication of the biological significance of the gene groups analyzed (Table S3). From the 148 sequences with homologs, DAVID FAC analysis included 95 up-regulated and 35 down-regulated sequences in the analysis, revealing 102 enriched functional clusters with strong confident enrichment scores in the up-regulated sequences for development and maintenance of different tissues, particularly muscle and nervous system, cell processes, protein metabolism, gene transcription, response to hormones, and immune system processes, whereas the down-regulated sequences -although showing only 26 enriched functional clusters with lower enrichment scores- highlighted mainly fatty acid metabolism and intracellular organelles (Table S3, Fig. 1).

The KEGG database retrieved five pathways (Table 2, Fig. S2): Adipocytokine Signaling Pathway, Glycosphingolipid Biosynthesis, Glutathione Metabolism, Drug Metabolism - Cytochrome P450, and Metabolism of Xenobiotics by Cytochrome P450. The list of differentially expressed genes encoding proteins of known function but not included in the DAVID FAC analysis (9), along with those genes encoding proteins of unknown function (9) is shown in Table S4.

**Table 2:** List of chicken genes from KEGG pathway maps differentially expressed in liver from animals supplemented with 5^−1^ diet and controls, with expression ratio, annotated gene description and KEGG ID.

Gene Symbol	Expression Ratio With vs. Without	Gene Name	KEGG ID
Adipocytokine Signaling Pathway - KEGG pathway			
*TNFRSF1B*	1.9	Tumor necrosis factor receptor superfamily member 1B	TNFR1
*ACSL6*	1.4	Acyl-CoA synthetase long-chain family member 6, transcript variant X5	FACS
*PPARA*	1.7	Peroxisome proliferator-activated receptor alpha	PPARα
			
Glycosphingolipid Biosynthesis - Ganglio Series - KEGG pathway			
*ST3GAL5*	1.6	ST3 beta-galactoside alpha-2,3-sialyltransferase 5	2.4.99.9
*ST3GAL1*	1.9	ST3 beta-galactoside alpha-2,3-sialyltransferase 1	2.4.99.4
			
Glutathione Metabolism - KEGG pathway			
*GSTA*	0.6	Glutathione S-transferase class-alpha	2.5.1.18
*GSTT1*	0.6	Glutathione S-transferase theta 1	2.5.1.18
*RRM2B*	1.5	Ribonucleotide reductase M2 B (TP53 inducible)	1.17.4.1
			
Drug Metabolism - Cytochrome P450 - KEGG pathway			
*GSTA*	0.6	Glutathione S-transferase class-alpha	2.5.1.18
*GSTT1*	0.6	Glutathione S-transferase theta 1	2.5.1.18
			
Metabolism of Xenobiotics by Cytochrome P450 - KEGG pathway			
*GSTA*	0.6	Glutathione S-transferase class-alpha	2.5.1.18
*GSTT1*	0.6	Glutathione S-transferase theta 1	2.5.1.18

### Validation of microarray data by real-time RT-PCR

In order to validate the microarray results, qRT-PCR was performed to determine the expression levels of six chicken genes -*ITIH5, DIO2, USP18, CCDC79, KIAA1754, GIMAP5-* selected from the list of sequences differentially expressed across individuals from inulin and control groups. Also, to obtain reliable qRT-PCR results, the stability of four commonly used housekeeping genes was determined (*HPRT, ACTB*, *GAPDH,* and *G6PDH*). The results of three programs (GeNorm, BestKeeper, and NormFinder) revealed that *ACTB*, *GAPDH,* and *G6PDH* were good candidate reference genes (Table 3). Despite a non significant 1.3 fold differential expression in the microarray results for *HPRT*, this gene was significantly differentially expressed when analyzed through qRT-PCR. Thereof, *HPRT* was not suitable as an endogenous control for the analysis of gene expression in the liver tissue in chickens.

**Table 3:** Stability of four reference genes on liver from animals supplemented with 5^-1^ diet and controls, measured through three different software: Bestkeeper, GeNorm and NormFinder.

	Bestkeeper		GeNorm		NormFinder	
	Stability value	Ranking	Stability value	Ranking	Stability value	Ranking
***ACTB***	0.959	1	0.659	1	0.247	3
***GAPDH***	0.934	2	0.690	3	0.304	2
***G6PDH***	0.921	3	0.727	1	0.372	1
***HPRT***	0.835	4	0.805	4	0.466	4

The qRT-PCR expression results using REST software (Table 4) correlated with the microarray expression data for 4 out of the 6 genes tested, plus the up-regulation of *HPRT*. The qRT-PCR determination of *ITIH5*, *DIO2*, *KIAA1754*, *GIMAP5,* and *HPRT* mRNA levels showed a 2.2, 6.2, 2.4, 2.5, and 1.9-fold increase respectively in inulin supplemented chickens over controls, these results comparing favorably to the 2.2, 4.3, 3.3, 3.2, and 1.3–fold increase in expression determined by the microarray analysis.

**Table 4:** Differential expression results of the genes studied in the liver by Real-time PCR assay from animals supplemented with 5 g of inulin kg^−1^ diet and controls using REST software (*P<0.08*).

Gene	Type	Expression	Std. Error	95% C.I.	P(H1)	Result
*ACTB*	REF	0.828				
*GAPDH*	REF	1.093				
*G6PDH*	REF	1.106				
*ITIH5*	TRG	2.185	0.88–5.3	0.40–7.4	0.080	UP
*DIO2*	TRG	6.175	2.29–17.6	1.00–33.6	0.003	UP
*USP18*	TRG	1.082	0.44–3.0	0.16–6.4	0.880	
*CCDC79*	TRG	1.484	0.35–4.3	0.12–16.6	0.538	
*KIAA1754*	TRG	2.376	0.89–6.0	0.44–11.3	0.070	UP
*GIMAP5*	TRG	2.543	1.04–6.0	0.62–14.2	0.042	UP
*HPRT*	TRG	1.860	1.33–2.8	0.63–3.7	0.015	UP

Among the significant differentially expressed genes, *ITIH5*, *DIO2*, *GIMAP5*, and *HPRT* showed significant 2Δ^Ct^ differences between treatments when analyzed using PROC GLM procedure (SAS) (Table 5). The activity of *ITIH5*, *DIO2*, *GIMAP5*, and *HPRT* genes explained 30%, 39%, 26% and 49% of the total variability (*P*<0.08), respectively.

**Table 5:** ANOVA results of the genes studied in the liver by Real-time PCR assay from animals supplemented with 5 g of inulin kg^−1^ diet and controls.

Dependent Variable	Mean Square	Error	R-Square	Coeff Var	Pr > F
*ITIH5*	48.4	11.2	0.302	71.3	0.0642
*DIO2*	607.5	94.7	0.390	88.5	0.0297
*USP18*	55.9	82.5	0.063	113.4	0.4296
*CCDC79*	179.5	216.0	0.077	123.4	0.3835
*KIAA1754*	37.2	19.1	0.178	87.8	0.1962
*GIMAP5*	78.6	22.7	0.257	80.1	0.0922
*HPRT*	6.2	0.63	0.495	34.1	0.0106

However, the expression analysis of *USP18* and *CCDC79* by qRT-PCR was not significant, in contrast with the microarray data which showed both genes to be down-regulated by 0.5-fold.

## Discussion

The use of inulin-type fructans in poultry feeding is known to produce positive influences both on chicken health and growth, by improving the performance [3], [4], increasing the absorption of nutrients by modifications on the intestinal mucosal structure [27], [28], [29], stimulating the growth and/or activity of beneficial intestinal bacteria and preventing colonization by pathogenic bacteria [1]. Also, they decrease body fat deposition and improve its profile [4], [5], [7], [8], [9], along with other positive effects on health [7], [12], [13]. This study adds the characterization of the genetic expression patterns promoted by inulin to the evaluation on the effects of its dietary supplementation in poultry. Moreover, understanding the molecular mechanism underlying inulin effects can be a useful approach to help finding natural alternatives to the overdependence on antibiotics to enhance animal production (given that it has been directly related to the growing number of antibiotic resistances [30], which has lead to the ban of antibiotics for growth promotion by the European Union since 2006 [31] and the calls to restrict its use in other countries).

Functional analysis of the differentially expressed genes using the GO term annotations showed that the differentially expressed genes can be functionally grouped in three main classes: (i) basal processes including tissue development and maintenance (particularly muscle, nervous system processes, cell organelles processes, protein metabolism, gene transcription, and response to hormones); (ii) immune system processes; and (iii) fatty acid metabolism.

### Basal processes – development and maintenance of different tissues

Although liver was the tissue explored, genes involved in the development and maintenance of different tissues, particularly nervous system and muscle, showed the highest enrichment score in the FAC analysis of up-regulated genes (Fig. 1(a)). DAVID analyses identified 46 genes with an expression range of 1.4 to 4.5 that functionally clustered into common GO terms related to nervous system, muscle, respiratory, bone, and embryonic development, or neurological, circulatory and reproductive processes (Table S3). Thus, several differentially expressed genes may relate to other tissue-specific processes that, up to now, have not been described as expressed in liver. As an example, the high enrichment scores of neurological pathways may be due to the involvement of a neurological mechanism, e.g. through the participation of neuronal tissue in liver tissue composition. Alternatively, it could indicate that genes currently described as involved in neurological pathways, may have basic functions common to other tissues.

FAC analysis also identified protein metabolism as a significant biological process up-regulated by the addition of inulin to the chicken diets (Fig. 1(a)). DAVID analyses identified 33 genes with an expression range of 1.4 to 4.3 that functionally clustered into common GO terms related to regulation of protein metabolism, translation, peptidase activity, post-translational protein modification, protein activity, proteolysis, or protein localization (Table S3). Among them, *CAV2*, *TPPP*, *MLH1*, *AHCTF1*, *NRG1*, *NEFL*, and *DVL1* were also involved in the improvement of growth performance by inulin supplementation, showing the highest enrichment scores.

DAVID analyses identified 43 genes with an expression range of 1.4 to 4.3 that functionally clustered into common GO terms related to regulation of transcription and biosynthesis, chromosome organization, DNA, RNA and nucleotide binding, or transcription activity (Fig. 1(a), Table S3), showing an increased cellular activity in the presence of the prebiotic.

Finally, regarding cell organelles and cellular processes, DAVID analyses included all up-regulated genes (expression range of 1.4 to 4.5) into common GO terms related to these main groups (Fig. 1, Table S3).

All these data suggested an important influence of inulin on processes and pathways that lead to the increase of growth and performance, which is in agreement with the results reported by Rebolé *et al.* [4] and Velasco *et al.* [5], who found a quadratic body weight gain in chickens supplemented with inulin, and with those of Tacchi *et al.* [6], who suggested an improvement in nutrient absorption as a consequence of the increase of intestinal growth caused by the addition of inulin to fish diets.

### Immune system processes

The addition of inulin to the diet of chickens stimulated various immune system processes (Fig. 1(a)). DAVID analyses identified 20 up-regulated genes with an expression range of 1.4 to 4.5 that functionally clustered into common GO terms related to immune system processes, immunoglobulins, response to virus and biotic stimulus, regulation of apoptosis, immune system development and activation, immune response, or cellular response to stress and DNA damage stimulus (Table S3).

KEGG pathway visual analysis identified three genes (*TNFRSF1B, ACSL6, PPARA*) in the Adipocytokine Signaling Pathway that were up-regulated with inulin supplementation by 1.56 to 7.56 fold (Fig. S2(a), Table 2). However, *ACSL6* and *PPARA* were not included in the functional clusters related to immune system processes by DAVID FAC tool (Table S3). *TNFRSF1B* has anti-apoptotic activity by stimulating antioxidative pathways and is considered a marker of activation of T-helper subsets regulatory T-cell (Tregs) [32]. *ACSL6* encodes an enzyme that catalyzes the formation of acyl-CoA from fatty acids, ATP, and CoA, using magnesium as a cofactor, and as such was included in the mitochondrion, membrane, fatty acid metabolism, metal ion binding and carboxylic acid metabolism DAVID clusters (Table S3). Finally, *PPARA*, found in different clusters, is a member of peroxisome proliferator-activated receptors (PPARs), which plays a major regulatory function of genes involved in energy metabolism, affects the expression of target genes involved in cell proliferation and differentiation, and in immune and inflammation responses (see e.g. Gervois & Mansouri [33]).

Interestingly, Glutathione Metabolism is found among the pathways identified by KEGG (Fig. S2(c), Table 2). Glutathione plays important roles in antioxidant defense, nutrient metabolism, and regulation of cellular events (including gene expression, DNA and protein synthesis, cell proliferation and apoptosis, signal transduction, cytokine production and immune response, and protein glutathionylation); its deficiency contributes to oxidative stress, which plays a key role in the aging and the pathogenesis of many diseases [34]. Three genes where included in this pathway: *GSTT1* is a member of a superfamily of proteins that catalyze the conjugation of reduced glutathione to a variety of electrophilic and hydrophobic compounds identified as having an important role in human carcinogenesis; together with *GSTA* form the Glutathione S-transferase cluster, and both genes were down-regulated in contrast to the up-regulated *RRM2B*, which is found to act in *Trypanosoma cruci* as a mechanism to minimize the reactive oxygen species (ROS) produced by host defense [35].

These results pointed towards an effect of inulin supplementation on the reinforcement of chicken immune status by the activation of genes and pathways implicated in immune processes, while conferring a higher ability to avoid ROS.

### Fatty acid metabolism

Fatty acid metabolism showed the highest enrichment score in the FAC analysis of down-regulated genes (Fig. 1(b)). DAVID analyses identified 5 genes with an expression range of 0.5 to 0.6 that functionally clustered into common GO terms related to lipid, monocarboxylic acids, carboxylic acids, oxoacids, organic acids, and ketone metabolic processes (Table S3).

Inulin supplementation seemed to foster the production of beneficial long chain fatty acid [36], as deduced from the higher expression of genes as *PPARA*, *FASN*, and *ACSL6*, and the down regulation of genes involved in the degradation of long branched fatty acids (*ACOX2*), hydrolysis of fatty acids, specifically phospholipids (*JMJD7-PLA2G4B*), cleavage of the ether bond of alkylglycerols (*TMEM195*), and in processes specifically linked with the mitochondrion and cytoplasma (*CYP2J2*). The down-regulation of a fatty acid elongase (*ELOVL2*) can be explained by its particular substrate: whereas *ELOVL2* can efficiently elongate C_20_ and C_22_ polyunsaturated (PUFA) fatty acids, it cannot elongate C_18_ PUFA nor monounsaturated fatty acids (MUFA) or saturated fatty acids (SFA) [37], and its down-regulation is in concordance with the observed effect of dietary inulin addition on the increase of C18:2n-6 [5].

As supported by Rebolé *et al.* [4] and Velasco *et al.* [5], the addition of inulin-type fructans to the diet decreases body fat deposition [8], serum cholesterol concentration, and abdominal fat weight of chickens [9], which is in agreement with the gene regulation found here and explained also the increased function of the mitochondrion linked to the different fatty acid metabolism.

### Validation of microarray data by real-time RT-PCR

In order to validate the microarray results, six chicken genes, selected from the list of genes differentially expressed across controls and inulin supplemented individuals, were used in qRT-PCR. To avoid bias, the tested genes were chosen from an array of different processes including biological regulation (Deiodinase iodothyronine type II, *DIO2*), biologic and metabolic process (Inter-alpha globulin inhibitor H5, *ITIH5*), immune system processes (IMAP family member 5, *GIMAP5*), protein metabolism (Ubiquitin Specific Protease 18, *USP18*), gene transcription and regulation processes (Coiled-coil domain containing protein 79 CCD79, *CCDC79*), and cell development and maintenance processes (Inositol 1,4,5-trisphosphate receptor interacting protein, similar to *KIAA1754*-like) FAC groups. *HPRT*, initially chosen as reference gene, failed as housekeeping gene for the analysis of gene expression in liver tissue in broiler chickens due to its significant differential expression when analyzed with qRT-PCR, highlighting the importance of the correct selection of reference genes [38].

Among the genes showing differential expression, it is worth highlighting *ITIH5*, which explained 30% of the total variability found between the two feeding groups, *DIO2* -39%-, *GIMAP5* −26%-, and *HPRT* −49%-. *ITIH5* encodes a secreted protein and is known to be highly expressed in subcutaneous adipose tissue, and associated with measures of body size and metabolism [39]. The protein encoded by *DIO2* belongs to the iodothyronine deiodinase family and activates thyroid hormone, which acts on nearly every cell in the organism -increases the basal metabolic rate, affects protein synthesis, helps regulate long bone growth and neural maturation-, being essential to proper development and differentiation of all cells types and the regulation of protein, fat, and carbohydrate metabolism [40]. KIAA1754 is an intracellular channel protein that mediates calcium (Ca^2+^) release from the endoplasmic reticulum, and is involved in many biological processes (e.g. fertilization, muscle contraction, secretion, cell growth, differentiation, apoptosis, and synaptic plasticity) [41]. The protein encoded by *HPRT* gene plays a central role in the generation of purine nucleotides through the purine salvage pathway [42]. Thereof, the up-regulation of *ITIH5, DIO2, KIAA1754* and *HPRT* by inulin supplementation is in agreement with the higher basal activity required by an increased body weight gain reported in chickens by Rebolé *et al.* [3] and Velasco *et al.* [4].

*GIMAP5* encodes a protein belonging to the GTP-binding superfamily and to the immune-associated nucleotide (IAN) subfamily of nucleotide-binding proteins, and has been implicated in autoimmune diseases, lymphocyte homeostasis and apoptosis [43].

Down-regulation of *USP18* gene, which belongs to the ubiquitin-specific proteases (UBP) family of enzymes, and *CCDC79,* one of the principal subunit oligomerization motifs in proteins, in inulin supplemented chickens was not validated by qRT-PCR. While qRT-PCR results are usually accurate and gene specific, it is possible that the microarray hybridization results can be biased for genes encoded by multigene families.

## Conclusion

As far as we have notice, this is the first report of a microarray based gene expression study on the effect of inulin supplementation in any animal species. The results obtained here highlighted the functional significance and importance of inulin supplementation on processes and pathways that lead to an increase in growth and performance, while reinforcing the immune status of chickens, and fostering the production of long chain fatty acids in broilers supplemented with 5 g of inulin kg^−1^ diet. Additional information on the molecular mechanism underlying the inulin effects on gene activity and the cellular basis of feed efficiency in broilers is also provided. This nutrigenomic study supports further research on the supplementation of chicken diets with the prebiotic inulin at 5g kg^−1^ diet as a possible and useful alternative to the use of antibiotics for improving animal production and general immunity in poultry farming, along with a healthier meat lipid profile.

## Supporting Information

Table S1 **Reference and selected target genes used in the Real-time PCR assay indicating name, GenBank accession number or reference (Accession), and primers used for the expression study.**
(PDF)

Table S2 **One hundred and twelve up-regulated genes (≥ 1.4-fold) and 46 down-regulated genes (≤ 0.6-fold) showing a** ***P-value*** **≤ 0.09 when using an Affymetrix GeneChip Chicken Genome Array in RNA from chicken broilers supplemented with inulin.**
(PDF)

Table S3 **Gene list report and complete results for the 95 up-regulated and 35 down-regulated genes for which annotation information was available at DAVID Bioinformatics Resources 6.7 (** http://david.abcc.ncifcrf.gov/ **) in August 2013, including the corresponding DAVID scores (** ***P values*** **) and the lists of genes in every significant category.** A summary of the Functional Annotation Clusters (FAC) is also shown from page 108 to 112.
(PDF)

Table S4 **List of differentially expressed genes encoding proteins of known function but not included in the DAVID FAC analysis, and genes encoding proteins of unknown function.**
(PDF)

Figure S1 **Flow diagram of study design and results.**
(PDF)

Figure S2 **KEGG pathway maps of chicken differentially expressed genes involved in common pathways and processes.** KEGG pathway tools were utilized through DAVID online tools and the analysis were conducted on two independent gene lists containing 95 up-regulated genes (≥ 1.4-fold) and 35 down-regulated genes (≤ 0.6-fold) and P ≤ 0.09. (a) Adipocytokine Signaling Pathway; (b) Glycosphingolipid Biosynthesis - Ganglio Series; (c) Glutathione Metabolism; (d) Drug Metabolism - Cytochrome P450; (e) Metabolism of Xenobiotics by Cytochrome P450. Red boxes indicate up-regulated and down-regulated homologs. All chicken homologs identified on the KEGG maps are shown in Table 2.
(PDF)
