# Supplementary material for: Dietary Inulin Supplementation Modifies Significantly the Liver Transcriptomic Profile of Broiler Chickens
Source: PLoS One. 2014 Jun 10;9(6):e98942. doi: 10.1371/journal.pone.0098942 (PMC4051581; doi:10.1371/journal.pone.0098942)
Supplement: Table S1 — Reference and selected target genes used in the Real-time PCR assay indicating name, GenBank accession number or reference (Accession), and primers used for the expression study. (PDF) [file pone.0098942.s001.pdf]

Table S1. Reference and selected target genes used in the Real-time PCR assay indicating name, GenBank accession number or reference (Accession), and primers used for the expression study.

Locus Symbol	GenBank Accession N°	Forward multiplex primers (5' → 3')	Reverse multiplex primers (5' → 3')
ACTB	L08165	CACAGATCATGTTTGAGACCTT	CATCACAATACCAGTGGTACG
GAPDH	K01458	GGGAAGCTTACTGGAATGGCT	GGCAGGTCAGGTCAACAACA
G6PDH	AI981686	CGGGAACCAAATGCACTTCGT	GGCTGCCGTAGAGGTATGGGA
HPRT	AJ132697	TGGCGATGATGAACAAGGT	GCTACAATGTGGTGTCCTCCC
ITIH5	XM_41729.3	ATGGGGAGGTCACAGGGAAAGAA	TCTCTCTTCCGTTCACAGGGCTT
UPS18	XM_417299.3	TGCTCTCTACTTTCCTTCTCCGCT	ACCACAGGAACATAGGCTGAACCA
GIMAP5	XM_003640651.1	ACCAAACCAGTGACCCTGAG	AGAAGATGTTGGCCGTATCG
KIAA1754	BU420698	GAGCGGCTGCTGGGGGACAT	CCAGCGCGTGGTTTTCCGCA
CCDC79	CR391605.1	AGTGGCTAGAAAGTTGCATGGAGCC	TGCAACCGTGAGGCCAACAAATG
DIO2	NM_204114.1	GCTGGAAGAGTGTTGGTGGCAAG	TGGCTTGTGAATGGTGGTCAGGT
