# Supplementary material for: Dietary Inulin Supplementation Modifies Significantly the Liver Transcriptomic Profile of Broiler Chickens
Source: PLoS One. 2014 Jun 10;9(6):e98942. doi: 10.1371/journal.pone.0098942 (PMC4051581; doi:10.1371/journal.pone.0098942)
Supplement: Table S2 — One hundred and twelve up-regulated genes (≥ 1.4-fold) and 46 down-regulated genes (≤ 0.6-fold) showing a P-value ≤ 0.09 when using an Affymetrix GeneChip Chicken Genome Array in RNA from chicken broilers supplemented with inulin. (PDF) [file pone.0098942.s002.pdf]

**Table S2.** One hundred and twelve up-regulated genes (≥ 1.4-fold) and 46 down-regulated genes (≤ 0.6-fold) showing a *P-value* ≤ 0.09 when using an Affymetrix GeneChip® Chicken Genome Array in chicken broilers supplemented with inulin.

| Probe Set ID | Ratio With vs. Without | *P* | Public ID | Gene Symbol | Gene Name |
|---|---|---|---|---|---|
| GgaAffx.10059.1.S1_at | 1.8 | 0.08 | ENSGALT00000025470.1 | *ADAMTS5* | ADAM metallopeptidase with thrombospondin type 1 motif, 5 (aggrecanase-2) |
| GgaAffx.20483.1.S1_at | 1.5 | 0.03 | CR524132.1 | *ADAMTS5* | ADAM metallopeptidase with thrombospondin type 1 motif, 5 (aggrecanase-2) |
| GgaAffx.20839.1.S1_s_at | 1.4 | 0.00 | CR523776.1 | *ADAMTSL1* | ADAMTS-like 1 |
| GgaAffx.2253.2.S1_s_at | 1.6 | 0.09 | ENSGALT00000005717.1 | *ADC* | arginine decarboxylase |
| Gga.18034.1.S1_at | 1.6 | 0.07 | BU480374 | *AHCTF1* | AT hook containing transcription factor 1 |
| GgaAffx.23623.2.S1_at | 1.6 | 0.02 | ENSGALT00000019050.1 | *AHNAK2* | AHNAK nucleoprotein 2 |
| Gga.5161.1.S1_s_at | 1.5 | 0.09 | CR352367.1 | *ANK3* | ankyrin 3 |
| Gga.9825.2.S1_s_at | 1.8 | 0.01 | BU476146 | *ARMC1* | Armadillo repeat containing 1 |
| Gga.13932.1.S1_s_at | 1.5 | 0.04 | CR353853.1 | *BACH1* | BTB and CNC homology 1, basic leucine zipper transcription factor 1 |
| GgaAffx.22329.1.S1_at | 1.5 | 0.02 | ENSGALT00000012457.1 | *BCL9L* | B-cell CLL/lymphoma 9-like |
| Gga.3693.1.A1_at | 1.5 | 0.07 | AI982495 | *B-G* | MHC B-G antigen |
| Gga.517.1.S1_x_at | 4.5 | 0.09 | CN210876 | *BLB1* | MHC class II antigen B-F minor heavy chain |
| Gga.13275.1.S1_s_at | 1.4 | 0.05 | BU221568 | *BZRAP1 (PRAX1)* | similar to Peripheral-type benzodiazepine receptor-associated protein 1 (PRAX-1) (Peripheral benzodiazepine receptor interacting protein) (PBR-IP) (RIM binding protein 1) (RIM-BP1) |
| GgaAffx.23227.1.A1_at | 1.4 | 0.09 | ENSGALT00000013340.1 | *BZRAP1 (PRAX1)* | Peripheral-type benzodiazepine receptor-associated protein 1 (PRAX-1) (Peripheral benzodiazepine receptor interacting protein) (PBR-IP) (RIM binding protein 1) (RIM-BP1) |
| Gga.16422.1.S1_at | 1.4 | 0.02 | BU113809 | *C10orf72* | Chromosome 10 open reading frame 72 |
| Gga.10903.1.S1_at | 1.4 | 0.09 | BU375078 | *CALB1* | calbindin 1, 28kDa |
| GgaAffx.9152.5.S1_s_at | 1.4 | 0.02 | ENSGALT00000023280.1 | *CASR* | calcium-sensing receptor |
| Gga.10303.1.S1_at | 1.5 | 0.09 | BI067555 | *CAV2* | caveolin 2 |
| Gga.17147.1.S1_s_at | 1.8 | 0.04 | BU309463 | *CCDC127* | Coiled-coil domain containing 127 |
| Gga.4900.5.S1_at | 2.5 | 0.02 | BX935608.2 | *CD69* | CD69 molecule |
| Gga.18993.1.S1_at | 1.6 | 0.02 | BU117403 | *DGKI* | Diacylglycerol kinase, iota |
| Gga.1819.1.S1_at | 4.3 | 0.02 | NM_204114.1 | *DIO2* | Deiodinase, iodothyronine, type II |
| Gga.552.1.S1_at | 1.8 | 0.04 | Y11273.1 | *DIO3* | Deiodinase, iodothyronine, type III |
| GgaAffx.13074.1.S1_at | 1.8 | 0.01 | AJ720971 | *DNAJC3* | DnaJ (Hsp40) homolog, subfamily C, member 3 |
| GgaAffx.8032.1.S1_at | 1.4 | 0.02 | ENSGALT00000020689.1 | *E2F3* | E2F transcription factor 3 |
| Gga.12036.1.S1_at | 1.4 | 0.03 | BU464460 | *EHF* | ets homologous factor |
| GgaAffx.26637.1.S1_at | 1.5 | 0.05 | ENSGALT00000000945.1 | *ERF* | Ets2 repressor factor |

| | | | | | |
|---|---|---|---|---|---|
| Gga.10859.1.S1_at | 1.5 | 0.07 | BX932090.2 | *FAM20C* | similar to family with sequence similarity 20, member C; IMAGE:4942737 |
| GgaAffx.4478.1.S1_at | 1.4 | 0.09 | ENSGALT00000011587.1 | *FAM3D* | family with sequence similarity 3, member D |
| Gga.2448.1.S2_at | 1.5 | 0.04 | NM_205155.1 | *FASN* | fatty acid synthase |
| GgaAffx.26128.1.S1_at | 1.5 | 0.04 | ENSGALT00000006646.1 | *GAA* | glucosidase, alpha; acid (Pompe disease, glycogen storage disease type II) |
| GgaAffx.2586.1.S1_s_at | 1.4 | 0.04 | ENSGALT00000006644.1 | *GAA* | glucosidase, alpha; acid (Pompe disease, glycogen storage disease type II) |
| GgaAffx.9628.1.S1_at | 1.7 | 0.04 | ENSGALT00000024432.1 | *GDA* | guanine deaminase |
| GgaAffx.3548.1.S1_at | 3.2 | 0.00 | ENSGALT00000009063.1 | *GIMAP5* | GTPase, IMAP family member 5 |
| GgaAffx.22932.2.S1_s_at | 1.5 | 0.08 | ENSGALT00000015408.1 | *GOLIM4* | golgi integral membrane protein 4 |
| GgaAffx.8393.3.S1_s_at | 1.4 | 0.05 | ENSGALT00000021537.1 | *GRAMD4* | GRAM domain containing 4 |
| Gga.5680.1.S1_at | 1.6 | 0.08 | BU469684 | *GRID2* | glutamate receptor, ionotropic, delta 2 |
| Gga.17568.1.S1_at | 1.5 | 0.06 | BU463787 | *HECA* | Headcase homolog (Drosophila) |
| Gga.14003.1.A1_at | 1.6 | 0.00 | BU427151 | *IFNGR2* | Interferon gamma receptor 2 |
| Gga.9364.1.S1_at | 1.5 | 0.01 | BX262749 | *IGFBP5* | Insulin-like growth factor binding protein 5 |
| GgaAffx.952.1.S1_at | 1.5 | 0.09 | ENSGALT00000002204.1 | *IGSF9B* | immunoglobulin superfamily, member 9B |
| Gga.16545.1.S1_at | 1.5 | 0.03 | BU295122 | *IL6* | interleukin-6 precursor |
| Gga.15024.1.S1_at | 1.5 | 0.03 | CR386066.1 | *IL6* | interleukin-6 precursor |
| GgaAffx.22269.1.S1_at | 1.6 | 0.02 | ENSGALT00000012179.1 | *INCENP* | Inner centromere protein antigens 135/155kDa |
| Gga.9732.1.S1_at | 2.2 | 0.01 | BU244720 | *ITIH5* | inter-alpha (globulin) inhibitor H5 |
| GgaAffx.4308.1.S1_s_at | 1.5 | 0.03 | ENSGALT00000011162.1 | *ITIH5* | inter-alpha (globulin) inhibitor H5 |
| Gga.18318.1.S1_at | 3.3 | 0.09 | BU420698 | *ITPRIP (KIAA1754)* | Similar to KIAA1754-like |
| GgaAffx.10015.3.S1_s_at | 1.4 | 0.09 | ENSGALT00000025328.1 | *LETM1* | leucine zipper-EF-hand containing transmembrane protein 1 |
| Gga.12344.1.S1_at | 1.4 | 0.04 | BU371716 | *LIMS1 (PINCH)* | LIM and senescent cell antigen-like domains 1 |
| Gga.9481.1.S1_s_at | 1.4 | 0.09 | NM_204932.1 | *LOC395772* | otokeratin |
| GgaAffx.4394.1.S1_at | 1.4 | 0.08 | ENSGALT00000011376.1 | *LOC416622* | similar to putative endoplasmic reticulum protein family member, with at least 7 transmembrane domains, of ancient origin (53.1 kD) (1F495) |
| Gga.6778.2.S1_a_at | 1.4 | 0.02 | BX264871 | *LOC431317* | similar to Scale keratin (S-ker) (sKer) |
| Gga.13496.2.S1_at | 1.5 | 0.01 | CR407424.1 | *LRBA* | LPS-responsive vesicle trafficking, beach and anchor containing, transcript variant X3 |
| Gga.11132.1.S1_at | 1.6 | 0.02 | BU133413 | *LRRC1 (LANO)* | Leucine rich repeat containing 1 |
| Gga.19858.1.S1_at | 1.4 | 0.00 | BU119290 | *MAP3K3* | mitogen-activated protein kinase kinase kinase 3, transcript variant X3 |
| Gga.16473.1.S1_s_at | 1.5 | 0.00 | CR385148.1 | *MAP7D2* | MAP7 domain containing 2 |
| Gga.17974.1.A1_at | 1.4 | 0.08 | BU309450 | *MIB1* | mindbomb E3 ubiquitin protein ligase 1 |
| GgaAffx.10735.1.S1_at | 1.4 | 0.08 | ENSGALT00000027060.1 | *MITD1* | MIT, microtubule interacting and transport, domain containing 1 |
| GgaAffx.23773.1.S1_s_at | 1.7 | 0.09 | ENSGALT00000019702.1 | *MLH1* | mutL homolog 1, colon cancer, nonpolyposis type 2 (E. coli) |
| GgaAffx.751.1.S1_s_at | 1.5 | 0.09 | ENSGALT00000001684.1 | *MMEL1* | membrane metallo-endopeptidase-like 1 |

| | | | | | |
|---|---|---|---|---|---|
| Gga.17872.1.A1_at | 1.6 | 0.02 | BU412788 | *NCOA3* | Nuclear receptor coactivator 3 |
| Gga.16162.1.S1_at | 1.4 | 0.04 | CR387438.1 | *Near ACSL6* | acyl-CoA synthetase long-chain family member 6, transcript variant X5 |
| Gga.13094.1.S1_at | 1.6 | 0.04 | CR352921.1 | *Near COL13A1* | Collagen type XIII alpha 1 |
| Gga.2143.1.A1_at | 1.4 | 0.09 | CR352925.1 | *Near FAM123B* | family with sequence similarity 123B, transcript variant X7 |
| Gga.14140.1.S1_at | 1.5 | 0.09 | BU390066 | *Near FOXP1* | forkhead box P1 |
| Gga.12627.1.S1_at | 1.8 | 0.06 | BU461086 | *Near GDA* | Guanine deaminase |
| Gga.17403.1.S1_at | 1.4 | 0.06 | BU140017 | *Near KLF9* | Kruppel-like factor 9 |
| Gga.1755.1.S1_at | 1.6 | 0.07 | BU411807 | *NEB* | nebulin |
| Gga.17355.1.S1_at | 1.5 | 0.09 | CR390089.1 | *NEBL* | Nebulette |
| GgaAffx.25567.1.S1_at | 1.4 | 0.04 | ENSGALT00000000420.1 | *NEFL* | neurofilament, light polypeptide 68kDa |
| GgaAffx.20871.1.S1_at | 1.5 | 0.02 | CR523744.1 | *NLGN1* | Neuroligin 1 |
| Gga.772.1.S1_at | 1.4 | 0.04 | BI394262 | *NNF1* | Nnf1 protein |
| Gga.135.3.S1_a_at | 1.4 | 0.09 | L11264.1 | *NRG1* | neuregulin 1 |
| GgaAffx.6653.1.S1_at | 1.4 | 0.04 | ENSGALT00000017121.1 | *NRXN3* | neurexin 3 |
| Gga.19916.1.S1_at | 1.6 | 0.06 | CR407206.1 | *NUMB* | Numb homolog (Drosophila) |
| GgaAffx.689.2.S1_s_at | 1.4 | 0.07 | ENSGALT00000001569.1 | *OLFML2A* | olfactomedin-like 2A |
| GgaAffx.6557.1.S1_at | 1.4 | 0.01 | ENSGALT00000016901.1 | *P2RY12* | purinergic receptor P2Y, G-protein coupled, 12 |
| GgaAffx.4460.1.S1_at | 1.7 | 0.07 | ENSGALT00000011542.1 | *PARD3* | Par-3 partitioning defective 3 homolog (C. elegans) |
| Gga.16444.1.S1_at | 1.6 | 0.04 | CR406294.1 | *PDK4* | Pyruvate dehydrogenase kinase, isozyme 4 |
| GgaAffx.2003.1.A1_at | 1.5 | 0.02 | NW_003763668 | *PIK3R4* | phosphoinositide-3-kinase, regulatory subunit 4 |
| GgaAffx.25933.1.S1_at | 1.4 | 0.07 | ENSGALT00000005813.1 | *PITPNC1* | phosphatidylinositol transfer protein, cytoplasmic 1 |
| GgaAffx.5573.2.S1_s_at | 1.4 | 0.01 | ENSGALT00000014420.1 | *PLCB1* | phospholipase C, beta 1 (phosphoinositide-specific) |
| Gga.19875.1.S1_at | 1.8 | 0.05 | CR407079.1 | *PLXNA2* | Plexin A2 |
| GgaAffx.20310.1.S1_at | 1.5 | 0.01 | CR524305.1 | *PLXNA2* | Plexin A2 |
| Gga.14866.1.S1_at | 1.7 | 0.09 | BU309936 | *PPARA* | peroxisome proliferator-activated receptor alpha |
| GgaAffx.21561.1.S1_at | 1.5 | 0.01 | CR523054.1 | *PPP2R3A* | Protein phosphatase 2, regulatory subunit B'', alpha |
| Gga.5846.1.S1_at | 3.8 | 0.07 | BX540658 | *PRR13* | Proline rich 13 |
| GgaAffx.24869.1.S1_s_at | 1.4 | 0.09 | ENSGALT00000026041.1 | *PTP4A3* | protein tyrosine phosphatase type IVA, member 3 |
| Gga.6563.1.A1_at | 1.5 | 0.01 | BX279513 | *RBM15* | RNA binding motif protein 15 |
| GgaAffx.20672.1.S1_at | 1.6 | 0.08 | CR523943.1 | *RCJMB04_2d20 (DVL1)* | dishevelled, dsh homolog 1 (Drosophila) |
| GgaAffx.7921.1.S1_at | 1.4 | 0.09 | ENSGALT00000020397.1 | *RIF1* | RAP1 interacting factor homolog (yeast) |
| GgaAffx.10231.1.S1_at | 1.5 | 0.01 | ENSGALT00000025855.1 | *RRM2B* | ribonucleotide reductase M2 B (TP53 inducible) |
| GgaAffx.1288.1.S1_at | 1.4 | 0.09 | ENSGALT00000003012.1 | *RSPO1* | R-spondin homolog (Xenopus laevis) |
| Gga.12422.1.S1_at | 1.4 | 0.05 | CK613298 | *RUSC2* | RUN and SH3 domain containing 2 |
| GgaAffx.25330.1.S1_at | 1.5 | 0.05 | ENSGALT00000003015.1 | *SAFB2* | scaffold attachment factor B2 |
| GgaAffx.21809.1.S1_s_at | 1.7 | 0.07 | NM_001001773.1 | *SLC24A1* | solute carrier family 24 member 1 |
| Gga.15268.1.S1_at | 1.6 | 0.04 | CR385226.1 | *SLC6A6 (TAUT)* | Solute carrier family 6 member 6 |

| | | | | | |
|---|---|---|---|---|---|
| GgaAffx.22585.1.S1_s_at | 1.4 | 0.01 | ENSGALT00000013738.1 | *SMARCA1* | SWI/SNF related, matrix associated, actin dependent regulator of chromatin, subfamily a, member 1 |
| Gga.10828.1.S1_at | 1.5 | 0.01 | BU382987 | *SMYD3* | SET and MYND domain containing 3 |
| GgaAffx.21582.1.S1_at | 1.5 | 0.01 | CR523033.1 | *SPECC1L (CYTSA)* | sperm antigen with calponin homology and coiled-coil domains 1-like |
| GgaAffx.1735.1.S1_at | 1.5 | 0.01 | ENSGALT00000004177.1 | *SPEG* | SPEG complex locus |
| Gga.17516.1.A1_s_at | 1.4 | 0.09 | CR389420.1 | *SPEN* | Spen homolog, transcriptional regulator (Drosophila) |
| Gga.3672.1.S1_at | 1.9 | 0.04 | NM_205217.1 | *ST3GAL1* | ST3 beta-galactoside alpha-2,3-sialyltransferase 1 |
| Gga.20016.1.S1_at | 1.6 | 0.04 | AY515255.1 | *ST3GAL5* | ST3 beta-galactoside alpha-2,3-sialyltransferase 5 |
| GgaAffx.12279.1.S1_s_at | 1.4 | 0.05 | AJ720176 | *SUCLA2* | succinate-CoA ligase, ADP-forming, beta subunit |
| Gga.5148.1.S1_at | 1.9 | 0.04 | NM_204439.1 | *TNFRSF1B* | Tumor necrosis factor receptor superfamily member 1B |
| Gga.11168.1.S1_at | 1.4 | 0.07 | BU295911 | *TPK1* | thiamin pyrophosphokinase 1, transcript variant X6 |
| Gga.10343.1.S1_a_at | 1.8 | 0.07 | BU246202 | *TPPP* | Tubulin polymerization promoting protein |
| Gga.16816.1.S1_at | 1.5 | 0.02 | CR389338.1 | *TPPP* | Tubulin polymerization promoting protein |
| Gga.19706.1.S1_at | 1.9 | 0.06 | BU241605 | *UPP2* | uridine phosphorylase 2 |
| GgaAffx.23940.1.S1_at | 1.6 | 0.08 | ENSGALT00000020501.1 | *UPP2* | uridine phosphorylase 2 |
| Gga.10373.1.S1_s_at | 1.5 | 0.00 | BU270366 | *USP44* | Ubiquitin specific peptidase 44 |
| GgaAffx.7824.1.S1_at | 1.4 | 0.01 | ENSGALT00000020179.1 | *VOPP1* | vesicular, overexpressed in cancer, prosurvival protein 1, transcript variant X9 |
| Gga.15807.1.S1_at | 1.7 | 0.08 | BU134637 | --- | --- |
| Gga.19573.1.S1_at | 1.5 | 0.08 | BU289520 | --- | --- |
| Gga.15299.1.A1_at | 1.5 | 0.02 | CR385127.1 | --- | --- |
| Gga.14037.1.S1_at | 1.5 | 0.09 | BU121701 | --- | --- |
| Gga.17520.1.S1_at | 1.5 | 0.07 | BU200510 | --- | --- |
| Gga.5404.1.S1_at | 1.4 | 0.09 | BX263367 | --- | --- |
| Gga.10110.1.S1_at | 1.4 | 0.00 | CR386923.1 | --- | --- |
| Gga.10490.1.A1_at | 1.4 | 0.07 | BX933568.1 | --- | --- |
| Gga.2882.2.S1_a_at | 0.6 | 0.00 | BU433069 | *ABCB9* | ATP-binding cassette, sub-family B (MDR/TAP), member 9 |
| Gga.16063.1.S1_s_at | 0.5 | 0.06 | BU325071 | *ABTB1* | ankyrin repeat and BTB (POZ) domain containing 1 |
| GgaAffx.4468.1.S1_at | 0.6 | 0.05 | ENSGALT00000011555.1 | *ACOX2* | acyl-Coenzyme A oxidase 2, branched chain |
| Gga.10636.1.S1_at | 0.6 | 0.04 | BX932098.2 | *C21orf7* | Chromosome 21 open reading frame 7 |
| GgaAffx.7666.1.S1_at | 0.6 | 0.07 | ENSGALT00000019740.1 | *C4H4ORF32* | similar to Chromosome 4 open reading frame 32 |
| GgaAffx.8171.3.S1_s_at | 0.6 | 0.04 | ENSGALT00000021054.1 | *C5orf22* | chromosome 5 open reading frame 22 |
| Gga.6127.2.S1_a_at | 0.5 | 0.06 | BU450627 | *CABC1* | chaperone, ABC1 activity of bc1 complex homolog (S. pombe) |
| Gga.18832.1.S1_at | 0.5 | 0.01 | CR391605.1 | *CCDC79* | Coiled-coil domain containing 79 (CCDC79) |
| Gga.8244.1.S1_at | 0.5 | 0.09 | BX263886 | *CMPK2* | cytidine monophosphate (UMP-CMP) kinase 2, mitochondrial |
| Gga.3025.1.S1_at | 0.5 | 0.01 | BU350875 | *CREB1* | cAMP responsive element binding protein 1 |
| Gga.5397.1.S1_at | 0.6 | 0.09 | BU270074 | *CYP2J2* | cytochrome P450, family 2, subfamily J, polypeptide 2 |
| GgaAffx.22958.1.S1_at | 0.5 | 0.09 | ENSGALT00000015560.1 | *DCAF17* | chromosome 2 open reading frame 37 |

| | | | | | |
|---|---|---|---|---|---|
| GgaAffx.22996.1.S1_at | 0.4 | 0.07 | ENSGALT00000015699.1 | *DDX60* | DEAD (Asp-Glu-Ala-Asp) box polypeptide 60 |
| Gga.11576.1.S1_at | 0.6 | 0.09 | BU389740 | *DNAJC19* | DnaJ (Hsp40) homolog, subfamily C, member 19 |
| Gga.12738.1.S1_at | 0.4 | 0.08 | CN235455 | *DYNLRB2* | dynein, light chain, roadblock-type 2 |
| GgaAffx.8082.2.A1_at | 0.6 | 0.05 | ENSGALT00000020803.1 | *ELOVL2* | elongation of very long chain fatty acids (FEN1/Elo2, SUR4/Elo3, yeast)-like 2 |
| GgaAffx.24481.1.S1_at | 0.6 | 0.09 | ENSGALT00000023622.1 | *GPR98* | G protein-coupled receptor 98 |
| Gga.2263.1.S1_s_at | 0.6 | 0.05 | L15387.2 | *GSTA* | glutathione S-transferase class-alpha |
| GgaAffx.26446.2.S1_s_at | 0.6 | 0.09 | ENSGALT00000008356.1 | *GSTT1* | glutathione S-transferase theta 1 |
| GgaAffx.10817.1.S1_at | 0.6 | 0.02 | ENSGALT00000027297.1 | *HS6ST3* | similar to Heparan sulfate 6-O-sulfotransferase 3 |
| Gga.5441.2.S1_a_at | 0.6 | 0.09 | BX933391.1 | *HTATIP2* | HIV-1 Tat interactive protein 2, 30kDa |
| GgaAffx.21915.1.S1_at | 0.4 | 0.09 | ENSGALT00000010311.1 | *IFIT5* | interferon-induced protein with tetratricopeptide repeats 5 |
| Gga.4832.1.S1_at | 0.6 | 0.09 | BU405376 | *IFITM1* | interferon-induced transmembrane protein 1-like |
| Gga.10961.1.S1_at | 0.6 | 0.00 | BU351116 | *IMMP2L* | IMP2 inner mitochondrial membrane peptidase-like (S. cerevisiae) |
| Gga.9699.4.S1_a_at | 0.6 | 0.08 | BX934714.2 | *JMJD7-PLA2G4B* | JMJD7-PLA2G4B readthrough transcript |
| Gga.16376.1.S1_x_at | 0.6 | 0.09 | CR386477.1 | *LOC101750364* | uncharacterized LOC101750364 |
| GgaAffx.24012.3.S1_s_at | 0.5 | 0.09 | ENSGALT00000020952.1 | *LOC418109* | cystine/glutamate transporter-like |
| Gga.10818.2.S1_a_at | 0.4 | 0.00 | BU417876 | *LOC429567* | similar to protease |
| GgaAffx.25180.2.A1_at | 0.6 | 0.03 | ENSGALT00000027689.1 | *LOC771527* | hypothetical protein LOC771527 |
| Gga.10818.2.S1_s_at | 0.4 | 0.06 | BU417876 | *MGAT5B* | mannosyl (alpha-1,6-)-glycoprotein beta-1,6-N-acetyl-glucosaminyltransferase, isozyme B |
| Gga.131.1.S1_at | 0.5 | 0.09 | NM_204609.1 | *MX1* | myxovirus (influenza virus) resistance 1, interferon-inducible protein p78 (mouse) |
| Gga.14439.1.S1_at | 0.6 | 0.06 | BX933041.2 | *Near NCAPG2* | non-SMC condensin II complex, subunit G2 |
| Gga.10037.1.S1_s_at | 0.5 | 0.04 | BU220239 | *NOXO1* | NADPH oxidase organizer 1 |
| GgaAffx.605.5.S1_at | 0.6 | 0.08 | ENSGALT00000001346.1 | *NRG2* | neuregulin 2 |
| GgaAffx.24202.3.S1_s_at | 0.6 | 0.01 | ENSGALT00000002190.1 | *RANBP10* | RAN binding protein 10 |
| Gga.9636.1.S1_at | 0.6 | 0.04 | BX929784.2 | *SNORA32* | small nucleolar RNA, H/ACA box 32 |
| Gga.16635.1.S1_at | 0.2 | 0.02 | CN236589 | *SPAG4* | sperm associated antigen 4 |
| GgaAffx.7443.1.S1_at | 0.6 | 0.09 | ENSGALT00000019102.1 | *STAP1* | signal transducing adaptor family member 1 |
| GgaAffx.6820.1.S1_at | 0.5 | 0.09 | ENSGALT00000017550.1 | *TMEM195* | transmembrane protein 195 |
| GgaAffx.3790.1.S1_at | 0.6 | 0.05 | ENSGALT00000009813.1 | *TMPRSS12* | transmembrane protease, serine 12 |
| GgaAffx.9959.1.S1_s_at | 0.6 | 0.04 | ENSGALT00000025204.1 | *TRPA1* | transient receptor potential cation channel, subfamily A, member 1 |
| GgaAffx.25266.1.S1_at | 0.6 | 0.08 | ENSGALT00000023473.1 | *TTC38* | tetratricopeptide repeat protein 38-like |
| Gga.1111.1.S1_a_at | 0.5 | 0.03 | CR389337.1 | *USP18* | ubiquitin specific peptidase 18 |
| GgaAffx.10545.1.S1_at | 0.6 | 0.06 | ENSGALT00000026579.1 | *XPAX* | similar to egg envelope component ZPAX |
| NC-001720.CDS25.S1_at | 0.6 | 0.03 | --- | --- | --- |
| GgaAffx.4165.1.S1_at | 0.4 | 0.02 | ENSGALT00000010815.1 | --- | --- |
