# Supplementary material for: Dietary Inulin Supplementation Modifies Significantly the Liver Transcriptomic Profile of Broiler Chickens
Source: PLoS One. 2014 Jun 10;9(6):e98942. doi: 10.1371/journal.pone.0098942 (PMC4051581; doi:10.1371/journal.pone.0098942)
Supplement: Table S3 — Gene list report and complete results for the 95 up-regulated and 35 down-regulated genes for which annotation information was available at DAVID Bioinformatics Resources 6.7 ( http://david.abcc.ncifcrf.gov/ ) in August 2013, including the corresponding DAVID scores ( P values ) and the lists of genes in every significant category. A summary of the Functional Annotation Clusters (FAC) is also shown from page 108 to 112. (PDF) [file pone.0098942.s003.pdf]

Table S3. Gene list report and complete results for the 95 up-regulated and 35 down-regulated genes for which annotation information was available at DAVID Bioinformatics Resources 6.7 (http://david.abcc.ncifcrf.gov/) in August 2013, including the corresponding DAVID scores (P values) and the lists of genes in every significant category. A summary of the Functional Annotation Clusters (FAC) is also shown from page 108 to 112.

Expression	GENE SYMBOL[1]	Gene Name
Up Regulated	ACSL6	similar to fatty acid Coenzyme A ligase, long chain 6; acyl-CoA synthetase long-chain family member 6
Up Regulated	ADAMTS5	ADAM metallopeptidase with thrombospondin type 1 motif, 5
Up Regulated	ADAMTSL1	ADAMTS-like 1
Up Regulated	ADC	arginine decarboxylase
Up Regulated	AHCTF1	AT hook containing transcription factor 1; AT hook containing transcription factor 1 pseudogene
Up Regulated	AHNAK2	AHNAK nucleoprotein 2
Up Regulated	ANK3	ankyrin 3, node of Ranvier (ankyrin G)
Up Regulated	ARMC1	armadillo repeat containing 1
Up Regulated	BACH1	BTB and CNC homology 1, basic leucine zipper transcription factor 1
Up Regulated	BCL9L	B-cell CLL/lymphoma 9-like
Up Regulated	BLB1	MHC class II antigen B-F minor heavy chain; MHC class II beta chain
Up Regulated	BZRAP1	benzodiazapine receptor (peripheral) associated protein 1
Up Regulated	C10orf72	chromosome 10 open reading frame 72
Up Regulated	CALB1	calbindin 1, 28kDa
Up Regulated	CASR	calcium-sensing receptor
Up Regulated	CAV2	caveolin 2
Up Regulated	CCDC127	coiled-coil domain containing 127
Up Regulated	CD69	CD69 molecule
Up Regulated	COL13A1	collagen, type XIII, alpha 1
Up Regulated	DGKI	diacylglycerol kinase, iota
Up Regulated	DIO2	deiodinase, iodothyronine, type II
Up Regulated	DIO3	deiodinase, iodothyronine, type III
Up Regulated	DNAJC3	DnaJ (Hsp40) homolog, subfamily C, member 3
Up Regulated	DVL1	dishevelled, dsh homolog 1 (Drosophila); dishevelled, dsh homolog 1 (Drosophila)-like 1
Up Regulated	E2F3	E2F transcription factor 3
Up Regulated	EHF	ets homologous factor

Up Regulated	ERF	Ets2 repressor factor
Up Regulated	FAM123B	family with sequence similarity 123B
Up Regulated	FAM20C	family with sequence similarity 20, member C
Up Regulated	FAM3D	family with sequence similarity 3, member D
Up Regulated	FASN	fatty acid synthase
Up Regulated	FOXP1	forkhead box P1
Up Regulated	GAA	glucosidase, alpha; acid
Up Regulated	GDA	guanine deaminase
Up Regulated	GIMAP5	GTPase, IMAP family member 5
Up Regulated	GOLIM4	golgi integral membrane protein 4
Up Regulated	GRAMD4	TSC22 domain family, member 3; GRAM domain containing 4
Up Regulated	GRID2	glutamate receptor, ionotropic, delta 2
Up Regulated	HECA	headcase homolog (Drosophila)
Up Regulated	IFNGR2	interferon gamma receptor 2 (interferon gamma transducer 1)
Up Regulated	IGFBP5	insulin-like growth factor binding protein 5
Up Regulated	IGSF9B	immunoglobulin superfamily, member 9B
Up Regulated	IL6	interleukin 6 (interferon, beta 2)
Up Regulated	INCENP	inner centromere protein antigens 135/155kDa
Up Regulated	ITIH5	inter-alpha (globulin) inhibitor H5
Up Regulated	ITPRIP	inositol 1,4,5-triphosphate receptor interacting protein
Up Regulated	KLF9	Kruppel-like factor 9
Up Regulated	LETM1	leucine zipper-EF-hand containing transmembrane protein 1
Up Regulated	LIMS1	LIM and senescent cell antigen-like domains 1
Up Regulated	LRBA	LPS-responsive vesicle trafficking, beach and anchor containing
Up Regulated	LRRC1	leucine rich repeat containing 1
Up Regulated	MAP3K3	mitogen-activated protein kinase kinase kinase 3
Up Regulated	MAP7D2	MAP7 domain containing 2
Up Regulated	MIB1	mindbomb homolog 1 (Drosophila)
Up Regulated	MITD1	MIT, microtubule interacting and transport, domain containing 1
Up Regulated	MLH1	mutL homolog 1, colon cancer, nonpolyposis type 2 (E. coli)
Up Regulated	MMEL1	membrane metallo-endopeptidase-like 1
Up Regulated	NCOA3	nuclear receptor coactivator 3
Up Regulated	NEB	nebulin

Up Regulated	NEBL	nebulette
Up Regulated	NEFL	neurofilament, light polypeptide
Up Regulated	NLGN1	neuroligin 1
Up Regulated	NRG1	neuregulin 1
Up Regulated	NRXN3	neurexin 3
Up Regulated	NUMB	numb homolog (Drosophila)
Up Regulated	OLFML2A	olfactomedin-like 2A
Up Regulated	P2RY12	purinergic receptor P2Y, G-protein coupled, 12
Up Regulated	PARD3	par-3 partitioning defective 3 homolog (C. elegans)
Up Regulated	PDK4	pyruvate dehydrogenase kinase, isozyme 4
Up Regulated	PIK3R4	phosphoinositide-3-kinase, regulatory subunit 4
Up Regulated	PITPNC1	phosphatidylinositol transfer protein, cytoplasmic 1
Up Regulated	PLCB1	phospholipase C, beta 1 (phosphoinositide-specific)
Up Regulated	PLXNA2	plexin A2
Up Regulated	PPARA	peroxisome proliferator-activated receptor alpha
Up Regulated	PPP2R3A	protein phosphatase 2 (formerly 2A), regulatory subunit B'', alpha
Up Regulated	PRR13	proline rich 13
Up Regulated	PTP4A3	protein tyrosine phosphatase type IVA, member 3
Up Regulated	RBM15	RNA binding motif protein 15
Up Regulated	RIF1	RAP1 interacting factor homolog (yeast)
Up Regulated	RRM2B	ribonucleotide reductase M2 B (TP53 inducible)
Up Regulated	RSPO1	R-spondin homolog (Xenopus laevis)
Up Regulated	RUSC2	RUN and SH3 domain containing 2
Up Regulated	SAFB2	scaffold attachment factor B2
Up Regulated	SLC24A1	solute carrier family 24 (sodium/potassium/calcium exchanger), member 1
Up Regulated	SLC6A6	solute carrier family 6 (neurotransmitter transporter, taurine), member 6
Up Regulated	SMARCA1	SWI/SNF related, matrix associated, actin dependent regulator of chromatin, subfamily a, member 1
Up Regulated	SMYD3	SET and MYND domain containing 3
Up Regulated	SPEG	SPEG complex locus
Up Regulated	SPEN	spen homolog, transcriptional regulator (Drosophila)
Up Regulated	ST3GAL1	ST3 beta-galactoside alpha-2,3-sialyltransferase 1
Up Regulated	ST3GAL5	ST3 beta-galactoside alpha-2,3-sialyltransferase 5
Up Regulated	SUCLA2	succinate-CoA ligase, ADP-forming, beta subunit

Up Regulated	TNFRSF1B	tumor necrosis factor receptor superfamily, member 1B
Up Regulated	TPK1	thiamin pyrophosphokinase 1
Up Regulated	TPPP	tubulin polymerization promoting protein
Up Regulated	UPP2	uridine phosphorylase 2
Up Regulated	USP44	ubiquitin specific peptidase 44
Up Regulated	VOPP1	similar to EGFR-coamplified and overexpressed protein; EGFR-coamplified and overexpressed protein
Down Regulated	ABCB9	ATP-binding cassette, sub-family B (MDR/TAP), member 9
Down Regulated	ABTB1	ankyrin repeat and BTB (POZ) domain containing 1
Down Regulated	ACOX2	acyl-Coenzyme A oxidase 2, branched chain
Down Regulated	C21orf7	chromosome 21 open reading frame 7
Down Regulated	C5orf22	chromosome 5 open reading frame 22
Down Regulated	CABC1	chaperone, ABC1 activity of bc1 complex homolog (S. pombe)
Down Regulated	CCDC79	coiled-coil domain containing 79
Down Regulated	CMPK2	cytidine monophosphate (UMP-CMP) kinase 2, mitochondrial
Down Regulated	CREB1	cAMP responsive element binding protein 1
Down Regulated	CYP2J2	hypothetical protein LOC776183; cytochrome P450, family 2, subfamily J, polypeptide 2
Down Regulated	DCAF17	hypothetical protein LOC776754; chromosome 2 open reading frame 37; hypothetical protein LOC777468
Down Regulated	DDX60	DEAD (Asp-Glu-Ala-Asp) box polypeptide 60
Down Regulated	DNAJC19	similar to translocase of the inner mitochondrial membrane 14; DnaJ (Hsp40) homolog, subfamily C, member 19
Down Regulated	DYNLRB2	dynein, light chain, roadblock-type 2; hypothetical protein LOC770420
Down Regulated	ELOVL2	elongation of very long chain fatty acids (FEN1/Elo2, SUR4/Elo3, yeast)-like 2
Down Regulated	GPR98	G protein-coupled receptor 98
Down Regulated	GSTA	glutathione S-transferase class-alpha
Down Regulated	GSTT1	glutathione S-transferase theta 1
Down Regulated	HS6ST3	heparan sulfate 6-O-sulfotransferase 3
Down Regulated	HTATIP2	HIV-1 Tat interactive protein 2, 30kDa
Down Regulated	IFIT5	interferon-induced protein with tetratricopeptide repeats 5
Down Regulated	IFITM1	interferon induced transmembrane protein 1 (9-27)
Down Regulated	IMMP2L	IMP2 inner mitochondrial membrane peptidase-like (S. cerevisiae)
Down Regulated	JMJD7-PLA2G4B	JMJD7-PLA2G4B readthrough transcript
Down Regulated	LOC418109	hypothetical LOC418109
Down Regulated	LOC429567	similar to protease
Down Regulated	LOC771527	hypothetical protein LOC771527

Down Regulated	MGAT5B	mannosyl (alpha-1,6-)-glycoprotein beta-1,6-N-acetyl-glucosaminyltransferase, isozyme B
Down Regulated	MX1	myxovirus (influenza virus) resistance 1, interferon-inducible protein p78 (mouse)
Down Regulated	NCAPG2	non-SMC condensin II complex, subunit G2
Down Regulated	NOXO1	NADPH oxidase organizer 1
Down Regulated	NRG2	neuregulin 2
Down Regulated	RANBP10	RAN binding protein 10
Down Regulated	SNORA32	small nucleolar RNA, H/ACA box 32
Down Regulated	SPAG4	sperm associated antigen 4
Down Regulated	STAP1	signal transducing adaptor family member 1
Down Regulated	TMEM195	transmembrane protein 195
Down Regulated	TMPRSS12	transmembrane protease, serine 12
Down Regulated	TRPA1	transient receptor potential cation channel, subfamily A, member 1
Down Regulated	TTC38	hypothetical protein LOC776175; tetratricopeptide repeat domain 38; similar to FLJ20699 protein
Down Regulated	USP18	ubiquitin specific peptidase 18

[1] In dark: genes for which the FAC analysis produced enriched functional clusters under high stringency conditions

Functional Annotation of Up-regulated Genes

**Annotation Cluster 1**	**Development**	**Enrichment Score: 3,781434603151875**			
Category	Term	Count	%	PValue	Genes
GOTERM_BP_ALL	GO:0048731~system development	29	30	7,29E-05	PPARA, CAV2, GDA, PARD3, CASR, PLXNA2, MLH1, EHF, RRM2B, HECA, SPEG, ANK3, NUMB, AHNAK2, NRG1, NEFL, ACSL6, IL6, GIMAP5, COL13A1, NRXN3, NLGN1, FOXP1, DVL1, MIB1, NEB, GAA, SMARCA1, IGFBP5
GOTERM_BP_ALL	GO:0048856~anatomical structure development	30	31	1,19E-04	PPARA, CAV2, GDA, PARD3, CASR, LIMS1, PLXNA2, MLH1, EHF, RRM2B, HECA, ANK3, SPEG, NUMB, AHNAK2, NRG1, NEFL, ACSL6, IL6, GIMAP5, COL13A1, NRXN3, NLGN1, FOXP1, DVL1, MIB1, NEB, GAA, SMARCA1, IGFBP5
GOTERM_BP_ALL	GO:0007275~multicellular organismal development	32	33	1,88E-04	PPARA, CAV2, GDA, PARD3, CASR, LIMS1, PLXNA2, AHCTF1, MLH1, EHF, RRM2B, HECA, ANK3, SPEG, NUMB, AHNAK2, NRG1, NEFL, ACSL6, IL6, GIMAP5, KLF9, COL13A1, NRXN3, NLGN1, FOXP1, DVL1, MIB1, NEB, GAA, SMARCA1, IGFBP5
GOTERM_BP_ALL	GO:0032502~developmental process	33	34	4,59E-04	PPARA, CAV2, GDA, PARD3, CASR, LIMS1, PLXNA2, AHCTF1, MLH1, EHF, RRM2B, HECA, ANK3, SPEG, NUMB, AHNAK2, NRG1, NEFL, ACSL6, IL6, GIMAP5, KLF9, COL13A1, NRXN3, NLGN1, FOXP1, DVL1, MIB1, RIF1, NEB, GAA, SMARCA1, IGFBP5

**Annotation Cluster 2**	**Muscle development**	**Enrichment Score: 3,2976566653643413**			
Category	Term	Count	%	PValue	Genes
GOTERM_BP_ALL	GO:0007517~muscle organ development	8	8	2,22E-04	CAV2, NEB, SPEG, GAA, NRG1, FOXP1, DVL1, IGFBP5
GOTERM_BP_FAT	GO:0007517~muscle organ development	8	8	2,46E-04	CAV2, NEB, SPEG, GAA, NRG1, FOXP1, DVL1, IGFBP5
GOTERM_BP_ALL	GO:0014706~striated muscle tissue development	6	6	6,36E-04	CAV2, GAA, NRG1, FOXP1, DVL1, IGFBP5
GOTERM_BP_FAT	GO:0014706~striated muscle tissue development	6	6	6,88E-04	CAV2, GAA, NRG1, FOXP1, DVL1, IGFBP5
GOTERM_BP_ALL	GO:0060537~muscle tissue development	6	6	7,95E-04	CAV2, GAA, NRG1, FOXP1, DVL1, IGFBP5
GOTERM_BP_FAT	GO:0060537~muscle tissue development	6	6	8,59E-04	CAV2, GAA, NRG1, FOXP1, DVL1, IGFBP5

**Annotation Cluster 3**	**Nervous system development**	**Enrichment Score: 3,0069772855655397**			
Category	Term	Count	%	PValue	Genes
GOTERM_BP_ALL	GO:0048699~generation of neurons	12	12	3,72E-04	MIB1, CAV2, PARD3, IL6, NRXN3, ANK3, NUMB, NLGN1, SMARCA1, NEFL, ACSL6, DVL1

GOTERM_BP_ALL	GO:0022008~neurogenesis	12	12	6,82E-04	MIB1, CAV2, PARD3, IL6, NRXN3, ANK3, NUMB, NLGN1, SMARCA1, NEFL, ACSL6, DVL1
GOTERM_BP_ALL	GO:0007399~nervous system development	15	15	0,004	CAV2, IL6, PARD3, GDA, NRXN3, PLXNA2, NLGN1, DVL1, MIB1, ANK3, NUMB, SMARCA1, NRG1, NEFL, ACSL6

**Annotation Cluster 4**	**Skeletal muscle development**	**Enrichment Score: 2,1669904987561552**			
Category	Term	Count	%	PValue	Genes
GOTERM_BP_ALL	GO:0060538~skeletal muscle organ development	4	4	0,007	CAV2, FOXP1, DVL1, IGFBP5
GOTERM_BP_ALL	GO:0007519~skeletal muscle tissue development	4	4	0,007	CAV2, FOXP1, DVL1, IGFBP5
GOTERM_BP_FAT	GO:0007519~skeletal muscle tissue development	4	4	0,007	CAV2, FOXP1, DVL1, IGFBP5
GOTERM_BP_FAT	GO:0060538~skeletal muscle organ development	4	4	0,007	CAV2, FOXP1, DVL1, IGFBP5

**Annotation Cluster 5**	**Muscle cell differentiation**	**Enrichment Score: 2,0417968938924353**			
Category	Term	Count	%	PValue	Genes
GOTERM_BP_ALL	GO:0042692~muscle cell differentiation	5	5	0,005	CAV2, SPEG, NRG1, FOXP1, DVL1
GOTERM_BP_FAT	GO:0042692~muscle cell differentiation	5	5	0,006	CAV2, SPEG, NRG1, FOXP1, DVL1
GOTERM_BP_ALL	GO:0051146~striated muscle cell differentiation	4	4	0,015	CAV2, NRG1, FOXP1, DVL1
GOTERM_BP_FAT	GO:0051146~striated muscle cell differentiation	4	4	0,015	CAV2, NRG1, FOXP1, DVL1

**Annotation Cluster 6**	**Protein metabolism**	**Enrichment Score: 1,8972999236158714**			
Category	Term	Count	%	PValue	Genes
GOTERM_BP_ALL	GO:0043623~cellular protein complex assembly	7	7	0,000	CAV2, TPPP, MLH1, AHCTF1, NRG1, NEFL, DVL1
GOTERM_BP_FAT	GO:0043623~cellular protein complex assembly	7	7	0,000	CAV2, TPPP, MLH1, AHCTF1, NRG1, NEFL, DVL1
GOTERM_BP_ALL	GO:0070271~protein complex biogenesis	9	9	0,009	CAV2, E2F3, PARD3, TPPP, MLH1, AHCTF1, NRG1, NEFL, DVL1
GOTERM_BP_ALL	GO:0006461~protein complex assembly	9	9	0,009	CAV2, E2F3, PARD3, TPPP, MLH1, AHCTF1, NRG1, NEFL, DVL1
GOTERM_BP_FAT	GO:0070271~protein complex biogenesis	9	9	0,010	CAV2, E2F3, PARD3, TPPP, MLH1, AHCTF1, NRG1, NEFL, DVL1

GOTERM_BP_FAT	GO:0006461~protein complex assembly	9	9	0,010	CAV2, E2F3, PARD3, TPPP, MLH1, AHCTF1, NRG1, NEFL, DVL1
GOTERM_BP_ALL	GO:0034622~cellular macromolecular complex assembly	7	7	0,010	CAV2, TPPP, MLH1, AHCTF1, NRG1, NEFL, DVL1
GOTERM_BP_FAT	GO:0034622~cellular macromolecular complex assembly	7	7	0,011	CAV2, TPPP, MLH1, AHCTF1, NRG1, NEFL, DVL1
GOTERM_BP_ALL	GO:0034621~cellular macromolecular complex subunit organization	7	7	0,018	CAV2, TPPP, MLH1, AHCTF1, NRG1, NEFL, DVL1
GOTERM_BP_FAT	GO:0034621~cellular macromolecular complex subunit organization	7	7	0,019	CAV2, TPPP, MLH1, AHCTF1, NRG1, NEFL, DVL1
GOTERM_BP_ALL	GO:0022607~cellular component assembly	11	11	0,032	CAV2, E2F3, PARD3, NRXN3, TPPP, NLGN1, MLH1, AHCTF1, NRG1, NEFL, DVL1
GOTERM_BP_ALL	GO:0065003~macromolecular complex assembly	9	9	0,039	CAV2, E2F3, PARD3, TPPP, MLH1, AHCTF1, NRG1, NEFL, DVL1
GOTERM_BP_FAT	GO:0065003~macromolecular complex assembly	9	9	0,042	CAV2, E2F3, PARD3, TPPP, MLH1, AHCTF1, NRG1, NEFL, DVL1
GOTERM_BP_ALL	GO:0043933~macromolecular complex subunit organization	9	9	0,054	CAV2, E2F3, PARD3, TPPP, MLH1, AHCTF1, NRG1, NEFL, DVL1
GOTERM_BP_FAT	GO:0043933~macromolecular complex subunit organization	9	9	0,058	CAV2, E2F3, PARD3, TPPP, MLH1, AHCTF1, NRG1, NEFL, DVL1
GOTERM_BP_ALL	GO:0044085~cellular component biogenesis	11	11	0,064	CAV2, E2F3, PARD3, NRXN3, TPPP, NLGN1, MLH1, AHCTF1, NRG1, NEFL, DVL1

**Annotation Cluster 7**	**Neuron development**	**Enrichment Score: 1,8574100277574226**			
Category	Term	Count	%	PValue	Genes
GOTERM_BP_ALL	GO:0031175~neuron projection development	7	7	0,004	PARD3, IL6, NRXN3, ANK3, NUMB, NEFL, DVL1
GOTERM_BP_FAT	GO:0031175~neuron projection development	7	7	0,004	PARD3, IL6, NRXN3, ANK3, NUMB, NEFL, DVL1
GOTERM_BP_ALL	GO:0048667~cell morphogenesis involved in neuron differentiation	6	6	0,007	PARD3, NRXN3, ANK3, NUMB, NEFL, DVL1
GOTERM_BP_FAT	GO:0048667~cell morphogenesis involved in neuron differentiation	6	6	0,008	PARD3, NRXN3, ANK3, NUMB, NEFL, DVL1
GOTERM_BP_ALL	GO:0048812~neuron projection morphogenesis	6	6	0,008	PARD3, NRXN3, ANK3, NUMB, NEFL, DVL1
GOTERM_BP_FAT	GO:0048812~neuron projection morphogenesis	6	6	0,009	PARD3, NRXN3, ANK3, NUMB, NEFL, DVL1

GOTERM_BP_ALL	GO:0030182~neuron differentiation	8	8	0,014	PARD3, IL6, NRXN3, ANK3, NUMB, SMARCA1, NEFL, DVL1
GOTERM_BP_ALL	GO:0000904~cell morphogenesis involved in differentiation	6	6	0,014	PARD3, NRXN3, ANK3, NUMB, NEFL, DVL1
GOTERM_BP_ALL	GO:0048666~neuron development	7	7	0,014	PARD3, IL6, NRXN3, ANK3, NUMB, NEFL, DVL1
GOTERM_BP_ALL	GO:0048858~cell projection morphogenesis	6	6	0,014	PARD3, NRXN3, ANK3, NUMB, NEFL, DVL1
GOTERM_BP_FAT	GO:0000904~cell morphogenesis involved in differentiation	6	6	0,015	PARD3, NRXN3, ANK3, NUMB, NEFL, DVL1
GOTERM_BP_FAT	GO:0030182~neuron differentiation	8	8	0,015	PARD3, IL6, NRXN3, ANK3, NUMB, SMARCA1, NEFL, DVL1
GOTERM_BP_FAT	GO:0048858~cell projection morphogenesis	6	6	0,015	PARD3, NRXN3, ANK3, NUMB, NEFL, DVL1
GOTERM_BP_FAT	GO:0048666~neuron development	7	7	0,015	PARD3, IL6, NRXN3, ANK3, NUMB, NEFL, DVL1
GOTERM_BP_ALL	GO:0032990~cell part morphogenesis	6	6	0,017	PARD3, NRXN3, ANK3, NUMB, NEFL, DVL1
GOTERM_BP_ALL	GO:0000902~cell morphogenesis	7	7	0,017	PARD3, LIMS1, NRXN3, ANK3, NUMB, NEFL, DVL1
GOTERM_BP_FAT	GO:0032990~cell part morphogenesis	6	6	0,018	PARD3, NRXN3, ANK3, NUMB, NEFL, DVL1
GOTERM_BP_FAT	GO:0000902~cell morphogenesis	7	7	0,019	PARD3, LIMS1, NRXN3, ANK3, NUMB, NEFL, DVL1
GOTERM_BP_ALL	GO:0030030~cell projection organization	7	7	0,020	PARD3, IL6, NRXN3, ANK3, NUMB, NEFL, DVL1
GOTERM_BP_FAT	GO:0030030~cell projection organization	7	7	0,022	PARD3, IL6, NRXN3, ANK3, NUMB, NEFL, DVL1
GOTERM_BP_ALL	GO:0007409~axonogenesis	5	5	0,026	PARD3, NRXN3, ANK3, NUMB, NEFL
GOTERM_BP_FAT	GO:0007409~axonogenesis	5	5	0,027	PARD3, NRXN3, ANK3, NUMB, NEFL
GOTERM_BP_ALL	GO:0032989~cellular component morphogenesis	7	7	0,028	PARD3, LIMS1, NRXN3, ANK3, NUMB, NEFL, DVL1
GOTERM_BP_FAT	GO:0032989~cellular component morphogenesis	7	7	0,030	PARD3, LIMS1, NRXN3, ANK3, NUMB, NEFL, DVL1

**Annotation Cluster 8**	**Neuron signaling**	**Enrichment Score: 1,5433525723979065**			
Category	Term	Count	%	PValue	Genes
GOTERM_BP_ALL	GO:0050808~synapse organization	4	4	0,005	NRXN3, ANK3, NLGN1, DVL1
GOTERM_BP_FAT	GO:0050808~synapse organization	4	4	0,006	NRXN3, ANK3, NLGN1, DVL1
GOTERM_BP_ALL	GO:0006836~neurotransmitter transport	4	4	0,012	NRXN3, SLC6A6, NLGN1, DVL1
GOTERM_BP_FAT	GO:0006836~neurotransmitter transport	4	4	0,013	NRXN3, SLC6A6, NLGN1, DVL1
GOTERM_BP_ALL	GO:0003001~generation of a signal involved in cell-cell signaling	4	4	0,013	IL6, NRXN3, NLGN1, DVL1
GOTERM_BP_FAT	GO:0003001~generation of a signal involved in cell-cell signaling	4	4	0,014	IL6, NRXN3, NLGN1, DVL1
GOTERM_BP_ALL	GO:0043062~extracellular structure organization	5	5	0,015	NRXN3, ANK3, NLGN1, OLFML2A, DVL1

GOTERM_BP_FAT	GO:0043062~extracellular structure organization	5	5	0,016	NRXN3, ANK3, NLGN1, OLFML2A, DVL1
GOTERM_BP_ALL	GO:0007269~neurotransmitter secretion	3	3	0,017	NRXN3, NLGN1, DVL1
GOTERM_BP_FAT	GO:0007269~neurotransmitter secretion	3	3	0,017	NRXN3, NLGN1, DVL1
GOTERM_BP_ALL	GO:0001505~regulation of neurotransmitter levels	3	3	0,058	NRXN3, NLGN1, DVL1
GOTERM_BP_FAT	GO:0001505~regulation of neurotransmitter levels	3	3	0,060	NRXN3, NLGN1, DVL1
GOTERM_BP_ALL	GO:0032940~secretion by cell	4	4	0,119	IL6, NRXN3, NLGN1, DVL1
GOTERM_BP_FAT	GO:0032940~secretion by cell	4	4	0,124	IL6, NRXN3, NLGN1, DVL1
GOTERM_BP_ALL	GO:0046903~secretion	4	4	0,253	IL6, NRXN3, NLGN1, DVL1
GOTERM_BP_FAT	GO:0046903~secretion	4	4	0,262	IL6, NRXN3, NLGN1, DVL1

**Annotation Cluster 9**	**Intracellular organelle**	**Enrichment Score: 1,5306430079180702**			
Category	Term	Count	%	PValue	Genes
GOTERM_CC_ALL	GO:0043229~intracellular organelle	61	63	0,016	BACH1, PPARA, E2F3, MLH1, MITD1, CALB1, ST3GAL1, RSPO1, ST3GAL5, ANK3, INCENP, GRID2, PLCB1, SUCLA2, NRG1, DNAJC3, IFNGR2, MMEL1, SPEN, FAM123B, MIB1, LETM1, NCOA3, RIF1, TPPP, GAA, VOPP1, SMARCA1, ADC, CAV2, PARD3, LRBA, AHCTF1, UPP2, EHF, RRM2B, GRAMD4, SPEG, NUMB, FASN, AHNAK2, BCL9L, NEFL, ACSL6, ERF, GIMAP5, KLF9, PDK4, NLGN1, BZRAP1, SMYD3, GOLIM4, DGKI, FOXP1, SAFB2, DVL1, NEB, PTP4A3, DIO3, RBM15, USP44
GOTERM_CC_ALL	GO:0043226~organelle	61	63	0,017	BACH1, PPARA, E2F3, MLH1, MITD1, CALB1, ST3GAL1, RSPO1, ST3GAL5, ANK3, INCENP, GRID2, PLCB1, SUCLA2, NRG1, DNAJC3, IFNGR2, MMEL1, SPEN, FAM123B, MIB1, LETM1, NCOA3, RIF1, TPPP, GAA, VOPP1, SMARCA1, ADC, CAV2, PARD3, LRBA, AHCTF1, UPP2, EHF, RRM2B, GRAMD4, SPEG, NUMB, FASN, AHNAK2, BCL9L, NEFL, ACSL6, ERF, GIMAP5, KLF9, PDK4, NLGN1, BZRAP1, SMYD3, GOLIM4, DGKI, FOXP1, SAFB2, DVL1, NEB, PTP4A3, DIO3, RBM15, USP44

GOTERM_CC_ALL	GO:0043231~intracellular membrane-bounded organelle	54	56	0,037	BACH1, PPARA, E2F3, MLH1, MITD1, CALB1, ST3GAL1, RSPO1, ST3GAL5, ANK3, INCENP, SUCLA2, PLCB1, NRG1, DNAJC3, IFNGR2, MMEL1, SPEN, FAM123B, LETM1, NCOA3, RIF1, TPPP, GAA, VOPP1, SMARCA1, ADC, CAV2, LRBA, AHCTF1, EHF, RRM2B, GRAMD4, SPEG, NUMB, FASN, AHNAK2, BCL9L, ACSL6, ERF, GIMAP5, KLF9, PDK4, SMYD3, GOLIM4, BZRAP1, DGKI, FOXP1, SAFB2, DVL1, PTP4A3, DIO3, RBM15, USP44
GOTERM_CC_ALL	GO:0043227~membrane-bounded organelle	54	56	0,038	BACH1, PPARA, E2F3, MLH1, MITD1, CALB1, ST3GAL1, RSPO1, ST3GAL5, ANK3, INCENP, SUCLA2, PLCB1, NRG1, DNAJC3, IFNGR2, MMEL1, SPEN, FAM123B, LETM1, NCOA3, RIF1, TPPP, GAA, VOPP1, SMARCA1, ADC, CAV2, LRBA, AHCTF1, EHF, RRM2B, GRAMD4, SPEG, NUMB, FASN, AHNAK2, BCL9L, ACSL6, ERF, GIMAP5, KLF9, PDK4, SMYD3, GOLIM4, BZRAP1, DGKI, FOXP1, SAFB2, DVL1, PTP4A3, DIO3, RBM15, USP44
GOTERM_CC_ALL	GO:0044424~intracellular part	67	69	0,061	BACH1, PPARA, E2F3, GDA, RUSC2, MLH1, MITD1, CALB1, ST3GAL1, RSPO1, ST3GAL5, ANK3, INCENP, GRID2, PITPNC1, DNAJC3, PLCB1, SUCLA2, NRG1, IFNGR2, MMEL1, LRRC1, SPEN, FAM123B, MIB1, LETM1, NCOA3, RIF1, TPPP, GAA, VOPP1, SMARCA1, ADC, CAV2, PARD3, PPP2R3A, LRBA, AHCTF1, UPP2, EHF, RRM2B, GRAMD4, SPEG, NUMB, FASN, AHNAK2, BCL9L, NEFL, PIK3R4, ACSL6, ERF, GIMAP5, KLF9, PDK4, NLGN1, BZRAP1, SMYD3, GOLIM4, DGKI, FOXP1, SAFB2, DVL1, NEB, PTP4A3, DIO3, RBM15, USP44

**Annotation Cluster 10**	**Plasma membrane**	**Enrichment Score: 1,293625363137786**			
Category	Term	Count	%	PValue	Genes
GOTERM_CC_ALL	GO:0031226~intrinsic to plasma membrane	13	13	0,035	CAV2, IL6, PARD3, CASR, NRXN3, NLGN1, ST3GAL5, CD69, SLC6A6, NUMB, SLC24A1, GRID2, IFNGR2
GOTERM_CC_FAT	GO:0031226~intrinsic to plasma membrane	13	13	0,042	CAV2, IL6, PARD3, CASR, NRXN3, NLGN1, ST3GAL5, CD69, SLC6A6, NUMB, SLC24A1, GRID2, IFNGR2
GOTERM_CC_ALL	GO:0005887~integral to plasma membrane	12	12	0,063	CAV2, CASR, IL6, ST3GAL5, NRXN3, CD69, SLC24A1, NUMB, SLC6A6, NLGN1, GRID2, IFNGR2
GOTERM_CC_FAT	GO:0005887~integral to plasma membrane	12	12	0,073	CAV2, CASR, IL6, ST3GAL5, NRXN3, CD69, SLC24A1, NUMB, SLC6A6, NLGN1, GRID2, IFNGR2

Annotation Cluster 11	Fibronectin	Enrichment Score: 1,2609055685526542			
Category	Term	Count	%	PValue	Genes
UP_SEQ_FEATURE	domain:Fibronectin type-III 2	4	4	0,028	SPEG, BZRAP1, IGSF9B, IFNGR2
UP_SEQ_FEATURE	domain:Fibronectin type-III 1	4	4	0,028	SPEG, BZRAP1, IGSF9B, IFNGR2
INTERPRO	IPR008957:Fibronectin, type III-like fold	4	4	0,074	SPEG, BZRAP1, IGSF9B, IFNGR2
INTERPRO	IPR003961:Fibronectin, type III	4	4	0,079	SPEG, BZRAP1, IGSF9B, IFNGR2
SMART	SM00060:FN3	4	4	0,108	SPEG, BZRAP1, IGSF9B, IFNGR2

Annotation Cluster 12	Response to stimulus	Enrichment Score: 1,1901293564170592			
Category	Term	Count	%	PValue	Genes
GOTERM_BP_ALL	GO:0032102~negative regulation of response to external stimulus	3	3	0,034	PPARA, TNFRSF1B, GRID2
GOTERM_BP_FAT	GO:0032102~negative regulation of response to external stimulus	3	3	0,035	PPARA, TNFRSF1B, GRID2
GOTERM_BP_ALL	GO:0032101~regulation of response to external stimulus	4	4	0,065	PPARA, TNFRSF1B, IL6, GRID2
GOTERM_BP_FAT	GO:0032101~regulation of response to external stimulus	4	4	0,068	PPARA, TNFRSF1B, IL6, GRID2
GOTERM_BP_ALL	GO:0048585~negative regulation of response to stimulus	3	3	0,115	PPARA, TNFRSF1B, GRID2
GOTERM_BP_FAT	GO:0048585~negative regulation of response to stimulus	3	3	0,118	PPARA, TNFRSF1B, GRID2

Annotation Cluster 13	Negative regulation of biosynthetic process	Enrichment Score: 1,1299762005337894			
Category	Term	Count	%	PValue	Genes
GOTERM_BP_ALL	GO:0031327~negative regulation of cellular biosynthetic process	8	8	0,044	PPARA, IL6, GIMAP5, SPEN, NRG1, RBM15, FOXP1, IGFBP5
GOTERM_BP_FAT	GO:0031327~negative regulation of cellular biosynthetic process	8	8	0,048	PPARA, IL6, GIMAP5, SPEN, NRG1, RBM15, FOXP1, IGFBP5
GOTERM_BP_ALL	GO:0009890~negative regulation of biosynthetic process	8	8	0,049	PPARA, IL6, GIMAP5, SPEN, NRG1, RBM15, FOXP1, IGFBP5
GOTERM_BP_FAT	GO:0009890~negative regulation of biosynthetic process	8	8	0,053	PPARA, IL6, GIMAP5, SPEN, NRG1, RBM15, FOXP1, IGFBP5
GOTERM_BP_ALL	GO:0031324~negative regulation of cellular metabolic process	9	9	0,057	PPARA, IL6, GIMAP5, MLH1, SPEN, NRG1, RBM15, FOXP1, IGFBP5

GOTERM_BP_ALL	GO:0009892~negative regulation of metabolic process	9	9	0,083	PPARA, IL6, GIMAP5, MLH1, SPEN, NRG1, RBM15, FOXP1, IGFBP5
GOTERM_BP_ALL	GO:0010558~negative regulation of macromolecule biosynthetic process	7	7	0,098	PPARA, IL6, SPEN, NRG1, RBM15, FOXP1, IGFBP5
GOTERM_BP_FAT	GO:0010558~negative regulation of macromolecule biosynthetic process	7	7	0,105	PPARA, IL6, SPEN, NRG1, RBM15, FOXP1, IGFBP5
GOTERM_BP_ALL	GO:0010605~negative regulation of macromolecule metabolic process	8	8	0,134	PPARA, IL6, MLH1, SPEN, NRG1, RBM15, FOXP1, IGFBP5
GOTERM_BP_FAT	GO:0010605~negative regulation of macromolecule metabolic process	8	8	0,143	PPARA, IL6, MLH1, SPEN, NRG1, RBM15, FOXP1, IGFBP5

**Annotation Cluster 14**	**Regulation of neuron development**	**Enrichment Score: 1,1021755439618124**			
Category	Term	Count	%	PValue	Genes
GOTERM_BP_ALL	GO:0045664~regulation of neuron differentiation	4	4	0,042	MIB1, NUMB, NLGN1, NEFL
GOTERM_BP_FAT	GO:0045664~regulation of neuron differentiation	4	4	0,044	MIB1, NUMB, NLGN1, NEFL
GOTERM_BP_ALL	GO:0050767~regulation of neurogenesis	4	4	0,072	MIB1, NUMB, NLGN1, NEFL
GOTERM_BP_FAT	GO:0050767~regulation of neurogenesis	4	4	0,075	MIB1, NUMB, NLGN1, NEFL
GOTERM_BP_ALL	GO:0051960~regulation of nervous system development	4	4	0,101	MIB1, NUMB, NLGN1, NEFL
GOTERM_BP_FAT	GO:0051960~regulation of nervous system development	4	4	0,105	MIB1, NUMB, NLGN1, NEFL
GOTERM_BP_ALL	GO:0060284~regulation of cell development	4	4	0,117	MIB1, NUMB, NLGN1, NEFL
GOTERM_BP_FAT	GO:0060284~regulation of cell development	4	4	0,121	MIB1, NUMB, NLGN1, NEFL

**Annotation Cluster 15**	**Cytoskeleton organization**	**Enrichment Score: 1,0160830243590524**			
Category	Term	Count	%	PValue	Genes
GOTERM_BP_ALL	GO:0051258~protein polymerization	3	3	0,033	CAV2, TPPP, NEFL
GOTERM_BP_FAT	GO:0051258~protein polymerization	3	3	0,034	CAV2, TPPP, NEFL
GOTERM_BP_ALL	GO:0000226~microtubule cytoskeleton organization	4	4	0,054	CAV2, TPPP, MLH1, NEFL
GOTERM_BP_FAT	GO:0000226~microtubule cytoskeleton organization	4	4	0,056	CAV2, TPPP, MLH1, NEFL
GOTERM_BP_ALL	GO:0007017~microtubule-based process	4	4	0,182	CAV2, TPPP, MLH1, NEFL

GOTERM_BP_FAT	GO:0007017~microtubule-based process	4	4	0,189	CAV2, TPPP, MLH1, NEFL
GOTERM_BP_ALL	GO:0007010~cytoskeleton organization	5	5	0,248	CAV2, ANK3, TPPP, MLH1, NEFL
GOTERM_BP_FAT	GO:0007010~cytoskeleton organization	5	5	0,257	CAV2, ANK3, TPPP, MLH1, NEFL

**Annotation Cluster 16**	**Negative regulation of metabolic process**	**Enrichment Score: 0,9939375127770929**			
Category	Term	Count	%	PValue	Genes
GOTERM_BP_ALL	GO:0031324~negative regulation of cellular metabolic process	9	9	0,057	PPARA, IL6, GIMAP5, MLH1, SPEN, NRG1, RBM15, FOXP1, IGFBP5
GOTERM_BP_ALL	GO:0051172~negative regulation of nitrogen compound metabolic process	7	7	0,081	PPARA, GIMAP5, MLH1, SPEN, NRG1, RBM15, FOXP1
GOTERM_BP_ALL	GO:0009892~negative regulation of metabolic process	9	9	0,083	PPARA, IL6, GIMAP5, MLH1, SPEN, NRG1, RBM15, FOXP1, IGFBP5
GOTERM_BP_FAT	GO:0051172~negative regulation of nitrogen compound metabolic process	7	7	0,086	PPARA, GIMAP5, MLH1, SPEN, NRG1, RBM15, FOXP1
GOTERM_BP_ALL	GO:0045934~negative regulation of nucleobase, nucleoside, nucleotide and nucleic acid metabolic process	6	6	0,177	PPARA, MLH1, SPEN, NRG1, RBM15, FOXP1
GOTERM_BP_FAT	GO:0045934~negative regulation of nucleobase, nucleoside, nucleotide and nucleic acid metabolic process	6	6	0,186	PPARA, MLH1, SPEN, NRG1, RBM15, FOXP1

**Annotation Cluster 17**	**Biological regulation**	**Enrichment Score: 0,9910627128676565**			
Category	Term	Count	%	PValue	Genes
GOTERM_BP_ALL	GO:0050789~regulation of biological process	49	51	0,084	BACH1, PPARA, CASR, E2F3, FAM3D, PLXNA2, MLH1, CALB1, ANK3, GRID2, PITPNC1, PLCB1, NRG1, SPEN, NEBL, MIB1, NCOA3, RIF1, TPPP, GAA, VOPP1, SMARCA1, CAV2, PARD3, EHF, RRM2B, GRAMD4, TNFRSF1B, MAP3K3, SPEG, NUMB, BCL9L, NEFL, ERF, IL6, GIMAP5, KLF9, PDK4, NLGN1, DGKI, FOXP1, SAFB2, DVL1, P2RY12, NEB, DIO2, DIO3, RBM15, IGFBP5
GOTERM_BP_ALL	GO:0050794~regulation of cellular process	47	48	0,096	BACH1, CAV2, PPARA, PARD3, CASR, E2F3, FAM3D, PLXNA2, MLH1, EHF, RRM2B, GRAMD4, CALB1, TNFRSF1B, MAP3K3, ANK3, SPEG, NUMB, GRID2, BCL9L, PITPNC1, NRG1, PLCB1, NEFL, ERF, IL6, GIMAP5, KLF9, PDK4, NLGN1, DGKI, SPEN, FOXP1, NEBL, SAFB2, DVL1, P2RY12, MIB1, RIF1, DIO2, NCOA3, NEB, TPPP, VOPP1, SMARCA1, RBM15, IGFBP5

GOTERM_BP_ALL	GO:0065007~biological regulation	50	52	0,132	BACH1, PPARA, CASR, E2F3, FAM3D, PLXNA2, MLH1, CALB1, ANK3, GRID2, PITPNC1, PLCB1, NRG1, NRXN3, SPEN, NEBL, MIB1, NCOA3, RIF1, TPPP, GAA, VOPP1, SMARCA1, CAV2, PARD3, EHF, RRM2B, GRAMD4, TNFRSF1B, MAP3K3, SPEG, NUMB, BCL9L, NEFL, ERF, IL6, GIMAP5, KLF9, PDK4, NLGN1, DGKI, FOXP1, SAFB2, DVL1, P2RY12, NEB, DIO2, DIO3, RBM15, IGFBP5
**Annotation Cluster 18**	**Apical part of cell**	**Enrichment Score: 0,931679541600548**			
Category	Term	Count	%	PValue	Genes
GOTERM_CC_ALL	GO:0045177~apical part of cell	4	4	0,077	CAV2, PARD3, NUMB, NRG1
GOTERM_CC_FAT	GO:0045177~apical part of cell	4	4	0,083	CAV2, PARD3, NUMB, NRG1
GOTERM_CC_ALL	GO:0016324~apical plasma membrane	3	3	0,168	CAV2, PARD3, NRG1
GOTERM_CC_FAT	GO:0016324~apical plasma membrane	3	3	0,176	CAV2, PARD3, NRG1
**Annotation Cluster 19**	**Respiratory development**	**Enrichment Score: 0,8889140975009547**			
Category	Term	Count	%	PValue	Genes
GOTERM_BP_ALL	GO:0030323~respiratory tube development	3	3	0,119	CAV2, HECA, FOXP1
GOTERM_BP_FAT	GO:0030323~respiratory tube development	3	3	0,122	CAV2, HECA, FOXP1
GOTERM_BP_ALL	GO:0035295~tube development	4	4	0,136	MIB1, CAV2, HECA, FOXP1
GOTERM_BP_FAT	GO:0035295~tube development	4	4	0,141	MIB1, CAV2, HECA, FOXP1
**Annotation Cluster 20**	**sh3 domain**	**Enrichment Score: 0,8884548223027133**			
Category	Term	Count	%	PValue	Genes
SP_PIR_KEYWORDS	sh3 domain	4	4	0,088	NEB, RUSC2, BZRAP1, NEBL
INTERPRO	IPR001452:Src homology-3 domain	4	4	0,103	NEB, RUSC2, BZRAP1, NEBL
SMART	SM00326:SH3	4	4	0,138	NEB, RUSC2, BZRAP1, NEBL
UP_SEQ_FEATURE	domain:SH3	3	3	0,223	NEB, RUSC2, NEBL
**Annotation Cluster 21**	**Regulation of transcription**	**Enrichment Score: 0,8608059285911931**			
Category	Term	Count	%	PValue	Genes
GOTERM_BP_ALL	GO:0032583~regulation of gene-specific transcription	4	4	0,043	PPARA, IL6, SMARCA1, RBM15
GOTERM_BP_FAT	GO:0032583~regulation of gene-specific transcription	4	4	0,045	PPARA, IL6, SMARCA1, RBM15

GOTERM_BP_ALL	GO:0043193~positive regulation of gene-specific transcription	3	3	0,091	IL6, SMARCA1, RBM15
GOTERM_BP_FAT	GO:0043193~positive regulation of gene-specific transcription	3	3	0,094	IL6, SMARCA1, RBM15
GOTERM_BP_ALL	GO:0045944~positive regulation of transcription from RNA polymerase II promoter	3	3	0,639	PPARA, IL6, RBM15
GOTERM_BP_FAT	GO:0045944~positive regulation of transcription from RNA polymerase II promoter	3	3	0,649	PPARA, IL6, RBM15

**Annotation Cluster 22**	**Cytoskeleton**	**Enrichment Score: 0,8385546821350619**			
Category	Term	Count	%	PValue	Genes
GOTERM_CC_ALL	GO:0044430~cytoskeletal part	10	10	0,080	PARD3, RIF1, NEB, TPPP, INCENP, NLGN1, GRID2, UPP2, NEFL, DVL1
GOTERM_CC_FAT	GO:0044430~cytoskeletal part	10	10	0,092	PARD3, RIF1, NEB, TPPP, INCENP, NLGN1, GRID2, UPP2, NEFL, DVL1
GOTERM_CC_ALL	GO:0005856~cytoskeleton	11	11	0,232	PARD3, RIF1, NEB, ANK3, TPPP, INCENP, NLGN1, GRID2, UPP2, NEFL, DVL1
GOTERM_CC_FAT	GO:0005856~cytoskeleton	11	11	0,261	PARD3, RIF1, NEB, ANK3, TPPP, INCENP, NLGN1, GRID2, UPP2, NEFL, DVL1

**Annotation Cluster 23**	**Immune system process**	**Enrichment Score: 0,8380354776005403**			
Category	Term	Count	%	PValue	Genes
GOTERM_BP_ALL	GO:0002700~regulation of production of molecular mediator of immune response	3	3	0,024	IL6, GIMAP5, FOXP1
GOTERM_BP_FAT	GO:0002700~regulation of production of molecular mediator of immune response	3	3	0,024	IL6, GIMAP5, FOXP1
GOTERM_BP_ALL	GO:0002697~regulation of immune effector process	3	3	0,117	IL6, GIMAP5, FOXP1
GOTERM_BP_FAT	GO:0002697~regulation of immune effector process	3	3	0,120	IL6, GIMAP5, FOXP1
GOTERM_BP_ALL	GO:0050776~regulation of immune response	3	3	0,379	IL6, GIMAP5, FOXP1
GOTERM_BP_ALL	GO:0002682~regulation of immune system process	4	4	0,388	IL6, GIMAP5, RBM15, FOXP1

GOTERM_BP_ALL	GO:0002684~positive regulation of immune system process	3	3	0,404	IL6, GIMAP5, FOXP1
GOTERM_BP_FAT	GO:0002684~positive regulation of immune system process	3	3	0,412	IL6, GIMAP5, FOXP1

**Annotation Cluster 24**	**Negative regulation of differentiation and development**	**Enrichment Score: 0,8232758606391365**			
Category	Term	Count	%	PValue	Genes
GOTERM_BP_ALL	GO:0045596~negative regulation of cell differentiation	4	4	0,131	MIB1, PPARA, RIF1, RBM15
GOTERM_BP_FAT	GO:0045596~negative regulation of cell differentiation	4	4	0,136	MIB1, PPARA, RIF1, RBM15
GOTERM_BP_ALL	GO:0051093~negative regulation of developmental process	4	4	0,191	MIB1, PPARA, RIF1, RBM15

**Annotation Cluster 25**	**Positive regulation of cell comunication**	**Enrichment Score: 0,8139050106865081**			
Category	Term	Count	%	PValue	Genes
GOTERM_BP_ALL	GO:0009967~positive regulation of signal transduction	5	5	0,093	CAV2, IL6, NCOA3, MAP3K3, DVL1
GOTERM_BP_FAT	GO:0009967~positive regulation of signal transduction	5	5	0,097	CAV2, IL6, NCOA3, MAP3K3, DVL1
GOTERM_BP_ALL	GO:0010647~positive regulation of cell communication	5	5	0,124	CAV2, IL6, NCOA3, MAP3K3, DVL1
GOTERM_BP_FAT	GO:0010647~positive regulation of cell communication	5	5	0,130	CAV2, IL6, NCOA3, MAP3K3, DVL1
GOTERM_BP_ALL	GO:0009966~regulation of signal transduction	6	6	0,584	CAV2, IL6, NCOA3, MAP3K3, DVL1, IGFBP5

**Annotation Cluster 26**	**Positive regulation of transcription and biosynthesis**	**Enrichment Score: 0,7876562593073575**			
Category	Term	Count	%	PValue	Genes
GOTERM_BP_ALL	GO:0045893~positive regulation of transcription, DNA-dependent	7	7	0,059	PPARA, E2F3, IL6, NCOA3, EHF, SMARCA1, RBM15
GOTERM_BP_ALL	GO:0051254~positive regulation of RNA metabolic process	7	7	0,061	PPARA, E2F3, IL6, NCOA3, EHF, SMARCA1, RBM15
GOTERM_BP_FAT	GO:0045893~positive regulation of transcription, DNA-dependent	7	7	0,063	PPARA, E2F3, IL6, NCOA3, EHF, SMARCA1, RBM15

GOTERM_BP_FAT	GO:0051254~positive regulation of RNA metabolic process	7	7	0,065	PPARA, E2F3, IL6, NCOA3, EHF, SMARCA1, RBM15
GOTERM_BP_ALL	GO:0045941~positive regulation of transcription	7	7	0,109	PPARA, E2F3, IL6, NCOA3, EHF, SMARCA1, RBM15
GOTERM_BP_FAT	GO:0045941~positive regulation of transcription	7	7	0,116	PPARA, E2F3, IL6, NCOA3, EHF, SMARCA1, RBM15
GOTERM_BP_ALL	GO:0010628~positive regulation of gene expression	7	7	0,121	PPARA, E2F3, IL6, NCOA3, EHF, SMARCA1, RBM15
GOTERM_BP_FAT	GO:0010628~positive regulation of gene expression	7	7	0,129	PPARA, E2F3, IL6, NCOA3, EHF, SMARCA1, RBM15
GOTERM_BP_ALL	GO:0045935~positive regulation of nucleobase, nucleoside, nucleotide and nucleic acid metabolic process	7	7	0,154	PPARA, E2F3, IL6, NCOA3, EHF, SMARCA1, RBM15
GOTERM_BP_FAT	GO:0045935~positive regulation of nucleobase, nucleoside, nucleotide and nucleic acid metabolic process	7	7	0,163	PPARA, E2F3, IL6, NCOA3, EHF, SMARCA1, RBM15
GOTERM_BP_ALL	GO:0051173~positive regulation of nitrogen compound metabolic process	7	7	0,171	PPARA, E2F3, IL6, NCOA3, EHF, SMARCA1, RBM15
GOTERM_BP_ALL	GO:0010557~positive regulation of macromolecule biosynthetic process	7	7	0,179	PPARA, E2F3, IL6, NCOA3, EHF, SMARCA1, RBM15
GOTERM_BP_FAT	GO:0051173~positive regulation of nitrogen compound metabolic process	7	7	0,181	PPARA, E2F3, IL6, NCOA3, EHF, SMARCA1, RBM15
GOTERM_BP_FAT	GO:0010557~positive regulation of macromolecule biosynthetic process	7	7	0,190	PPARA, E2F3, IL6, NCOA3, EHF, SMARCA1, RBM15
GOTERM_BP_ALL	GO:0031328~positive regulation of cellular biosynthetic process	7	7	0,207	PPARA, E2F3, IL6, NCOA3, EHF, SMARCA1, RBM15
GOTERM_BP_ALL	GO:0009891~positive regulation of biosynthetic process	7	7	0,216	PPARA, E2F3, IL6, NCOA3, EHF, SMARCA1, RBM15
GOTERM_BP_FAT	GO:0031328~positive regulation of cellular biosynthetic process	7	7	0,218	PPARA, E2F3, IL6, NCOA3, EHF, SMARCA1, RBM15
GOTERM_BP_FAT	GO:0009891~positive regulation of biosynthetic process	7	7	0,228	PPARA, E2F3, IL6, NCOA3, EHF, SMARCA1, RBM15
GOTERM_BP_ALL	GO:0010604~positive regulation of macromolecule metabolic process	7	7	0,380	PPARA, E2F3, IL6, NCOA3, EHF, SMARCA1, RBM15
GOTERM_BP_FAT	GO:0010604~positive regulation of macromolecule metabolic process	7	7	0,396	PPARA, E2F3, IL6, NCOA3, EHF, SMARCA1, RBM15

GOTERM_BP_ALL	GO:0031325~positive regulation of cellular metabolic process	7	7	0,404	PPARA, E2F3, IL6, NCOA3, EHF, SMARCA1, RBM15
GOTERM_BP_ALL	GO:0009893~positive regulation of metabolic process	7	7	0,447	PPARA, E2F3, IL6, NCOA3, EHF, SMARCA1, RBM15

**Annotation Cluster 27**	**Positive regulation of cell differentiation and development vs. negative regulation of apoptosis**	**Enrichment Score: 0,7612494687464665**			
Category	Term	Count	%	PValue	Genes
GOTERM_BP_ALL	GO:0045597~positive regulation of cell differentiation	5	5	0,044	IL6, GIMAP5, NUMB, NRG1, NEFL
GOTERM_BP_FAT	GO:0045597~positive regulation of cell differentiation	5	5	0,047	IL6, GIMAP5, NUMB, NRG1, NEFL
GOTERM_BP_ALL	GO:0051094~positive regulation of developmental process	5	5	0,078	IL6, GIMAP5, NUMB, NRG1, NEFL
GOTERM_BP_FAT	GO:0051094~positive regulation of developmental process	5	5	0,082	IL6, GIMAP5, NUMB, NRG1, NEFL
GOTERM_BP_ALL	GO:0043066~negative regulation of apoptosis	4	4	0,339	IL6, GIMAP5, NRG1, NEFL
GOTERM_BP_ALL	GO:0043069~negative regulation of programmed cell death	4	4	0,347	IL6, GIMAP5, NRG1, NEFL
GOTERM_BP_ALL	GO:0060548~negative regulation of cell death	4	4	0,348	IL6, GIMAP5, NRG1, NEFL
GOTERM_BP_FAT	GO:0043066~negative regulation of apoptosis	4	4	0,349	IL6, GIMAP5, NRG1, NEFL
GOTERM_BP_FAT	GO:0043069~negative regulation of programmed cell death	4	4	0,357	IL6, GIMAP5, NRG1, NEFL
GOTERM_BP_FAT	GO:0060548~negative regulation of cell death	4	4	0,359	IL6, GIMAP5, NRG1, NEFL

**Annotation Cluster 28**	**Synaptic transmission**	**Enrichment Score: 0,7563165514224575**			
Category	Term	Count	%	PValue	Genes
GOTERM_BP_ALL	GO:0007268~synaptic transmission	5	5	0,095	CAV2, NRXN3, NLGN1, GRID2, DVL1
GOTERM_BP_FAT	GO:0007268~synaptic transmission	5	5	0,100	CAV2, NRXN3, NLGN1, GRID2, DVL1
GOTERM_BP_ALL	GO:0019226~transmission of nerve impulse	5	5	0,146	CAV2, NRXN3, NLGN1, GRID2, DVL1

GOTERM_BP_FAT	GO:0019226~transmission of nerve impulse	5	5	0,153	CAV2, NRXN3, NLGN1, GRID2, DVL1
GOTERM_BP_ALL	GO:0007267~cell-cell signaling	6	6	0,269	CAV2, IL6, NRXN3, NLGN1, GRID2, DVL1
GOTERM_BP_FAT	GO:0007267~cell-cell signaling	6	6	0,281	CAV2, IL6, NRXN3, NLGN1, GRID2, DVL1
GOTERM_BP_ALL	GO:0007154~cell communication	7	7	0,315	CAV2, IL6, NRXN3, NLGN1, GRID2, NRG1, DVL1

**Annotation Cluster 29**	**Organelle organization**	**Enrichment Score: 0,7532512191024592**			
Category	Term	Count	%	PValue	Genes
GOTERM_BP_ALL	GO:0007005~mitochondrion organization	4	4	0,046	CAV2, LETM1, GIMAP5, RRM2B
GOTERM_BP_FAT	GO:0007005~mitochondrion organization	4	4	0,048	CAV2, LETM1, GIMAP5, RRM2B
GOTERM_BP_ALL	GO:0016044~membrane organization	3	3	0,654	CAV2, LETM1, GIMAP5
GOTERM_BP_FAT	GO:0016044~membrane organization	3	3	0,663	CAV2, LETM1, GIMAP5

**Annotation Cluster 30**	**Cellular fractions**	**Enrichment Score: 0,6904723718104767**			
Category	Term	Count	%	PValue	Genes
GOTERM_CC_ALL	GO:0005624~membrane fraction	8	8	0,160	CAV2, TNFRSF1B, ANK3, SLC24A1, GRID2, PLCB1, ACSL6, DVL1
GOTERM_CC_FAT	GO:0005624~membrane fraction	8	8	0,178	CAV2, TNFRSF1B, ANK3, SLC24A1, GRID2, PLCB1, ACSL6, DVL1
GOTERM_CC_ALL	GO:0005626~insoluble fraction	8	8	0,182	CAV2, TNFRSF1B, ANK3, SLC24A1, GRID2, PLCB1, ACSL6, DVL1
GOTERM_CC_FAT	GO:0005626~insoluble fraction	8	8	0,202	CAV2, TNFRSF1B, ANK3, SLC24A1, GRID2, PLCB1, ACSL6, DVL1
GOTERM_CC_ALL	GO:0000267~cell fraction	9	9	0,249	CAV2, TNFRSF1B, ANK3, TPPP, SLC24A1, GRID2, PLCB1, ACSL6, DVL1
GOTERM_CC_FAT	GO:0000267~cell fraction	9	9	0,276	CAV2, TNFRSF1B, ANK3, TPPP, SLC24A1, GRID2, PLCB1, ACSL6, DVL1

**Annotation Cluster 31**	**Immunoglobulin**	**Enrichment Score: 0,6584280139890469**			
Category	Term	Count	%	PValue	Genes
INTERPRO	IPR003598:Immunoglobulin subtype 2	4	4	0,095	ADAMTSL1, SPEG, IGSF9B, NRG1
SMART	SM00408:IGc2	4	4	0,127	ADAMTSL1, SPEG, IGSF9B, NRG1
SP_PIR_KEYWORDS	Immunoglobulin domain	5	5	0,208	ADAMTSL1, SPEG, IGSF9B, NRG1, C10ORF72
INTERPRO	IPR003599:Immunoglobulin subtype	4	4	0,253	ADAMTSL1, SPEG, IGSF9B, C10ORF72
INTERPRO	IPR007110:Immunoglobulin-like	5	5	0,273	ADAMTSL1, SPEG, IGSF9B, NRG1, C10ORF72
INTERPRO	IPR013151:Immunoglobulin	3	3	0,289	ADAMTSL1, IGSF9B, NRG1
SMART	SM00409:IG	4	4	0,323	ADAMTSL1, SPEG, IGSF9B, C10ORF72

INTERPRO	IPR013783:Immunoglobulin-like fold	5	5	0,335	ADAMTSL1, SPEG, IGSF9B, NRG1, C10ORF72

**Annotation Cluster 32**	**Carboxilic acid metabolism**	**Enrichment Score: 0,6470577930577035**			
Category	Term	Count	%	PValue	Genes
GOTERM_BP_ALL	GO:0043436~oxoacid metabolic process	6	6	0,221	PPARA, SLC6A6, FASN, SUCLA2, ADC, ACSL6
GOTERM_BP_ALL	GO:0019752~carboxylic acid metabolic process	6	6	0,221	PPARA, SLC6A6, FASN, SUCLA2, ADC, ACSL6
GOTERM_BP_ALL	GO:0006082~organic acid metabolic process	6	6	0,226	PPARA, SLC6A6, FASN, SUCLA2, ADC, ACSL6
GOTERM_BP_ALL	GO:0042180~cellular ketone metabolic process	6	6	0,233	PPARA, SLC6A6, FASN, SUCLA2, ADC, ACSL6

**Annotation Cluster 33**	**Cell adhesion**	**Enrichment Score: 0,6315895208039075**			
Category	Term	Count	%	PValue	Genes
GOTERM_CC_ALL	GO:0005912~adherens junction	3	3	0,212	CAV2, PARD3, LIMS1
GOTERM_CC_FAT	GO:0005912~adherens junction	3	3	0,222	CAV2, PARD3, LIMS1
GOTERM_CC_ALL	GO:0070161~anchoring junction	3	3	0,246	CAV2, PARD3, LIMS1
GOTERM_CC_FAT	GO:0070161~anchoring junction	3	3	0,258	CAV2, PARD3, LIMS1

**Annotation Cluster 34**	**Bone development**	**Enrichment Score: 0,62532914591469**			
Category	Term	Count	%	PValue	Genes
GOTERM_BP_ALL	GO:0001503~ossification	3	3	0,144	CASR, COL13A1, IGFBP5
GOTERM_BP_FAT	GO:0001503~ossification	3	3	0,148	CASR, COL13A1, IGFBP5
GOTERM_BP_ALL	GO:0060348~bone development	3	3	0,160	CASR, COL13A1, IGFBP5
GOTERM_BP_FAT	GO:0060348~bone development	3	3	0,165	CASR, COL13A1, IGFBP5
GOTERM_BP_ALL	GO:0001501~skeletal system development	3	3	0,556	CASR, COL13A1, IGFBP5
GOTERM_BP_FAT	GO:0001501~skeletal system development	3	3	0,566	CASR, COL13A1, IGFBP5

**Annotation Cluster 35**	**Calcium ion binding**	**Enrichment Score: 0,6108546435406391**			
Category	Term	Count	%	PValue	Genes
SP_PIR_KEYWORDS	calcium	7	7	0,212	LETM1, PPP2R3A, NRXN3, SLC24A1, FAM20C, PLCB1, CALB1
GOTERM_MF_ALL	GO:0005509~calcium ion binding	8	8	0,248	LETM1, PPP2R3A, NRXN3, CD69, SLC24A1, FAM20C, PLCB1, CALB1
GOTERM_MF_FAT	GO:0005509~calcium ion binding	8	8	0,280	LETM1, PPP2R3A, NRXN3, CD69, SLC24A1, FAM20C, PLCB1, CALB1

Annotation Cluster 36	Extracellular region	Enrichment Score: 0,6046618253289845			
Category	Term	Count	%	PValue	Genes
SP_PIR_KEYWORDS	Secreted	13	13	0,135	IL6, ADAMTSL1, FAM3D, MMEL1, FAM20C, OLFML2A, ST3GAL1, TNFRSF1B, RSPO1, ITIH5, NRG1, ADAMTS5, IGFBP5
GOTERM_CC_ALL	GO:0005576~extracellular region	14	14	0,317	IL6, ADAMTSL1, FAM3D, MMEL1, COL13A1, FAM20C, OLFML2A, ST3GAL1, TNFRSF1B, RSPO1, ITIH5, NRG1, ADAMTS5, IGFBP5
GOTERM_CC_FAT	GO:0005576~extracellular region	14	14	0,359	IL6, ADAMTSL1, FAM3D, MMEL1, COL13A1, FAM20C, OLFML2A, ST3GAL1, TNFRSF1B, RSPO1, ITIH5, NRG1, ADAMTS5, IGFBP5

Annotation Cluster 37	Mitochondrion	Enrichment Score: 0,5930286045962284			
Category	Term	Count	%	PValue	Genes
SP_PIR_KEYWORDS	mitochondrion	7	7	0,236	LETM1, GIMAP5, PDK4, BZRAP1, SUCLA2, GRAMD4, ACSL6
GOTERM_CC_ALL	GO:0005739~mitochondrion	9	9	0,252	CAV2, LETM1, GIMAP5, PDK4, BZRAP1, SUCLA2, GRAMD4, ADC, ACSL6
GOTERM_CC_FAT	GO:0005739~mitochondrion	9	9	0,280	CAV2, LETM1, GIMAP5, PDK4, BZRAP1, SUCLA2, GRAMD4, ADC, ACSL6

Annotation Cluster 38	Response to stimulus	Enrichment Score: 0,5857736396045381			
Category	Term	Count	%	PValue	Genes
GOTERM_BP_ALL	GO:0009611~response to wounding	6	6	0,195	P2RY12, PPARA, TNFRSF1B, IL6, NRG1, NEFL
GOTERM_BP_FAT	GO:0009611~response to wounding	6	6	0,204	P2RY12, PPARA, TNFRSF1B, IL6, NRG1, NEFL
GOTERM_BP_ALL	GO:0009605~response to external stimulus	7	7	0,440	P2RY12, PPARA, TNFRSF1B, IL6, GRID2, NRG1, NEFL

Annotation Cluster 39	Organelle organization	Enrichment Score: 0,5804402483993681			
Category	Term	Count	%	PValue	Genes
GOTERM_BP_ALL	GO:0051493~regulation of cytoskeleton organization	3	3	0,187	CAV2, NEB, NEBL
GOTERM_BP_FAT	GO:0051493~regulation of cytoskeleton organization	3	3	0,192	CAV2, NEB, NEBL
GOTERM_BP_ALL	GO:0033043~regulation of organelle organization	3	3	0,360	CAV2, NEB, NEBL
GOTERM_BP_FAT	GO:0033043~regulation of organelle organization	3	3	0,368	CAV2, NEB, NEBL

Annotation Cluster 40	Coenzyme and cofactor metabolic process	Enrichment Score: 0,5723309153527573			
Category	Term	Count	%	PValue	Genes
GOTERM_BP_ALL	GO:0006732~coenzyme metabolic process	3	3	0,223	TPK1, SUCLA2, ACSL6
GOTERM_BP_FAT	GO:0006732~coenzyme metabolic process	3	3	0,229	TPK1, SUCLA2, ACSL6
GOTERM_BP_ALL	GO:0051186~cofactor metabolic process	3	3	0,313	TPK1, SUCLA2, ACSL6
GOTERM_BP_FAT	GO:0051186~cofactor metabolic process	3	3	0,321	TPK1, SUCLA2, ACSL6

Annotation Cluster 41	Golgi apparatus	Enrichment Score: 0,570769606159717			
Category	Term	Count	%	PValue	Genes
GOTERM_CC_ALL	GO:0044431~Golgi apparatus part	4	4	0,222	ST3GAL1, CAV2, ST3GAL5, GOLIM4
GOTERM_CC_FAT	GO:0044431~Golgi apparatus part	4	4	0,236	ST3GAL1, CAV2, ST3GAL5, GOLIM4
GOTERM_CC_ALL	GO:0000139~Golgi membrane	3	3	0,275	ST3GAL1, CAV2, ST3GAL5
GOTERM_CC_FAT	GO:0000139~Golgi membrane	3	3	0,287	ST3GAL1, CAV2, ST3GAL5
SP_PIR_KEYWORDS	golgi apparatus	5	5	0,338	ST3GAL1, CAV2, ST3GAL5, GOLIM4, LRBA

Annotation Cluster 42	Neurological system process	Enrichment Score: 0,5704839487400494			
Category	Term	Count	%	PValue	Genes
GOTERM_BP_ALL	GO:0003008~system process	12	12	0,254	CAV2, PPARA, NRXN3, SLC24A1, NLGN1, GAA, GRID2, RRM2B, PLCB1, NEFL, CALB1, DVL1
GOTERM_BP_ALL	GO:0050877~neurological system process	10	10	0,268	CAV2, NRXN3, SLC24A1, NLGN1, GAA, GRID2, PLCB1, NEFL, CALB1, DVL1
GOTERM_BP_FAT	GO:0050877~neurological system process	10	10	0,286	CAV2, NRXN3, SLC24A1, NLGN1, GAA, GRID2, PLCB1, NEFL, CALB1, DVL1

Annotation Cluster 43	Regulation of transcription	Enrichment Score: 0,5608912536470765			
Category	Term	Count	%	PValue	Genes
GOTERM_MF_ALL	GO:0030528~transcription regulator activity	12	12	0,214	BACH1, ERF, PPARA, E2F3, KLF9, NCOA3, EHF, SPEN, NRG1, SMARCA1, GRAMD4, FOXP1
GOTERM_BP_ALL	GO:0051252~regulation of RNA metabolic process	14	14	0,250	BACH1, ERF, PPARA, IL6, E2F3, KLF9, EHF, SPEN, GRAMD4, FOXP1, TNFRSF1B, NCOA3, SMARCA1, RBM15
GOTERM_MF_FAT	GO:0030528~transcription regulator activity	12	12	0,253	BACH1, ERF, PPARA, E2F3, KLF9, NCOA3, EHF, SPEN, NRG1, SMARCA1, GRAMD4, FOXP1
GOTERM_BP_FAT	GO:0051252~regulation of RNA metabolic process	14	14	0,270	BACH1, ERF, PPARA, IL6, E2F3, KLF9, EHF, SPEN, GRAMD4, FOXP1, TNFRSF1B, NCOA3, SMARCA1, RBM15
GOTERM_BP_ALL	GO:0006355~regulation of transcription, DNA-dependent	13	13	0,332	BACH1, PPARA, ERF, IL6, E2F3, KLF9, EHF, SPEN, GRAMD4, FOXP1, NCOA3, SMARCA1, RBM15

GOTERM_BP_FAT	GO:0006355~regulation of transcription, DNA-dependent	13	13	0,355	BACH1, PPARA, ERF, IL6, E2F3, KLF9, EHF, SPEN, GRAMD4, FOXP1, NCOA3, SMARCA1, RBM15

**Annotation Cluster 44**	**Response to virus and biotic stimulus**	**Enrichment Score: 0,556153066779096**			
Category	Term	Count	%	PValue	Genes
GOTERM_BP_ALL	GO:0009615~response to virus	3	3	0,132	IL6, DNAJC3, IFNGR2
GOTERM_BP_FAT	GO:0009615~response to virus	3	3	0,136	IL6, DNAJC3, IFNGR2
GOTERM_BP_ALL	GO:0051707~response to other organism	3	3	0,505	IL6, DNAJC3, IFNGR2
GOTERM_BP_ALL	GO:0009607~response to biotic stimulus	3	3	0,658	IL6, DNAJC3, IFNGR2

**Annotation Cluster 45**	**Membrane**	**Enrichment Score: 0,5561367601946631**			
Category	Term	Count	%	PValue	Genes
SP_PIR_KEYWORDS	transmembrane	10	11	0,052	ST3GAL1, BLB1, LETM1, ST3GAL5, DIO2, DIO3, CD69, SLC6A6, SLC24A1, NRG1
UP_SEQ_FEATURE	transmembrane region	6	6	0,110	ST3GAL1, LETM1, DIO2, DIO3, SLC24A1, NRG1
SP_PIR_KEYWORDS	membrane	9	9	0,179	ST3GAL1, LETM1, ST3GAL5, DIO2, DIO3, CD69, SLC6A6, SLC24A1, NRG1
GOTERM_CC_ALL	GO:0016020~membrane	19	20	0,334	CASR, BLB1, IL6, PLXNA2, NLGN1, OLFML2A, CALB1, ST3GAL1, LETM1, TNFRSF1B, DIO2, ST3GAL5, CD69, DIO3, NUMB, SLC6A6, SLC24A1, GRID2, NRG1
GOTERM_CC_ALL	GO:0044425~membrane part	14	15	0,361	BLB1, IL6, CASR, ST3GAL1, LETM1, TNFRSF1B, ST3GAL5, DIO2, CD69, DIO3, NUMB, SLC6A6, SLC24A1, NRG1
GOTERM_CC_ALL	GO:0016021~integral to membrane	11	12	0,449	ST3GAL1, CASR, BLB1, LETM1, ST3GAL5, DIO2, DIO3, CD69, SLC6A6, SLC24A1, NRG1
GOTERM_CC_ALL	GO:0031224~intrinsic to membrane	11	12	0,500	ST3GAL1, CASR, BLB1, LETM1, ST3GAL5, DIO2, DIO3, CD69, SLC6A6, SLC24A1, NRG1
GOTERM_CC_FAT	GO:0016021~integral to membrane	11	12	0,569	ST3GAL1, CASR, BLB1, LETM1, ST3GAL5, DIO2, DIO3, CD69, SLC6A6, SLC24A1, NRG1
GOTERM_CC_FAT	GO:0031224~intrinsic to membrane	11	12	0,626	ST3GAL1, CASR, BLB1, LETM1, ST3GAL5, DIO2, DIO3, CD69, SLC6A6, SLC24A1, NRG1

**Annotation Cluster 46**	**Circulatory system process**	**Enrichment Score: 0,5262747262308948**			
Category	Term	Count	%	PValue	Genes
GOTERM_BP_ALL	GO:0008015~blood circulation	3	3	0,294	CAV2, PPARA, GAA
GOTERM_BP_ALL	GO:0003013~circulatory system process	3	3	0,294	CAV2, PPARA, GAA
GOTERM_BP_FAT	GO:0008015~blood circulation	3	3	0,301	CAV2, PPARA, GAA

GOTERM_BP_FAT	GO:0003013~circulatory system process	3	3	0,301	CAV2, PPARA, GAA

**Annotation Cluster 47**	**Chromosome**	**Enrichment Score: 0,5119166765940161**			
Category	Term	Count	%	PValue	Genes
GOTERM_CC_ALL	GO:0000793~condensed chromosome	3	3	0,160	INCENP, MLH1, AHCTF1
GOTERM_CC_FAT	GO:0000793~condensed chromosome	3	3	0,168	INCENP, MLH1, AHCTF1
GOTERM_CC_ALL	GO:0044427~chromosomal part	4	4	0,360	RIF1, INCENP, MLH1, AHCTF1
GOTERM_CC_FAT	GO:0044427~chromosomal part	4	4	0,379	RIF1, INCENP, MLH1, AHCTF1
GOTERM_CC_ALL	GO:0005694~chromosome	4	4	0,470	RIF1, INCENP, MLH1, AHCTF1
GOTERM_CC_FAT	GO:0005694~chromosome	4	4	0,492	RIF1, INCENP, MLH1, AHCTF1

**Annotation Cluster 48**	**Regulation of translation**	**Enrichment Score: 0,5049349565737768**			
Category	Term	Count	%	PValue	Genes
GOTERM_BP_ALL	GO:0010608~posttranscriptional regulation of gene expression	4	4	0,124	TNFRSF1B, IL6, DIO2, IGFBP5
GOTERM_BP_FAT	GO:0010608~posttranscriptional regulation of gene expression	4	4	0,129	TNFRSF1B, IL6, DIO2, IGFBP5
GOTERM_BP_ALL	GO:0006417~regulation of translation	3	3	0,189	IL6, DIO2, IGFBP5
GOTERM_BP_FAT	GO:0006417~regulation of translation	3	3	0,194	IL6, DIO2, IGFBP5
GOTERM_BP_ALL	GO:0032268~regulation of cellular protein metabolic process	3	3	0,767	IL6, DIO2, IGFBP5
GOTERM_BP_FAT	GO:0032268~regulation of cellular protein metabolic process	3	3	0,776	IL6, DIO2, IGFBP5
GOTERM_BP_ALL	GO:0051246~regulation of protein metabolic process	3	3	0,832	IL6, DIO2, IGFBP5

**Annotation Cluster 49**	**Membrane**	**Enrichment Score: 0,5022738720592737**			
Category	Term	Count	%	PValue	Genes
UP_SEQ_FEATURE	topological domain:Cytoplasmic	25	26	0,044	CAV2, CASR, PARD3, PLXNA2, ST3GAL1, TNFRSF1B, ST3GAL5, CD69, SLC24A1, GRID2, NRG1, IFNGR2, C10ORF72, ACSL6, GIMAP5, MMEL1, NRXN3, COL13A1, GOLIM4, NLGN1, IGSF9B, P2RY12, DIO3, SLC6A6, VOPP1
UP_SEQ_FEATURE	transmembrane region	29	30	0,250	CAV2, PARD3, CASR, PLXNA2, LRBA, GRAMD4, ST3GAL1, TNFRSF1B, ST3GAL5, CD69, SLC24A1, GRID2, NRG1, IFNGR2, ACSL6, C10ORF72, GIMAP5, MMEL1, NRXN3, COL13A1, NLGN1, GOLIM4, IGSF9B, P2RY12, LETM1, DIO2, DIO3, SLC6A6, VOPP1

SP_PIR_KEYWORDS	transmembrane	29	30	0,262	CAV2, PARD3, CASR, PLXNA2, LRBA, GRAMD4, ST3GAL1, TNFRSF1B, ST3GAL5, CD69, SLC24A1, GRID2, NRG1, IFNGR2, ACSL6, C10ORF72, GIMAP5, MMEL1, NRXN3, COL13A1, NLGN1, GOLIM4, IGSF9B, P2RY12, LETM1, DIO2, DIO3, SLC6A6, VOPP1
GOTERM_CC_ALL	GO:0016021~integral to membrane	32	33	0,389	CAV2, PARD3, CASR, PLXNA2, LRBA, AHCTF1, GRAMD4, ST3GAL1, TNFRSF1B, ST3GAL5, CD69, NUMB, SLC24A1, GRID2, NRG1, IFNGR2, ACSL6, C10ORF72, IL6, GIMAP5, MMEL1, NRXN3, COL13A1, NLGN1, GOLIM4, IGSF9B, P2RY12, LETM1, DIO2, DIO3, SLC6A6, VOPP1
GOTERM_CC_FAT	GO:0016021~integral to membrane	32	33	0,474	CAV2, PARD3, CASR, PLXNA2, LRBA, AHCTF1, GRAMD4, ST3GAL1, TNFRSF1B, ST3GAL5, CD69, NUMB, SLC24A1, GRID2, NRG1, IFNGR2, ACSL6, C10ORF72, IL6, GIMAP5, MMEL1, NRXN3, COL13A1, NLGN1, GOLIM4, IGSF9B, P2RY12, LETM1, DIO2, DIO3, SLC6A6, VOPP1
GOTERM_CC_ALL	GO:0031224~intrinsic to membrane	32	33	0,481	CAV2, PARD3, CASR, PLXNA2, LRBA, AHCTF1, GRAMD4, ST3GAL1, TNFRSF1B, ST3GAL5, CD69, NUMB, SLC24A1, GRID2, NRG1, IFNGR2, ACSL6, C10ORF72, IL6, GIMAP5, MMEL1, NRXN3, COL13A1, NLGN1, GOLIM4, IGSF9B, P2RY12, LETM1, DIO2, DIO3, SLC6A6, VOPP1
GOTERM_CC_FAT	GO:0031224~intrinsic to membrane	32	33	0,575	CAV2, PARD3, CASR, PLXNA2, LRBA, AHCTF1, GRAMD4, ST3GAL1, TNFRSF1B, ST3GAL5, CD69, NUMB, SLC24A1, GRID2, NRG1, IFNGR2, ACSL6, C10ORF72, IL6, GIMAP5, MMEL1, NRXN3, COL13A1, NLGN1, GOLIM4, IGSF9B, P2RY12, LETM1, DIO2, DIO3, SLC6A6, VOPP1
GOTERM_CC_ALL	GO:0044425~membrane part	36	37	0,657	CAV2, PARD3, LIMS1, CASR, PLXNA2, LRBA, AHCTF1, MITD1, GRAMD4, ST3GAL1, TNFRSF1B, ST3GAL5, ANK3, CD69, NUMB, SLC24A1, GRID2, NRG1, IFNGR2, ACSL6, C10ORF72, IL6, GIMAP5, MMEL1, NRXN3, COL13A1, LRRC1, NLGN1, GOLIM4, IGSF9B, P2RY12, LETM1, DIO2, DIO3, SLC6A6, VOPP1

**Annotation Cluster 50**	**Regulation of transcription**	**Enrichment Score: 0,49962107107410886**			
Category	Term	Count	%	PValue	Genes
GOTERM_BP_ALL	GO:0045934~negative regulation of nucleobase, nucleoside, nucleotide and nucleic acid metabolic process	6	6	0,177	PPARA, MLH1, SPEN, NRG1, RBM15, FOXP1

GOTERM_BP_FAT	GO:0045934~negative regulation of nucleobase, nucleoside, nucleotide and nucleic acid metabolic process	6	6	0,186	PPARA, MLH1, SPEN, NRG1, RBM15, FOXP1
GOTERM_BP_ALL	GO:0016481~negative regulation of transcription	5	5	0,277	PPARA, SPEN, NRG1, RBM15, FOXP1
GOTERM_BP_FAT	GO:0016481~negative regulation of transcription	5	5	0,288	PPARA, SPEN, NRG1, RBM15, FOXP1
GOTERM_BP_ALL	GO:0010629~negative regulation of gene expression	5	5	0,336	PPARA, SPEN, NRG1, RBM15, FOXP1
GOTERM_BP_ALL	GO:0045892~negative regulation of transcription, DNA-dependent	4	4	0,342	PPARA, SPEN, RBM15, FOXP1
GOTERM_BP_FAT	GO:0010629~negative regulation of gene expression	5	5	0,348	PPARA, SPEN, NRG1, RBM15, FOXP1
GOTERM_BP_ALL	GO:0051253~negative regulation of RNA metabolic process	4	4	0,352	PPARA, SPEN, RBM15, FOXP1
GOTERM_BP_FAT	GO:0045892~negative regulation of transcription, DNA-dependent	4	4	0,352	PPARA, SPEN, RBM15, FOXP1
GOTERM_BP_FAT	GO:0051253~negative regulation of RNA metabolic process	4	4	0,362	PPARA, SPEN, RBM15, FOXP1
GOTERM_BP_ALL	GO:0000122~negative regulation of transcription from RNA polymerase II promoter	3	3	0,459	PPARA, RBM15, FOXP1
GOTERM_BP_FAT	GO:0000122~negative regulation of transcription from RNA polymerase II promoter	3	3	0,469	PPARA, RBM15, FOXP1

**Annotation Cluster 51**	**Regulation of molecular function**	**Enrichment Score: 0,4916922889552043**			
Category	Term	Count	%	PValue	Genes
GOTERM_BP_ALL	GO:0044093~positive regulation of molecular function	6	6	0,254	CASR, PARD3, IL6, NCOA3, DGKI, NRG1
GOTERM_BP_FAT	GO:0044093~positive regulation of molecular function	6	6	0,265	CASR, PARD3, IL6, NCOA3, DGKI, NRG1
GOTERM_BP_ALL	GO:0065009~regulation of molecular function	7	7	0,497	CASR, PARD3, IL6, NCOA3, DGKI, NRG1, DVL1

**Annotation Cluster 52**	**Cell cycle**	**Enrichment Score: 0,478239476924004**			
Category	Term	Count	%	PValue	Genes

SP_PIR_KEYWORDS	cell cycle	6	6	0,081	E2F3, PARD3, RIF1, INCENP, MLH1, AHCTF1
GOTERM_BP_ALL	GO:0007049~cell cycle	5	5	0,667	E2F3, PARD3, RIF1, INCENP, MLH1
GOTERM_BP_FAT	GO:0007049~cell cycle	5	5	0,681	E2F3, PARD3, RIF1, INCENP, MLH1

**Annotation Cluster 53**	**Organelle**	**Enrichment Score: 0,4772544854767905**			
Category	Term	Count	%	PValue	Genes
GOTERM_CC_ALL	GO:0045111~intermediate filament cytoskeleton	3	3	0,074	LOC431317, LOC395772, NEFL
GOTERM_CC_ALL	GO:0005882~intermediate filament	3	3	0,074	LOC431317, LOC395772, NEFL
GOTERM_CC_FAT	GO:0005882~intermediate filament	3	3	0,083	LOC431317, LOC395772, NEFL
GOTERM_CC_FAT	GO:0045111~intermediate filament cytoskeleton	3	3	0,083	LOC431317, LOC395772, NEFL
GOTERM_CC_ALL	GO:0044422~organelle part	10	11	0,191	ST3GAL1, LETM1, E2F3, ST3GAL5, INCENP, LOC431317, SMARCA1, LOC395772, CALB1, NEFL
GOTERM_CC_ALL	GO:0044430~cytoskeletal part	4	4	0,246	INCENP, LOC431317, LOC395772, NEFL
GOTERM_CC_FAT	GO:0044430~cytoskeletal part	4	4	0,283	INCENP, LOC431317, LOC395772, NEFL
GOTERM_CC_ALL	GO:0044446~intracellular organelle part	9	9	0,313	ST3GAL1, LETM1, E2F3, ST3GAL5, INCENP, LOC431317, SMARCA1, LOC395772, NEFL
GOTERM_CC_ALL	GO:0005856~cytoskeleton	4	4	0,446	INCENP, LOC431317, LOC395772, NEFL
GOTERM_CC_ALL	GO:0043234~protein complex	7	7	0,469	BLB1, E2F3, INCENP, LOC431317, SMARCA1, LOC395772, NEFL
GOTERM_CC_FAT	GO:0005856~cytoskeleton	4	4	0,499	INCENP, LOC431317, LOC395772, NEFL
GOTERM_MF_FAT	GO:0005198~structural molecule activity	3	3	0,657	LOC431317, LOC395772, NEFL
GOTERM_MF_ALL	GO:0005198~structural molecule activity	3	3	0,669	LOC431317, LOC395772, NEFL
GOTERM_CC_ALL	GO:0032991~macromolecular complex	7	7	0,691	BLB1, E2F3, INCENP, LOC431317, SMARCA1, LOC395772, NEFL
GOTERM_CC_ALL	GO:0043228~non-membrane-bounded organelle	4	4	0,824	INCENP, LOC431317, LOC395772, NEFL
GOTERM_CC_ALL	GO:0043232~intracellular non-membrane-bounded organelle	4	4	0,824	INCENP, LOC431317, LOC395772, NEFL
GOTERM_CC_FAT	GO:0043232~intracellular non-membrane-bounded organelle	4	4	0,870	INCENP, LOC431317, LOC395772, NEFL
GOTERM_CC_FAT	GO:0043228~non-membrane-bounded organelle	4	4	0,870	INCENP, LOC431317, LOC395772, NEFL

**Annotation Cluster 54**	**Regulation of transcription and biosynthesis**	**Enrichment Score: 0,47091460501370447**

Category	Term	Count	%	PValue	Genes
GOTERM_BP_ALL	GO:0031323~regulation of cellular metabolic process	25	26	0,191	BACH1, PPARA, E2F3, PARD3, MLH1, EHF, GRAMD4, TNFRSF1B, BCL9L, NRG1, ERF, IL6, GIMAP5, KLF9, DGKI, SPEN, FOXP1, DVL1, SAFB2, NCOA3, DIO2, VOPP1, SMARCA1, RBM15, IGFBP5
GOTERM_BP_ALL	GO:0019222~regulation of metabolic process	25	26	0,262	BACH1, PPARA, E2F3, PARD3, MLH1, EHF, GRAMD4, TNFRSF1B, BCL9L, NRG1, ERF, IL6, GIMAP5, KLF9, DGKI, SPEN, FOXP1, DVL1, SAFB2, NCOA3, DIO2, VOPP1, SMARCA1, RBM15, IGFBP5
GOTERM_BP_ALL	GO:0051171~regulation of nitrogen compound metabolic process	20	21	0,283	BACH1, ERF, PPARA, E2F3, IL6, GIMAP5, KLF9, MLH1, EHF, SPEN, GRAMD4, FOXP1, SAFB2, TNFRSF1B, NCOA3, BCL9L, VOPP1, SMARCA1, NRG1, RBM15
GOTERM_BP_ALL	GO:0010468~regulation of gene expression	20	21	0,293	BACH1, ERF, PPARA, E2F3, IL6, KLF9, EHF, SPEN, GRAMD4, FOXP1, SAFB2, TNFRSF1B, DIO2, NCOA3, BCL9L, VOPP1, SMARCA1, NRG1, RBM15, IGFBP5
GOTERM_BP_ALL	GO:0031326~regulation of cellular biosynthetic process	20	21	0,345	BACH1, ERF, PPARA, E2F3, IL6, GIMAP5, KLF9, EHF, SPEN, GRAMD4, FOXP1, SAFB2, DIO2, NCOA3, BCL9L, VOPP1, SMARCA1, NRG1, RBM15, IGFBP5
GOTERM_BP_ALL	GO:0080090~regulation of primary metabolic process	22	23	0,351	BACH1, ERF, PPARA, E2F3, IL6, GIMAP5, KLF9, MLH1, EHF, SPEN, GRAMD4, FOXP1, SAFB2, TNFRSF1B, DIO2, NCOA3, BCL9L, VOPP1, SMARCA1, NRG1, RBM15, IGFBP5
GOTERM_BP_ALL	GO:0009889~regulation of biosynthetic process	20	21	0,356	BACH1, ERF, PPARA, E2F3, IL6, GIMAP5, KLF9, EHF, SPEN, GRAMD4, FOXP1, SAFB2, DIO2, NCOA3, BCL9L, VOPP1, SMARCA1, NRG1, RBM15, IGFBP5
GOTERM_BP_ALL	GO:0019219~regulation of nucleobase, nucleoside, nucleotide and nucleic acid metabolic process	19	20	0,365	BACH1, ERF, PPARA, E2F3, IL6, KLF9, MLH1, EHF, SPEN, GRAMD4, FOXP1, SAFB2, TNFRSF1B, NCOA3, BCL9L, VOPP1, SMARCA1, NRG1, RBM15
GOTERM_BP_ALL	GO:0010556~regulation of macromolecule biosynthetic process	19	20	0,375	BACH1, ERF, PPARA, E2F3, IL6, KLF9, EHF, SPEN, GRAMD4, FOXP1, SAFB2, DIO2, NCOA3, BCL9L, VOPP1, SMARCA1, NRG1, RBM15, IGFBP5
GOTERM_BP_ALL	GO:0060255~regulation of macromolecule metabolic process	21	22	0,432	BACH1, ERF, PPARA, E2F3, IL6, KLF9, MLH1, EHF, SPEN, GRAMD4, FOXP1, SAFB2, TNFRSF1B, DIO2, NCOA3, BCL9L, VOPP1, SMARCA1, NRG1, RBM15, IGFBP5
GOTERM_BP_ALL	GO:0045449~regulation of transcription	17	18	0,444	BACH1, ERF, PPARA, E2F3, IL6, KLF9, EHF, SPEN, GRAMD4, FOXP1, SAFB2, NCOA3, BCL9L, VOPP1, SMARCA1, NRG1, RBM15

GOTERM_BP_FAT	GO:0045449~regulation of transcription	17	18	0,475	BACH1, ERF, PPARA, E2F3, IL6, KLF9, EHF, SPEN, GRAMD4, FOXP1, SAFB2, NCOA3, BCL9L, VOPP1, SMARCA1, NRG1, RBM15

**Annotation Cluster 55**	**Regulation of apoptosis**	**Enrichment Score: 0,47038758016386656**			
Category	Term	Count	%	PValue	Genes
GOTERM_BP_ALL	GO:0042981~regulation of apoptosis	7	7	0,324	IL6, GIMAP5, GRID2, MLH1, RRM2B, NRG1, NEFL
GOTERM_BP_ALL	GO:0043067~regulation of programmed cell death	7	7	0,333	IL6, GIMAP5, GRID2, MLH1, RRM2B, NRG1, NEFL
GOTERM_BP_ALL	GO:0010941~regulation of cell death	7	7	0,336	IL6, GIMAP5, GRID2, MLH1, RRM2B, NRG1, NEFL
GOTERM_BP_FAT	GO:0042981~regulation of apoptosis	7	7	0,340	IL6, GIMAP5, GRID2, MLH1, RRM2B, NRG1, NEFL
GOTERM_BP_FAT	GO:0043067~regulation of programmed cell death	7	7	0,348	IL6, GIMAP5, GRID2, MLH1, RRM2B, NRG1, NEFL
GOTERM_BP_FAT	GO:0010941~regulation of cell death	7	7	0,351	IL6, GIMAP5, GRID2, MLH1, RRM2B, NRG1, NEFL

**Annotation Cluster 56**	**Membrane**	**Enrichment Score: 0,46633782718299915**			
Category	Term	Count	%	PValue	Genes
SP_PIR_KEYWORDS	transmembrane	10	11	0,052	ST3GAL1, BLB1, LETM1, ST3GAL5, DIO2, DIO3, CD69, SLC6A6, SLC24A1, NRG1
UP_SEQ_FEATURE	transmembrane region	6	6	0,110	ST3GAL1, LETM1, DIO2, DIO3, SLC24A1, NRG1
SP_PIR_KEYWORDS	membrane	9	9	0,179	ST3GAL1, LETM1, ST3GAL5, DIO2, DIO3, CD69, SLC6A6, SLC24A1, NRG1
UP_SEQ_FEATURE	topological domain:Extracellular	3	3	0,466	DIO3, SLC24A1, NRG1
UP_SEQ_FEATURE	topological domain:Cytoplasmic	3	3	0,600	ST3GAL1, SLC24A1, NRG1
UP_SEQ_FEATURE	glycosylation site:N-linked (GlcNAc, , ,)	3	3	0,786	ST3GAL1, SLC24A1, NRG1
SP_PIR_KEYWORDS	glycoprotein	3	3	0,832	ST3GAL1, SLC24A1, NRG1
SP_COMMENT_TYPE	subcellular location	8	8	0,994	ST3GAL1, DIO2, DIO3, INCENP, SLC24A1, DNAJC3, NRG1, FOXP1

**Annotation Cluster 57**	**Kinase activity**	**Enrichment Score: 0,46628495846808665**			
Category	Term	Count	%	PValue	Genes
SP_PIR_KEYWORDS	kinase	6	6	0,260	TPK1, MAP3K3, SPEG, PDK4, DGKI, PIK3R4
GOTERM_MF_ALL	GO:0016301~kinase activity	7	7	0,317	TPK1, CAV2, MAP3K3, SPEG, PDK4, DGKI, PIK3R4
GOTERM_MF_ALL	GO:0016773~phosphotransferase activity, alcohol group as acceptor	6	6	0,371	CAV2, MAP3K3, SPEG, PDK4, DGKI, PIK3R4

GOTERM_MF_ALL	GO:0016772~transferase activity, transferring phosphorus-containing groups	7	7	0,447	TPK1, CAV2, MAP3K3, SPEG, PDK4, DGKI, PIK3R4

**Annotation Cluster 58**	**Regulation of protein metabolism**	**Enrichment Score: 0,4567739984305782**			
Category	Term	Count	%	PValue	Genes
GOTERM_BP_ALL	GO:0032147~activation of protein kinase activity	3	3	0,142	PARD3, DGKI, NRG1
GOTERM_BP_FAT	GO:0032147~activation of protein kinase activity	3	3	0,146	PARD3, DGKI, NRG1
GOTERM_BP_ALL	GO:0042325~regulation of phosphorylation	5	5	0,286	PARD3, IL6, DGKI, NRG1, DVL1
GOTERM_BP_FAT	GO:0042325~regulation of phosphorylation	5	5	0,297	PARD3, IL6, DGKI, NRG1, DVL1
GOTERM_BP_ALL	GO:0051174~regulation of phosphorus metabolic process	5	5	0,311	PARD3, IL6, DGKI, NRG1, DVL1
GOTERM_BP_ALL	GO:0019220~regulation of phosphate metabolic process	5	5	0,311	PARD3, IL6, DGKI, NRG1, DVL1
GOTERM_BP_FAT	GO:0019220~regulation of phosphate metabolic process	5	5	0,323	PARD3, IL6, DGKI, NRG1, DVL1
GOTERM_BP_FAT	GO:0051174~regulation of phosphorus metabolic process	5	5	0,323	PARD3, IL6, DGKI, NRG1, DVL1
GOTERM_BP_ALL	GO:0045859~regulation of protein kinase activity	4	4	0,324	PARD3, DGKI, NRG1, DVL1
GOTERM_BP_FAT	GO:0045859~regulation of protein kinase activity	4	4	0,334	PARD3, DGKI, NRG1, DVL1
GOTERM_BP_ALL	GO:0043549~regulation of kinase activity	4	4	0,344	PARD3, DGKI, NRG1, DVL1
GOTERM_BP_FAT	GO:0043549~regulation of kinase activity	4	4	0,354	PARD3, DGKI, NRG1, DVL1
GOTERM_BP_ALL	GO:0051338~regulation of transferase activity	4	4	0,367	PARD3, DGKI, NRG1, DVL1
GOTERM_BP_ALL	GO:0045860~positive regulation of protein kinase activity	3	3	0,373	PARD3, DGKI, NRG1
GOTERM_BP_FAT	GO:0051338~regulation of transferase activity	4	4	0,378	PARD3, DGKI, NRG1, DVL1
GOTERM_BP_FAT	GO:0045860~positive regulation of protein kinase activity	3	3	0,381	PARD3, DGKI, NRG1

GOTERM_BP_ALL	GO:0033674~positive regulation of kinase activity	3	3	0,389	PARD3, DGKI, NRG1
GOTERM_BP_FAT	GO:0033674~positive regulation of kinase activity	3	3	0,398	PARD3, DGKI, NRG1
GOTERM_BP_ALL	GO:0051347~positive regulation of transferase activity	3	3	0,408	PARD3, DGKI, NRG1
GOTERM_BP_FAT	GO:0051347~positive regulation of transferase activity	3	3	0,417	PARD3, DGKI, NRG1
GOTERM_BP_ALL	GO:0043085~positive regulation of catalytic activity	4	4	0,586	CASR, PARD3, DGKI, NRG1
GOTERM_BP_FAT	GO:0043085~positive regulation of catalytic activity	4	4	0,599	CASR, PARD3, DGKI, NRG1
GOTERM_BP_ALL	GO:0050790~regulation of catalytic activity	5	5	0,732	CASR, PARD3, DGKI, NRG1, DVL1

**Annotation Cluster 59**	**Ank repeat**	**Enrichment Score: 0,45336078380375283**			
Category	Term	Count	%	PValue	Genes
UP_SEQ_FEATURE	repeat:ANK 1	3	3	0,323	MIB1, ANK3, DGKI
UP_SEQ_FEATURE	repeat:ANK 2	3	3	0,325	MIB1, ANK3, DGKI
SP_PIR_KEYWORDS	ank repeat	3	3	0,337	MIB1, ANK3, DGKI
INTERPRO	IPR002110:Ankyrin	3	3	0,359	MIB1, ANK3, DGKI
SMART	SM00248:ANK	3	3	0,425	MIB1, ANK3, DGKI

**Annotation Cluster 60**	**RNA binding**	**Enrichment Score: 0,452298582118472**			
Category	Term	Count	%	PValue	Genes
UP_SEQ_FEATURE	compositionally biased region:Arg-rich	4	4	0,031	SPEG, SPEN, RBM15, SAFB2
INTERPRO	IPR000504:RNA recognition motif, RNP-1	3	3	0,307	SPEN, RBM15, SAFB2
INTERPRO	IPR012677:Nucleotide-binding, alpha-beta plait	3	3	0,311	SPEN, RBM15, SAFB2
SMART	SM00360:RRM	3	3	0,367	SPEN, RBM15, SAFB2
SP_PIR_KEYWORDS	rna-binding	3	3	0,756	SPEN, RBM15, SAFB2
GOTERM_MF_ALL	GO:0003723~RNA binding	3	3	0,913	SPEN, RBM15, SAFB2
GOTERM_MF_FAT	GO:0003723~RNA binding	3	3	0,925	SPEN, RBM15, SAFB2

**Annotation Cluster 61**	**Axon**	**Enrichment Score: 0,44369216299423014**			
Category	Term	Count	%	PValue	Genes
GOTERM_CC_ALL	GO:0030424~axon	3	3	0,220	ANK3, NEFL, DVL1

GOTERM_CC_FAT	GO:0030424~axon	3	3	0,230	ANK3, NEFL, DVL1
GOTERM_CC_ALL	GO:0043005~neuron projection	3	3	0,567	ANK3, NEFL, DVL1
GOTERM_CC_FAT	GO:0043005~neuron projection	3	3	0,586	ANK3, NEFL, DVL1

**Annotation Cluster 62**	**Response to hormones**	**Enrichment Score: 0,4338809475338149**			
Category	Term	Count	%	PValue	Genes
GOTERM_BP_ALL	GO:0043434~response to peptide hormone stimulus	3	3	0,225	CAV2, PPARA, NEFL
GOTERM_BP_FAT	GO:0043434~response to peptide hormone stimulus	3	3	0,231	CAV2, PPARA, NEFL
GOTERM_BP_ALL	GO:0048545~response to steroid hormone stimulus	3	3	0,307	CAV2, IL6, NEFL
GOTERM_BP_FAT	GO:0048545~response to steroid hormone stimulus	3	3	0,314	CAV2, IL6, NEFL
GOTERM_BP_ALL	GO:0009725~response to hormone stimulus	4	4	0,360	CAV2, PPARA, IL6, NEFL
GOTERM_BP_FAT	GO:0009725~response to hormone stimulus	4	4	0,370	CAV2, PPARA, IL6, NEFL
GOTERM_BP_ALL	GO:0009719~response to endogenous stimulus	4	4	0,419	CAV2, PPARA, IL6, NEFL
GOTERM_BP_FAT	GO:0009719~response to endogenous stimulus	4	4	0,431	CAV2, PPARA, IL6, NEFL
GOTERM_BP_ALL	GO:0010033~response to organic substance	5	5	0,609	CAV2, PPARA, IL6, DNAJC3, NEFL
GOTERM_BP_FAT	GO:0010033~response to organic substance	5	5	0,623	CAV2, PPARA, IL6, DNAJC3, NEFL

**Annotation Cluster 63**	**Fatty acid metabolic process**	**Enrichment Score: 0,4199872542870759**			
Category	Term	Count	%	PValue	Genes
GOTERM_BP_ALL	GO:0006631~fatty acid metabolic process	3	3	0,320	PPARA, FASN, ACSL6
GOTERM_BP_FAT	GO:0006631~fatty acid metabolic process	3	3	0,327	PPARA, FASN, ACSL6
GOTERM_BP_ALL	GO:0032787~monocarboxylic acid metabolic process	3	3	0,525	PPARA, FASN, ACSL6

**Annotation Cluster 64**	**Extracellular matrix**	**Enrichment Score: 0,4063740893246793**			
Category	Term	Count	%	PValue	Genes
GOTERM_CC_ALL	GO:0031012~extracellular matrix	4	4	0,298	ADAMTSL1, COL13A1, OLFML2A, ADAMTS5

GOTERM_CC_FAT	GO:0031012~extracellular matrix	4	4	0,315	ADAMTSL1, COL13A1, OLFML2A, ADAMTS5
SP_PIR_KEYWORDS	extracellular matrix	3	3	0,339	ADAMTSL1, COL13A1, ADAMTS5
GOTERM_CC_ALL	GO:0005578~proteinaceous extracellular matrix	3	3	0,531	ADAMTSL1, COL13A1, ADAMTS5
GOTERM_CC_FAT	GO:0005578~proteinaceous extracellular matrix	3	3	0,549	ADAMTSL1, COL13A1, ADAMTS5

**Annotation Cluster 65**	**Membrane**	**Enrichment Score: 0,3978557053344479**			
Category	Term	Count	%	PValue	Genes
SP_PIR_KEYWORDS	membrane	37	38	0,175	CAV2, PARD3, LIMS1, CASR, PLXNA2, LRBA, MITD1, GRAMD4, ST3GAL1, TNFRSF1B, ST3GAL5, ITPRIP, CD69, NUMB, SLC24A1, GRID2, NRG1, IFNGR2, ACSL6, C10ORF72, GIMAP5, MMEL1, NRXN3, COL13A1, LRRC1, NLGN1, GOLIM4, IGSF9B, FAM123B, P2RY12, MIB1, LETM1, DIO2, PTP4A3, DIO3, SLC6A6, VOPP1
GOTERM_CC_ALL	GO:0016020~membrane	41	42	0,558	CAV2, PARD3, LIMS1, CASR, PLXNA2, LRBA, AHCTF1, MITD1, GRAMD4, ST3GAL1, TNFRSF1B, ST3GAL5, ITPRIP, ANK3, CD69, NUMB, SLC24A1, GRID2, NRG1, IFNGR2, ACSL6, C10ORF72, IL6, GIMAP5, MMEL1, NRXN3, COL13A1, LRRC1, PDK4, NLGN1, GOLIM4, OLFML2A, IGSF9B, P2RY12, MIB1, LETM1, DIO2, PTP4A3, DIO3, SLC6A6, VOPP1
GOTERM_CC_ALL	GO:0044425~membrane part	36	37	0,657	CAV2, PARD3, LIMS1, CASR, PLXNA2, LRBA, AHCTF1, MITD1, GRAMD4, ST3GAL1, TNFRSF1B, ST3GAL5, ANK3, CD69, NUMB, SLC24A1, GRID2, NRG1, IFNGR2, ACSL6, C10ORF72, IL6, GIMAP5, MMEL1, NRXN3, COL13A1, LRRC1, NLGN1, GOLIM4, IGSF9B, P2RY12, LETM1, DIO2, DIO3, SLC6A6, VOPP1

**Annotation Cluster 66**	**Immune system development and activation**	**Enrichment Score: 0,3945323276117384**			
Category	Term	Count	%	PValue	Genes
GOTERM_BP_ALL	GO:0046649~lymphocyte activation	3	3	0,322	GIMAP5, MLH1, FOXP1
GOTERM_BP_FAT	GO:0046649~lymphocyte activation	3	3	0,330	GIMAP5, MLH1, FOXP1
GOTERM_BP_ALL	GO:0045321~leukocyte activation	3	3	0,412	GIMAP5, MLH1, FOXP1
GOTERM_BP_FAT	GO:0045321~leukocyte activation	3	3	0,421	GIMAP5, MLH1, FOXP1
GOTERM_BP_ALL	GO:0002520~immune system development	3	3	0,479	GIMAP5, MLH1, FOXP1

GOTERM_BP_FAT	GO:0002520~immune system development	3	3	0,488	GIMAP5, MLH1, FOXP1

Annotation Cluster 67	Metal ion binding	Enrichment Score: 0,3827423919924523			
Category	Term	Count	%	PValue	Genes
GOTERM_MF_ALL	GO:0046872~metal ion binding	26	27	0,347	PPARA, LIMS1, GDA, ADAMTSL1, PPP2R3A, FAM20C, RRM2B, CALB1, MAP3K3, CD69, SLC24A1, FASN, PLCB1, ACSL6, KLF9, MMEL1, NRXN3, SMYD3, DGKI, ARMC1, FOXP1, NEBL, MIB1, LETM1, ADAMTS5, USP44
GOTERM_MF_ALL	GO:0043169~cation binding	26	27	0,367	PPARA, LIMS1, GDA, ADAMTSL1, PPP2R3A, FAM20C, RRM2B, CALB1, MAP3K3, CD69, SLC24A1, FASN, PLCB1, ACSL6, KLF9, MMEL1, NRXN3, SMYD3, DGKI, ARMC1, FOXP1, NEBL, MIB1, LETM1, ADAMTS5, USP44
GOTERM_MF_ALL	GO:0043167~ion binding	26	27	0,400	PPARA, LIMS1, GDA, ADAMTSL1, PPP2R3A, FAM20C, RRM2B, CALB1, MAP3K3, CD69, SLC24A1, FASN, PLCB1, ACSL6, KLF9, MMEL1, NRXN3, SMYD3, DGKI, ARMC1, FOXP1, NEBL, MIB1, LETM1, ADAMTS5, USP44
GOTERM_MF_FAT	GO:0046872~metal ion binding	26	27	0,437	PPARA, LIMS1, GDA, ADAMTSL1, PPP2R3A, FAM20C, RRM2B, CALB1, MAP3K3, CD69, SLC24A1, FASN, PLCB1, ACSL6, KLF9, MMEL1, NRXN3, SMYD3, DGKI, ARMC1, FOXP1, NEBL, MIB1, LETM1, ADAMTS5, USP44
GOTERM_MF_FAT	GO:0043169~cation binding	26	27	0,459	PPARA, LIMS1, GDA, ADAMTSL1, PPP2R3A, FAM20C, RRM2B, CALB1, MAP3K3, CD69, SLC24A1, FASN, PLCB1, ACSL6, KLF9, MMEL1, NRXN3, SMYD3, DGKI, ARMC1, FOXP1, NEBL, MIB1, LETM1, ADAMTS5, USP44
GOTERM_MF_FAT	GO:0043167~ion binding	26	27	0,494	PPARA, LIMS1, GDA, ADAMTSL1, PPP2R3A, FAM20C, RRM2B, CALB1, MAP3K3, CD69, SLC24A1, FASN, PLCB1, ACSL6, KLF9, MMEL1, NRXN3, SMYD3, DGKI, ARMC1, FOXP1, NEBL, MIB1, LETM1, ADAMTS5, USP44

Annotation Cluster 68	Cell adhesion	Enrichment Score: 0,3705962723675147			
Category	Term	Count	%	PValue	Genes
GOTERM_BP_ALL	GO:0016337~cell-cell adhesion	4	4	0,216	PARD3, LIMS1, COL13A1, NLGN1
GOTERM_BP_FAT	GO:0016337~cell-cell adhesion	4	4	0,224	PARD3, LIMS1, COL13A1, NLGN1
GOTERM_BP_ALL	GO:0007155~cell adhesion	5	5	0,585	PARD3, LIMS1, COL13A1, NRXN3, NLGN1
GOTERM_BP_ALL	GO:0022610~biological adhesion	5	5	0,586	PARD3, LIMS1, COL13A1, NRXN3, NLGN1
GOTERM_BP_FAT	GO:0007155~cell adhesion	5	5	0,600	PARD3, LIMS1, COL13A1, NRXN3, NLGN1

GOTERM_BP_FAT	GO:0022610~biological adhesion	5	5	0,601	PARD3, LIMS1, COL13A1, NRXN3, NLGN1

**Annotation Cluster 69**	**Transcription activity and binding**	**Enrichment Score: 0,36152875448816824**			
Category	Term	Count	%	PValue	Genes
GOTERM_MF_ALL	GO:0003712~transcription cofactor activity	4	4	0,327	ERF, NCOA3, SPEN, NRG1
GOTERM_MF_FAT	GO:0003712~transcription cofactor activity	4	4	0,350	ERF, NCOA3, SPEN, NRG1
GOTERM_MF_ALL	GO:0008134~transcription factor binding	4	4	0,545	ERF, NCOA3, SPEN, NRG1
GOTERM_MF_FAT	GO:0008134~transcription factor binding	4	4	0,574	ERF, NCOA3, SPEN, NRG1

**Annotation Cluster 70**	**Immune response**	**Enrichment Score: 0,35182272937712755**			
Category	Term	Count	%	PValue	Genes
GOTERM_BP_ALL	GO:0002252~immune effector process	3	3	0,183	IL6, MLH1, FOXP1
GOTERM_BP_FAT	GO:0002252~immune effector process	3	3	0,188	IL6, MLH1, FOXP1
GOTERM_BP_ALL	GO:0006955~immune response	4	4	0,771	TNFRSF1B, IL6, MLH1, FOXP1
GOTERM_BP_FAT	GO:0006955~immune response	4	4	0,782	TNFRSF1B, IL6, MLH1, FOXP1
GOTERM_BP_ALL	GO:0002376~immune system process	5	5	0,841	TNFRSF1B, IL6, GIMAP5, MLH1, FOXP1

**Annotation Cluster 71**	**Cell develpment, morphogenesis and differentiation**	**Enrichment Score: 0,3430737654638174**			
Category	Term	Count	%	PValue	Genes
GOTERM_BP_FAT	GO:0000902~cell morphogenesis	3	3	0,167	ANK3, NUMB, NRG1
GOTERM_BP_ALL	GO:0000902~cell morphogenesis	3	3	0,168	ANK3, NUMB, NRG1
GOTERM_BP_FAT	GO:0032989~cellular component morphogenesis	3	3	0,210	ANK3, NUMB, NRG1
GOTERM_BP_ALL	GO:0032989~cellular component morphogenesis	3	3	0,211	ANK3, NUMB, NRG1
GOTERM_BP_FAT	GO:0030182~neuron differentiation	3	3	0,254	ANK3, NUMB, NRG1
GOTERM_BP_ALL	GO:0030182~neuron differentiation	3	3	0,255	ANK3, NUMB, NRG1
GOTERM_CC_ALL	GO:0044459~plasma membrane part	5	5	0,291	IL6, BLB1, NUMB, SLC6A6, NRG1
GOTERM_CC_FAT	GO:0044459~plasma membrane part	5	5	0,340	IL6, BLB1, NUMB, SLC6A6, NRG1
GOTERM_BP_ALL	GO:0048699~generation of neurons	3	3	0,352	ANK3, NUMB, NRG1
GOTERM_BP_ALL	GO:0022008~neurogenesis	3	3	0,382	ANK3, NUMB, NRG1
GOTERM_CC_ALL	GO:0005576~extracellular region	5	5	0,418	ST3GAL1, IL6, NRG1, ADAMTS5, IGFBP5
GOTERM_CC_ALL	GO:0005886~plasma membrane	6	6	0,444	IL6, BLB1, DIO3, NUMB, SLC6A6, NRG1
GOTERM_CC_FAT	GO:0005576~extracellular region	5	5	0,479	ST3GAL1, IL6, NRG1, ADAMTS5, IGFBP5
GOTERM_BP_ALL	GO:0016043~cellular component organization	6	6	0,500	LETM1, ANK3, INCENP, NUMB, NRG1, SMARCA1

GOTERM_CC_FAT	GO:0005886~plasma membrane	6	6	0,517	IL6, BLB1, DIO3, NUMB, SLC6A6, NRG1
GOTERM_BP_ALL	GO:0009653~anatomical structure morphogenesis	4	4	0,550	IL6, ANK3, NUMB, NRG1
GOTERM_BP_ALL	GO:0007399~nervous system development	3	3	0,632	ANK3, NUMB, NRG1
GOTERM_BP_ALL	GO:0032501~multicellular organismal process	8	8	0,659	IL6, ANK3, PLXNA2, NUMB, SLC24A1, NRG1, CALB1, IGFBP5
GOTERM_BP_ALL	GO:0030154~cell differentiation	4	4	0,684	ANK3, NUMB, NRG1, IGFBP5
GOTERM_BP_ALL	GO:0048869~cellular developmental process	4	4	0,706	ANK3, NUMB, NRG1, IGFBP5
GOTERM_BP_ALL	GO:0010646~regulation of cell communication	3	3	0,709	IL6, NRG1, IGFBP5
GOTERM_BP_ALL	GO:0048731~system development	5	5	0,712	IL6, ANK3, NUMB, NRG1, IGFBP5
GOTERM_BP_ALL	GO:0007275~multicellular organismal development	6	6	0,752	IL6, ANK3, PLXNA2, NUMB, NRG1, IGFBP5
GOTERM_BP_ALL	GO:0048856~anatomical structure development	5	5	0,766	IL6, ANK3, NUMB, NRG1, IGFBP5
GOTERM_BP_ALL	GO:0032502~developmental process	6	6	0,811	IL6, ANK3, PLXNA2, NUMB, NRG1, IGFBP5
GOTERM_BP_ALL	GO:0048513~organ development	3	3	0,921	IL6, NUMB, NRG1
GOTERM_BP_ALL	GO:0048518~positive regulation of biological process	3	3	0,927	IL6, DIO3, NRG1

**Annotation Cluster 72**	**Cell division**	**Enrichment Score: 0,32992726469452366**			
Category	Term	Count	%	PValue	Genes
SP_PIR_KEYWORDS	cell division	3	3	0,380	PARD3, INCENP, AHCTF1
GOTERM_BP_ALL	GO:0051301~cell division	3	3	0,514	PARD3, INCENP, AHCTF1
GOTERM_BP_FAT	GO:0051301~cell division	3	3	0,524	PARD3, INCENP, AHCTF1

**Annotation Cluster 73**	**Mitochondria**	**Enrichment Score: 0,3289973037852026**			
Category	Term	Count	%	PValue	Genes
GOTERM_CC_ALL	GO:0031966~mitochondrial membrane	4	4	0,373	LETM1, GIMAP5, PDK4, ACSL6
GOTERM_CC_FAT	GO:0031966~mitochondrial membrane	4	4	0,392	LETM1, GIMAP5, PDK4, ACSL6
GOTERM_CC_ALL	GO:0005740~mitochondrial envelope	4	4	0,410	LETM1, GIMAP5, PDK4, ACSL6
GOTERM_CC_FAT	GO:0005740~mitochondrial envelope	4	4	0,430	LETM1, GIMAP5, PDK4, ACSL6
GOTERM_CC_ALL	GO:0031967~organelle envelope	5	5	0,450	LETM1, GIMAP5, PDK4, AHCTF1, ACSL6
GOTERM_CC_ALL	GO:0031975~envelope	5	5	0,453	LETM1, GIMAP5, PDK4, AHCTF1, ACSL6
GOTERM_CC_FAT	GO:0031967~organelle envelope	5	5	0,475	LETM1, GIMAP5, PDK4, AHCTF1, ACSL6

GOTERM_CC_FAT	GO:0031975~envelope	5	5	0,478	LETM1, GIMAP5, PDK4, AHCTF1, ACSL6
GOTERM_CC_ALL	GO:0044429~mitochondrial part	4	4	0,645	LETM1, GIMAP5, PDK4, ACSL6
GOTERM_CC_FAT	GO:0044429~mitochondrial part	4	4	0,667	LETM1, GIMAP5, PDK4, ACSL6
**Annotation Cluster 74**	**Transcription regulation**	**Enrichment Score: 0,32514922741950214**			
Category	Term	Count	%	PValue	Genes
SP_PIR_KEYWORDS	transcription regulation	13	13	0,310	BACH1, PPARA, ERF, E2F3, KLF9, EHF, SPEN, FOXP1, SAFB2, NCOA3, BCL9L, VOPP1, SMARCA1
SP_PIR_KEYWORDS	Transcription	13	13	0,337	BACH1, PPARA, ERF, E2F3, KLF9, EHF, SPEN, FOXP1, SAFB2, NCOA3, BCL9L, VOPP1, SMARCA1
GOTERM_BP_ALL	GO:0009058~biosynthetic process	23	24	0,399	BACH1, ERF, PPARA, E2F3, KLF9, AHCTF1, EHF, RRM2B, SPEN, FOXP1, SAFB2, TPK1, ST3GAL1, DIO2, ST3GAL5, NCOA3, DIO3, FASN, BCL9L, VOPP1, SMARCA1, SUCLA2, ADC
GOTERM_BP_ALL	GO:0044249~cellular biosynthetic process	22	23	0,440	BACH1, ERF, PPARA, E2F3, KLF9, AHCTF1, EHF, RRM2B, SPEN, FOXP1, SAFB2, TPK1, ST3GAL1, DIO2, ST3GAL5, NCOA3, FASN, BCL9L, VOPP1, SMARCA1, SUCLA2, ADC
GOTERM_BP_ALL	GO:0006350~transcription	14	14	0,449	BACH1, ERF, PPARA, E2F3, KLF9, AHCTF1, EHF, SPEN, FOXP1, SAFB2, NCOA3, BCL9L, VOPP1, SMARCA1
GOTERM_BP_ALL	GO:0034645~cellular macromolecule biosynthetic process	18	19	0,471	BACH1, ERF, PPARA, E2F3, KLF9, AHCTF1, EHF, RRM2B, SPEN, FOXP1, SAFB2, ST3GAL1, ST3GAL5, NCOA3, DIO2, BCL9L, VOPP1, SMARCA1
GOTERM_BP_FAT	GO:0006350~transcription	14	14	0,477	BACH1, ERF, PPARA, E2F3, KLF9, AHCTF1, EHF, SPEN, FOXP1, SAFB2, NCOA3, BCL9L, VOPP1, SMARCA1
GOTERM_BP_ALL	GO:0009059~macromolecule biosynthetic process	18	19	0,484	BACH1, ERF, PPARA, E2F3, KLF9, AHCTF1, EHF, RRM2B, SPEN, FOXP1, SAFB2, ST3GAL1, ST3GAL5, NCOA3, DIO2, BCL9L, VOPP1, SMARCA1
GOTERM_BP_ALL	GO:0010467~gene expression	16	16	0,783	BACH1, ERF, PPARA, E2F3, KLF9, MLH1, AHCTF1, EHF, SPEN, FOXP1, SAFB2, NCOA3, DIO2, BCL9L, VOPP1, SMARCA1
GOTERM_BP_ALL	GO:0006139~nucleobase, nucleoside, nucleotide and nucleic acid metabolic process	18	19	0,802	BACH1, ERF, PPARA, E2F3, GDA, KLF9, MLH1, AHCTF1, UPP2, EHF, RRM2B, SPEN, FOXP1, SAFB2, NCOA3, BCL9L, VOPP1, SMARCA1
**Annotation Cluster 75**	**Organelle**	**Enrichment Score: 0,30277630334524575**			
Category	Term	Count	%	PValue	Genes
GOTERM_CC_ALL	GO:0043228~non-membrane-bounded organelle	16	16	0,471	BACH1, PARD3, NLGN1, MLH1, AHCTF1, UPP2, SPEN, FOXP1, DVL1, RIF1, NEB, ANK3, TPPP, INCENP, GRID2, NEFL

GOTERM_CC_ALL	GO:0043232~intracellular non-membrane-bounded organelle	16	16	0,471	BACH1, PARD3, NLGN1, MLH1, AHCTF1, UPP2, SPEN, FOXP1, DVL1, RIF1, NEB, ANK3, TPPP, INCENP, GRID2, NEFL
GOTERM_CC_FAT	GO:0043232~intracellular non-membrane-bounded organelle	16	16	0,526	BACH1, PARD3, NLGN1, MLH1, AHCTF1, UPP2, SPEN, FOXP1, DVL1, RIF1, NEB, ANK3, TPPP, INCENP, GRID2, NEFL
GOTERM_CC_FAT	GO:0043228~non-membrane-bounded organelle	16	16	0,526	BACH1, PARD3, NLGN1, MLH1, AHCTF1, UPP2, SPEN, FOXP1, DVL1, RIF1, NEB, ANK3, TPPP, INCENP, GRID2, NEFL

**Annotation Cluster 76**	**Neurological system processes**	**Enrichment Score: 0,2998971437634362**			
Category	Term	Count	%	PValue	Genes
GOTERM_BP_FAT	GO:0050877~neurological system process	3	3	0,345	SLC24A1, NRG1, CALB1
GOTERM_BP_ALL	GO:0050877~neurological system process	3	3	0,347	SLC24A1, NRG1, CALB1
GOTERM_BP_ALL	GO:0050896~response to stimulus	7	7	0,439	IL6, CASR, BLB1, SLC24A1, DNAJC3, NRG1, CALB1
GOTERM_BP_ALL	GO:0003008~system process	3	3	0,504	SLC24A1, NRG1, CALB1
GOTERM_BP_ALL	GO:0032501~multicellular organismal process	8	8	0,659	IL6, ANK3, PLXNA2, NUMB, SLC24A1, NRG1, CALB1, IGFBP5
GOTERM_BP_ALL	GO:0051179~localization	7	7	0,910	IL6, SLC6A6, SLC24A1, GRID2, PITPNC1, NRG1, CALB1

**Annotation Cluster 77**	**Peptidase activity**	**Enrichment Score: 0,29952905696399174**			
Category	Term	Count	%	PValue	Genes
GOTERM_MF_ALL	GO:0008237~metallopeptidase activity	3	3	0,270	ADAMTSL1, MMEL1, ADAMTS5
GOTERM_MF_FAT	GO:0008237~metallopeptidase activity	3	3	0,285	ADAMTSL1, MMEL1, ADAMTS5
GOTERM_MF_ALL	GO:0070011~peptidase activity, acting on L-amino acid peptides	4	4	0,592	ADAMTSL1, MMEL1, ADAMTS5, USP44
GOTERM_MF_FAT	GO:0070011~peptidase activity, acting on L-amino acid peptides	4	4	0,620	ADAMTSL1, MMEL1, ADAMTS5, USP44
GOTERM_MF_ALL	GO:0008233~peptidase activity	4	4	0,622	ADAMTSL1, MMEL1, ADAMTS5, USP44
GOTERM_MF_FAT	GO:0008233~peptidase activity	4	4	0,650	ADAMTSL1, MMEL1, ADAMTS5, USP44
SP_PIR_KEYWORDS	Protease	3	3	0,700	MMEL1, ADAMTS5, USP44

**Annotation Cluster 78**	**Cell motility**	**Enrichment Score: 0,29123959234613295**			
Category	Term	Count	%	PValue	Genes
GOTERM_BP_ALL	GO:0040011~locomotion	4	4	0,459	CAV2, IL6, PLXNA2, NEFL
GOTERM_BP_ALL	GO:0016477~cell migration	3	3	0,479	CAV2, IL6, PLXNA2
GOTERM_BP_FAT	GO:0016477~cell migration	3	3	0,488	CAV2, IL6, PLXNA2
GOTERM_BP_ALL	GO:0051674~localization of cell	3	3	0,535	CAV2, IL6, PLXNA2
GOTERM_BP_ALL	GO:0048870~cell motility	3	3	0,535	CAV2, IL6, PLXNA2
GOTERM_BP_FAT	GO:0051674~localization of cell	3	3	0,545	CAV2, IL6, PLXNA2

GOTERM_BP_FAT	GO:0048870~cell motility	3	3	0,545	CAV2, IL6, PLXNA2

**Annotation Cluster 79**	**Organelle**	**Enrichment Score: 0,29058479195704773**			
Category	Term	Count	%	PValue	Genes
GOTERM_CC_ALL	GO:0044422~organelle part	10	11	0,191	ST3GAL1, LETM1, E2F3, ST3GAL5, INCENP, LOC431317, SMARCA1, LOC395772, CALB1, NEFL
GOTERM_CC_ALL	GO:0043229~intracellular organelle	18	19	0,443	PPARA, E2F3, CALB1, LOC395772, FOXP1, ST3GAL1, LETM1, NCOA3, ST3GAL5, DIO3, INCENP, NUMB, LOC431317, SUCLA2, DNAJC3, SMARCA1, RBM15, NEFL
GOTERM_CC_ALL	GO:0043226~organelle	18	19	0,446	PPARA, E2F3, CALB1, LOC395772, FOXP1, ST3GAL1, LETM1, NCOA3, ST3GAL5, DIO3, INCENP, NUMB, LOC431317, SUCLA2, DNAJC3, SMARCA1, RBM15, NEFL
GOTERM_CC_ALL	GO:0044464~cell part	36	38	0,489	PPARA, BLB1, E2F3, CASR, PLXNA2, UPP2, LOC395772, CALB1, ST3GAL1, TNFRSF1B, ST3GAL5, ANK3, CD69, GSTK1, INCENP, NUMB, SLC24A1, GRID2, PITPNC1, NRG1, DNAJC3, SUCLA2, NEFL, IL6, NLGN1, OLFML2A, FOXP1, LETM1, NEB, DIO2, NCOA3, DIO3, SLC6A6, LOC431317, SMARCA1, RBM15
GOTERM_CC_ALL	GO:0005623~cell	36	38	0,489	PPARA, BLB1, E2F3, CASR, PLXNA2, UPP2, LOC395772, CALB1, ST3GAL1, TNFRSF1B, ST3GAL5, ANK3, CD69, GSTK1, INCENP, NUMB, SLC24A1, GRID2, PITPNC1, NRG1, DNAJC3, SUCLA2, NEFL, IL6, NLGN1, OLFML2A, FOXP1, LETM1, NEB, DIO2, NCOA3, DIO3, SLC6A6, LOC431317, SMARCA1, RBM15
GOTERM_CC_ALL	GO:0043231~intracellular membrane-bounded organelle	15	16	0,497	PPARA, E2F3, CALB1, FOXP1, ST3GAL1, LETM1, ST3GAL5, NCOA3, DIO3, INCENP, NUMB, SUCLA2, DNAJC3, SMARCA1, RBM15
GOTERM_CC_ALL	GO:0043227~membrane-bounded organelle	15	16	0,500	PPARA, E2F3, CALB1, FOXP1, ST3GAL1, LETM1, ST3GAL5, NCOA3, DIO3, INCENP, NUMB, SUCLA2, DNAJC3, SMARCA1, RBM15
GOTERM_CC_ALL	GO:0044444~cytoplasmic part	8	8	0,592	ST3GAL1, LETM1, ST3GAL5, DIO3, NUMB, DNAJC3, SUCLA2, CALB1
GOTERM_CC_ALL	GO:0044424~intracellular part	20	21	0,612	PPARA, E2F3, UPP2, CALB1, LOC395772, FOXP1, ST3GAL1, LETM1, NCOA3, ST3GAL5, DIO3, INCENP, NUMB, LOC431317, SUCLA2, DNAJC3, SMARCA1, NRG1, RBM15, NEFL
GOTERM_CC_ALL	GO:0005622~intracellular	23	24	0,670	PPARA, E2F3, PLXNA2, UPP2, CALB1, LOC395772, FOXP1, ST3GAL1, LETM1, NCOA3, ST3GAL5, NEB, DIO3, INCENP, NUMB, LOC431317, PITPNC1, SUCLA2, NRG1, SMARCA1, DNAJC3, RBM15, NEFL

GOTERM_CC_ALL	GO:0005634~nucleus	9	9	0,707	PPARA, E2F3, NCOA3, INCENP, NUMB, SMARCA1, CALB1, RBM15, FOXP1
GOTERM_CC_ALL	GO:0005737~cytoplasm	10	11	0,844	ST3GAL1, LETM1, ST3GAL5, DIO3, NUMB, UPP2, DNAJC3, SUCLA2, NRG1, CALB1

**Annotation Cluster 80**	**Post-translational protein modification**	**Enrichment Score: 0,288017720508143**			
Category	Term	Count	%	PValue	Genes
GOTERM_BP_ALL	GO:0006796~phosphate metabolic process	7	7	0,501	CAV2, PPP2R3A, PTP4A3, MAP3K3, SPEG, PDK4, PIK3R4
GOTERM_BP_ALL	GO:0006793~phosphorus metabolic process	7	7	0,501	CAV2, PPP2R3A, PTP4A3, MAP3K3, SPEG, PDK4, PIK3R4
GOTERM_BP_FAT	GO:0006793~phosphorus metabolic process	7	7	0,519	CAV2, PPP2R3A, PTP4A3, MAP3K3, SPEG, PDK4, PIK3R4
GOTERM_BP_FAT	GO:0006796~phosphate metabolic process	7	7	0,519	CAV2, PPP2R3A, PTP4A3, MAP3K3, SPEG, PDK4, PIK3R4
GOTERM_BP_ALL	GO:0043687~post-translational protein modification	8	8	0,536	MIB1, CAV2, PPP2R3A, PTP4A3, MAP3K3, SPEG, PDK4, PIK3R4

**Annotation Cluster 81**	**Protein metabolism**	**Enrichment Score: 0,27982161431894054**			
Category	Term	Count	%	PValue	Genes
GOTERM_MF_ALL	GO:0016773~phosphotransferase activity, alcohol group as acceptor	6	6	0,371	CAV2, MAP3K3, SPEG, PDK4, DGKI, PIK3R4
GOTERM_MF_ALL	GO:0004672~protein kinase activity	5	5	0,435	CAV2, MAP3K3, SPEG, PDK4, PIK3R4
GOTERM_MF_FAT	GO:0004672~protein kinase activity	5	5	0,466	CAV2, MAP3K3, SPEG, PDK4, PIK3R4
UP_SEQ_FEATURE	binding site:ATP	4	4	0,515	MAP3K3, SPEG, PDK4, PIK3R4
GOTERM_BP_ALL	GO:0006468~protein amino acid phosphorylation	5	5	0,546	CAV2, MAP3K3, SPEG, PDK4, PIK3R4
GOTERM_BP_FAT	GO:0006468~protein amino acid phosphorylation	5	5	0,561	CAV2, MAP3K3, SPEG, PDK4, PIK3R4
GOTERM_BP_ALL	GO:0016310~phosphorylation	5	5	0,690	CAV2, MAP3K3, SPEG, PDK4, PIK3R4
GOTERM_BP_FAT	GO:0016310~phosphorylation	5	5	0,704	CAV2, MAP3K3, SPEG, PDK4, PIK3R4

**Annotation Cluster 82**	**Nucleotide binding**	**Enrichment Score: 0,27318634528616514**			
Category	Term	Count	%	PValue	Genes
SP_PIR_KEYWORDS	atp-binding	9	9	0,342	TPK1, MAP3K3, SPEG, PDK4, DGKI, SUCLA2, SMARCA1, PIK3R4, ACSL6
GOTERM_MF_ALL	GO:0005524~ATP binding	10	10	0,436	TPK1, MAP3K3, SPEG, PDK4, MLH1, DGKI, SUCLA2, SMARCA1, PIK3R4, ACSL6

GOTERM_MF_ALL	GO:0032559~adenyl ribonucleotide binding	10	10	0,452	TPK1, MAP3K3, SPEG, PDK4, MLH1, DGKI, SUCLA2, SMARCA1, PIK3R4, ACSL6
SP_PIR_KEYWORDS	nucleotide-binding	10	10	0,468	TPK1, GIMAP5, MAP3K3, SPEG, PDK4, DGKI, SUCLA2, SMARCA1, PIK3R4, ACSL6
GOTERM_MF_ALL	GO:0000166~nucleotide binding	14	14	0,480	GIMAP5, PDK4, MLH1, SPEN, DGKI, SAFB2, TPK1, MAP3K3, SPEG, SUCLA2, SMARCA1, RBM15, PIK3R4, ACSL6
GOTERM_MF_FAT	GO:0005524~ATP binding	10	10	0,486	TPK1, MAP3K3, SPEG, PDK4, MLH1, DGKI, SUCLA2, SMARCA1, PIK3R4, ACSL6
GOTERM_MF_FAT	GO:0032559~adenyl ribonucleotide binding	10	10	0,503	TPK1, MAP3K3, SPEG, PDK4, MLH1, DGKI, SUCLA2, SMARCA1, PIK3R4, ACSL6
GOTERM_MF_ALL	GO:0030554~adenyl nucleotide binding	10	10	0,517	TPK1, MAP3K3, SPEG, PDK4, MLH1, DGKI, SUCLA2, SMARCA1, PIK3R4, ACSL6
GOTERM_MF_ALL	GO:0001883~purine nucleoside binding	10	10	0,535	TPK1, MAP3K3, SPEG, PDK4, MLH1, DGKI, SUCLA2, SMARCA1, PIK3R4, ACSL6
GOTERM_MF_FAT	GO:0000166~nucleotide binding	14	14	0,544	GIMAP5, PDK4, MLH1, SPEN, DGKI, SAFB2, TPK1, MAP3K3, SPEG, SUCLA2, SMARCA1, RBM15, PIK3R4, ACSL6
GOTERM_MF_ALL	GO:0001882~nucleoside binding	10	10	0,544	TPK1, MAP3K3, SPEG, PDK4, MLH1, DGKI, SUCLA2, SMARCA1, PIK3R4, ACSL6
GOTERM_MF_FAT	GO:0030554~adenyl nucleotide binding	10	10	0,569	TPK1, MAP3K3, SPEG, PDK4, MLH1, DGKI, SUCLA2, SMARCA1, PIK3R4, ACSL6
GOTERM_MF_ALL	GO:0032555~purine ribonucleotide binding	11	11	0,575	TPK1, GIMAP5, MAP3K3, SPEG, PDK4, MLH1, DGKI, SUCLA2, SMARCA1, PIK3R4, ACSL6
GOTERM_MF_ALL	GO:0032553~ribonucleotide binding	11	11	0,575	TPK1, GIMAP5, MAP3K3, SPEG, PDK4, MLH1, DGKI, SUCLA2, SMARCA1, PIK3R4, ACSL6
GOTERM_MF_FAT	GO:0001883~purine nucleoside binding	10	10	0,588	TPK1, MAP3K3, SPEG, PDK4, MLH1, DGKI, SUCLA2, SMARCA1, PIK3R4, ACSL6
GOTERM_MF_FAT	GO:0001882~nucleoside binding	10	10	0,596	TPK1, MAP3K3, SPEG, PDK4, MLH1, DGKI, SUCLA2, SMARCA1, PIK3R4, ACSL6
GOTERM_MF_FAT	GO:0032555~purine ribonucleotide binding	11	11	0,630	TPK1, GIMAP5, MAP3K3, SPEG, PDK4, MLH1, DGKI, SUCLA2, SMARCA1, PIK3R4, ACSL6
GOTERM_MF_FAT	GO:0032553~ribonucleotide binding	11	11	0,630	TPK1, GIMAP5, MAP3K3, SPEG, PDK4, MLH1, DGKI, SUCLA2, SMARCA1, PIK3R4, ACSL6
GOTERM_MF_ALL	GO:0017076~purine nucleotide binding	11	11	0,633	TPK1, GIMAP5, MAP3K3, SPEG, PDK4, MLH1, DGKI, SUCLA2, SMARCA1, PIK3R4, ACSL6
GOTERM_MF_FAT	GO:0017076~purine nucleotide binding	11	11	0,687	TPK1, GIMAP5, MAP3K3, SPEG, PDK4, MLH1, DGKI, SUCLA2, SMARCA1, PIK3R4, ACSL6

Annotation Cluster 83	Localization and transport	Enrichment Score: 0,2680143662186858			
Category	Term	Count	%	PValue	Genes
GOTERM_BP_ALL	GO:0051179~localization	19	20	0,477	PPARA, CAV2, IL6, GIMAP5, NRXN3, PLXNA2, NLGN1, GOLIM4, MLH1, MITD1, ARMC1, DVL1, ANK3, SLC24A1, SLC6A6, GRID2, GAA, PITPNC1, NEFL
GOTERM_BP_ALL	GO:0051234~establishment of localization	17	18	0,480	CAV2, PPARA, IL6, GIMAP5, NRXN3, NLGN1, GOLIM4, MLH1, MITD1, ARMC1, DVL1, ANK3, SLC24A1, SLC6A6, GRID2, PITPNC1, NEFL
GOTERM_BP_ALL	GO:0006810~transport	15	15	0,685	CAV2, PPARA, IL6, GIMAP5, NRXN3, NLGN1, GOLIM4, MITD1, ARMC1, DVL1, SLC6A6, SLC24A1, GRID2, PITPNC1, NEFL

Annotation Cluster 84	Vesicle	Enrichment Score: 0,2539498140298554			
Category	Term	Count	%	PValue	Genes
GOTERM_CC_ALL	GO:0031410~cytoplasmic vesicle	5	5	0,477	MIB1, CAV2, GOLIM4, FASN, DVL1
GOTERM_CC_FAT	GO:0031410~cytoplasmic vesicle	5	5	0,503	MIB1, CAV2, GOLIM4, FASN, DVL1
GOTERM_CC_ALL	GO:0031982~vesicle	5	5	0,511	MIB1, CAV2, GOLIM4, FASN, DVL1
GOTERM_CC_FAT	GO:0031982~vesicle	5	5	0,537	MIB1, CAV2, GOLIM4, FASN, DVL1
GOTERM_CC_ALL	GO:0016023~cytoplasmic membrane-bounded vesicle	4	4	0,591	CAV2, GOLIM4, FASN, DVL1
GOTERM_CC_ALL	GO:0031988~membrane-bounded vesicle	4	4	0,613	CAV2, GOLIM4, FASN, DVL1
GOTERM_CC_FAT	GO:0016023~cytoplasmic membrane-bounded vesicle	4	4	0,614	CAV2, GOLIM4, FASN, DVL1
GOTERM_CC_FAT	GO:0031988~membrane-bounded vesicle	4	4	0,636	CAV2, GOLIM4, FASN, DVL1

Annotation Cluster 85	Cellular response to stress and DNA damage stimulus	Enrichment Score: 0,24427402312472687			
Category	Term	Count	%	PValue	Genes
SP_PIR_KEYWORDS	DNA damage	3	3	0,273	RIF1, MLH1, RRM2B
GOTERM_BP_ALL	GO:0006974~response to DNA damage stimulus	3	3	0,642	RIF1, MLH1, RRM2B
GOTERM_BP_ALL	GO:0033554~cellular response to stress	4	4	0,644	RIF1, MLH1, RRM2B, NEFL
GOTERM_BP_FAT	GO:0006974~response to DNA damage stimulus	3	3	0,652	RIF1, MLH1, RRM2B
GOTERM_BP_FAT	GO:0033554~cellular response to stress	4	4	0,656	RIF1, MLH1, RRM2B, NEFL
GOTERM_BP_ALL	GO:0051716~cellular response to stimulus	5	5	0,709	IL6, RIF1, MLH1, RRM2B, NEFL

Annotation Cluster 86	Response to stimulus	Enrichment Score: 0,23860959171252186			
Category	Term	Count	%	PValue	Genes
GOTERM_BP_ALL	GO:0050896~response to stimulus	7	7	0,439	IL6, CASR, BLB1, SLC24A1, DNAJC3, NRG1, CALB1
UP_SEQ_FEATURE	signal peptide	4	4	0,640	IL6, BLB1, SLC24A1, DNAJC3
SP_PIR_KEYWORDS	signal	4	4	0,685	IL6, BLB1, SLC24A1, DNAJC3

Annotation Cluster 87	Protein modification process	Enrichment Score: 0,23801960042497033			
Category	Term	Count	%	PValue	Genes
GOTERM_BP_ALL	GO:0006464~protein modification process	10	10	0,472	ST3GAL1, MIB1, CAV2, PPP2R3A, PTP4A3, ST3GAL5, MAP3K3, SPEG, PDK4, PIK3R4
GOTERM_BP_ALL	GO:0043412~biopolymer modification	10	10	0,533	ST3GAL1, MIB1, CAV2, PPP2R3A, PTP4A3, ST3GAL5, MAP3K3, SPEG, PDK4, PIK3R4
GOTERM_BP_ALL	GO:0043687~post-translational protein modification	8	8	0,536	MIB1, CAV2, PPP2R3A, PTP4A3, MAP3K3, SPEG, PDK4, PIK3R4
GOTERM_BP_ALL	GO:0044267~cellular protein metabolic process	12	12	0,827	ST3GAL1, MIB1, CAV2, PPP2R3A, PTP4A3, DIO2, ST3GAL5, MAP3K3, SPEG, PDK4, PIK3R4, USP44

Annotation Cluster 88	Protein activity	Enrichment Score: 0,23510432076531398			
Category	Term	Count	%	PValue	Genes
GOTERM_MF_ALL	GO:0042803~protein homodimerization activity	3	3	0,556	CAV2, OLFML2A, FOXP1
GOTERM_MF_FAT	GO:0042803~protein homodimerization activity	3	3	0,579	CAV2, OLFML2A, FOXP1
GOTERM_MF_ALL	GO:0046983~protein dimerization activity	4	4	0,583	BACH1, CAV2, OLFML2A, FOXP1
GOTERM_MF_FAT	GO:0046983~protein dimerization activity	4	4	0,611	BACH1, CAV2, OLFML2A, FOXP1

Annotation Cluster 89	Embryonic development	Enrichment Score: 0,23365745528457882			
Category	Term	Count	%	PValue	Genes
GOTERM_BP_ALL	GO:0043009~chordate embryonic development	3	3	0,577	MIB1, LIMS1, DVL1
GOTERM_BP_ALL	GO:0009792~embryonic development ending in birth or egg hatching	3	3	0,581	MIB1, LIMS1, DVL1
GOTERM_BP_FAT	GO:0043009~chordate embryonic development	3	3	0,586	MIB1, LIMS1, DVL1
GOTERM_BP_FAT	GO:0009792~embryonic development ending in birth or egg hatching	3	3	0,591	MIB1, LIMS1, DVL1

Annotation Cluster 90	Metal ion binding	Enrichment Score: 0,2099449928862685			
Category	Term	Count	%	PValue	Genes
GOTERM_MF_ALL	GO:0008270~zinc ion binding	14	14	0,525	PPARA, LIMS1, GDA, ADAMTSL1, KLF9, MMEL1, SMYD3, DGKI, FOXP1, NEBL, MIB1, FASN, ADAMTS5, USP44
SP_PIR_KEYWORDS	zinc	12	12	0,538	MIB1, PPARA, GDA, LIMS1, KLF9, MMEL1, SMYD3, DGKI, ADAMTS5, FOXP1, USP44, NEBL
GOTERM_MF_FAT	GO:0008270~zinc ion binding	14	14	0,590	PPARA, LIMS1, GDA, ADAMTSL1, KLF9, MMEL1, SMYD3, DGKI, FOXP1, NEBL, MIB1, FASN, ADAMTS5, USP44
GOTERM_MF_ALL	GO:0046914~transition metal ion binding	15	15	0,702	PPARA, LIMS1, GDA, ADAMTSL1, KLF9, MMEL1, SMYD3, RRM2B, DGKI, FOXP1, NEBL, MIB1, FASN, ADAMTS5, USP44
GOTERM_MF_FAT	GO:0046914~transition metal ion binding	15	15	0,763	PPARA, LIMS1, GDA, ADAMTSL1, KLF9, MMEL1, SMYD3, RRM2B, DGKI, FOXP1, NEBL, MIB1, FASN, ADAMTS5, USP44

Annotation Cluster 91	Protein metabolism	Enrichment Score: 0,18230711470278063			
Category	Term	Count	%	PValue	Genes
UP_SEQ_FEATURE	binding site:ATP	4	4	0,515	MAP3K3, SPEG, PDK4, PIK3R4
INTERPRO	IPR008271:Serine/threonine protein kinase, active site	3	3	0,561	MAP3K3, SPEG, PIK3R4
SP_PIR_KEYWORDS	serine/threonine-protein kinase	3	3	0,570	MAP3K3, SPEG, PIK3R4
GOTERM_MF_ALL	GO:0004674~protein serine/threonine kinase activity	3	3	0,694	MAP3K3, SPEG, PIK3R4
INTERPRO	IPR017441:Protein kinase, ATP binding site	3	3	0,697	MAP3K3, SPEG, PIK3R4
GOTERM_MF_FAT	GO:0004674~protein serine/threonine kinase activity	3	3	0,715	MAP3K3, SPEG, PIK3R4
INTERPRO	IPR000719:Protein kinase, core	3	3	0,721	MAP3K3, SPEG, PIK3R4
UP_SEQ_FEATURE	active site:Proton acceptor	3	3	0,848	MAP3K3, SPEG, PIK3R4

Annotation Cluster 92	Cellular homeostasis	Enrichment Score: 0,18135796908293353			
Category	Term	Count	%	PValue	Genes
GOTERM_BP_ALL	GO:0019725~cellular homeostasis	4	4	0,511	CASR, GIMAP5, GAA, GRID2
GOTERM_BP_FAT	GO:0019725~cellular homeostasis	4	4	0,524	CASR, GIMAP5, GAA, GRID2
GOTERM_BP_ALL	GO:0006873~cellular ion homeostasis	3	3	0,643	CASR, GIMAP5, GRID2
GOTERM_BP_ALL	GO:0055082~cellular chemical homeostasis	3	3	0,652	CASR, GIMAP5, GRID2
GOTERM_BP_FAT	GO:0006873~cellular ion homeostasis	3	3	0,653	CASR, GIMAP5, GRID2

GOTERM_BP_FAT	GO:0055082~cellular chemical homeostasis	3	3	0,662	CASR, GIMAP5, GRID2
GOTERM_BP_ALL	GO:0050801~ion homeostasis	3	3	0,692	CASR, GIMAP5, GRID2
GOTERM_BP_FAT	GO:0050801~ion homeostasis	3	3	0,701	CASR, GIMAP5, GRID2
GOTERM_BP_ALL	GO:0048878~chemical homeostasis	3	3	0,803	CASR, GIMAP5, GRID2
GOTERM_BP_FAT	GO:0048878~chemical homeostasis	3	3	0,812	CASR, GIMAP5, GRID2

**Annotation Cluster 93**	**Oxidoreductase activity**	**Enrichment Score: 0,16868258938509684**			
Category	Term	Count	%	PValue	Genes
SP_PIR_KEYWORDS	oxidoreductase	4	4	0,535	DIO2, DIO3, FASN, RRM2B
GOTERM_BP_ALL	GO:0055114~oxidation reduction	4	4	0,724	DIO2, DIO3, FASN, RRM2B
GOTERM_BP_FAT	GO:0055114~oxidation reduction	4	4	0,736	DIO2, DIO3, FASN, RRM2B
GOTERM_MF_ALL	GO:0016491~oxidoreductase activity	4	4	0,742	DIO2, DIO3, FASN, RRM2B

**Annotation Cluster 94**	**Metabolic process**	**Enrichment Score: 0,16558065363104665**			
Category	Term	Count	%	PValue	Genes
GOTERM_BP_ALL	GO:0044237~cellular metabolic process	39	40	0,591	BACH1, CAV2, PPARA, GDA, E2F3, PPP2R3A, UPP2, AHCTF1, MLH1, EHF, RRM2B, TPK1, ST3GAL1, MAP3K3, ST3GAL5, SPEG, FASN, BCL9L, SUCLA2, PLCB1, PIK3R4, ACSL6, ERF, KLF9, PDK4, SPEN, FOXP1, SAFB2, DVL1, MIB1, DIO2, NCOA3, PTP4A3, SLC6A6, GAA, VOPP1, SMARCA1, ADC, USP44
GOTERM_BP_ALL	GO:0044238~primary metabolic process	39	40	0,726	BACH1, CAV2, PPARA, GDA, E2F3, PPP2R3A, UPP2, AHCTF1, MLH1, EHF, RRM2B, ST3GAL1, MAP3K3, ST3GAL5, SPEG, FASN, ITIH5, BCL9L, PLCB1, PIK3R4, ACSL6, ERF, KLF9, MMEL1, PDK4, SPEN, FOXP1, SAFB2, MIB1, DIO2, NCOA3, PTP4A3, SLC6A6, GAA, VOPP1, SMARCA1, ADC, ADAMTS5, USP44
GOTERM_BP_ALL	GO:0008152~metabolic process	43	44	0,742	BACH1, CAV2, PPARA, GDA, E2F3, PPP2R3A, UPP2, AHCTF1, MLH1, EHF, RRM2B, TPK1, ST3GAL1, MAP3K3, ST3GAL5, SPEG, FASN, ITIH5, BCL9L, SUCLA2, PLCB1, PIK3R4, ACSL6, ERF, KLF9, MMEL1, PDK4, SPEN, FOXP1, SAFB2, DVL1, MIB1, DIO2, NCOA3, PTP4A3, DIO3, SLC6A6, GAA, VOPP1, SMARCA1, ADC, ADAMTS5, USP44

**Annotation Cluster 95**	**DNA binding**	**Enrichment Score: 0,14526789820070427**			
Category	Term	Count	%	PValue	Genes

SP_PIR_KEYWORDS	dna-binding	10	10	0,595	BACH1, ERF, PPARA, E2F3, KLF9, AHCTF1, EHF, SPEN, FOXP1, SAFB2
GOTERM_MF_ALL	GO:0003677~DNA binding	13	13	0,657	BACH1, PPARA, ERF, E2F3, KLF9, AHCTF1, MLH1, EHF, SPEN, GRAMD4, FOXP1, SAFB2, SMARCA1
GOTERM_MF_FAT	GO:0003677~DNA binding	13	13	0,716	BACH1, PPARA, ERF, E2F3, KLF9, AHCTF1, MLH1, EHF, SPEN, GRAMD4, FOXP1, SAFB2, SMARCA1
GOTERM_MF_ALL	GO:0003676~nucleic acid binding	14	14	0,937	BACH1, PPARA, ERF, E2F3, KLF9, MLH1, AHCTF1, EHF, SPEN, GRAMD4, FOXP1, SAFB2, SMARCA1, RBM15

**Annotation Cluster 96**	**Reproductive process**	**Enrichment Score: 0,09760992336207024**			
Category	Term	Count	%	PValue	Genes
GOTERM_BP_ALL	GO:0048609~reproductive process in a multicellular organism	3	3	0,780	CAV2, MLH1, ADC
GOTERM_BP_ALL	GO:0032504~multicellular organism reproduction	3	3	0,780	CAV2, MLH1, ADC
GOTERM_BP_FAT	GO:0048609~reproductive process in a multicellular organism	3	3	0,789	CAV2, MLH1, ADC
GOTERM_BP_FAT	GO:0032504~multicellular organism reproduction	3	3	0,789	CAV2, MLH1, ADC
GOTERM_BP_ALL	GO:0022414~reproductive process	4	4	0,827	CAV2, KLF9, MLH1, ADC
GOTERM_BP_ALL	GO:0000003~reproduction	4	4	0,830	CAV2, KLF9, MLH1, ADC

**Annotation Cluster 97**	**Proteolysis**	**Enrichment Score: 0,06589792400604289**			
Category	Term	Count	%	PValue	Genes
SP_PIR_KEYWORDS	Protease	3	3	0,700	MMEL1, ADAMTS5, USP44
GOTERM_BP_ALL	GO:0006508~proteolysis	4	4	0,950	MIB1, MMEL1, ADAMTS5, USP44
GOTERM_BP_FAT	GO:0006508~proteolysis	4	4	0,954	MIB1, MMEL1, ADAMTS5, USP44

**Annotation Cluster 98**	**Catabolic process**	**Enrichment Score: 0,05248272275065522**			
Category	Term	Count	%	PValue	Genes
GOTERM_BP_ALL	GO:0009057~macromolecule catabolic process	4	4	0,839	MIB1, GAA, MLH1, USP44
GOTERM_BP_FAT	GO:0009057~macromolecule catabolic process	4	4	0,848	MIB1, GAA, MLH1, USP44
GOTERM_BP_ALL	GO:0044265~cellular macromolecule catabolic process	3	3	0,928	MIB1, MLH1, USP44

GOTERM_BP_FAT	GO:0044265~cellular macromolecule catabolic process	3	3	0,933	MIB1, MLH1, USP44

**Annotation Cluster 99**	**Apoptosis**	**Enrichment Score: 0,044554896895792985**			
Category	Term	Count	%	PValue	Genes
GOTERM_BP_ALL	GO:0006915~apoptosis	3	3	0,870	TNFRSF1B, IL6, GRAMD4
GOTERM_BP_ALL	GO:0012501~programmed cell death	3	3	0,876	TNFRSF1B, IL6, GRAMD4
GOTERM_BP_FAT	GO:0006915~apoptosis	3	3	0,877	TNFRSF1B, IL6, GRAMD4
GOTERM_BP_FAT	GO:0012501~programmed cell death	3	3	0,882	TNFRSF1B, IL6, GRAMD4
GOTERM_BP_ALL	GO:0008219~cell death	3	3	0,926	TNFRSF1B, IL6, GRAMD4
GOTERM_BP_ALL	GO:0016265~death	3	3	0,928	TNFRSF1B, IL6, GRAMD4
GOTERM_BP_FAT	GO:0008219~cell death	3	3	0,931	TNFRSF1B, IL6, GRAMD4
GOTERM_BP_FAT	GO:0016265~death	3	3	0,933	TNFRSF1B, IL6, GRAMD4

**Annotation Cluster 100**	**Protein localization**	**Enrichment Score: 0,035904967616528**			
Category	Term	Count	%	PValue	Genes
GOTERM_BP_ALL	GO:0008104~protein localization	4	4	0,894	ANK3, NLGN1, MITD1, DVL1
GOTERM_BP_FAT	GO:0008104~protein localization	4	4	0,901	ANK3, NLGN1, MITD1, DVL1
GOTERM_BP_ALL	GO:0045184~establishment of protein localization	3	3	0,943	ANK3, NLGN1, MITD1
GOTERM_BP_FAT	GO:0045184~establishment of protein localization	3	3	0,947	ANK3, NLGN1, MITD1

**Annotation Cluster 101**	**Transmembrane transporter activity**	**Enrichment Score: 0,025639292457593648**			
Category	Term	Count	%	PValue	Genes
GOTERM_MF_ALL	GO:0015075~ion transmembrane transporter activity	3	3	0,916	SLC24A1, SLC6A6, GRID2
GOTERM_MF_ALL	GO:0022891~substrate-specific transmembrane transporter activity	3	3	0,948	SLC24A1, SLC6A6, GRID2
GOTERM_MF_ALL	GO:0022857~transmembrane transporter activity	3	3	0,965	SLC24A1, SLC6A6, GRID2

**Annotation Cluster 102**	**Organelle**	**Enrichment Score: 0,02135582187251642**			
Category	Term	Count	%	PValue	Genes
GOTERM_CC_ALL	GO:0070013~intracellular organelle lumen	7	7	0,938	BACH1, E2F3, PDK4, GOLIM4, AHCTF1, SPEN, FOXP1
GOTERM_CC_ALL	GO:0043233~organelle lumen	7	7	0,947	BACH1, E2F3, PDK4, GOLIM4, AHCTF1, SPEN, FOXP1
GOTERM_CC_FAT	GO:0070013~intracellular organelle lumen	7	7	0,952	BACH1, E2F3, PDK4, GOLIM4, AHCTF1, SPEN, FOXP1

GOTERM_CC_ALL	GO:0031974~membrane-enclosed lumen	7	7	0,953	BACH1, E2F3, PDK4, GOLIM4, AHCTF1, SPEN, FOXP1
GOTERM_CC_FAT	GO:0043233~organelle lumen	7	7	0,959	BACH1, E2F3, PDK4, GOLIM4, AHCTF1, SPEN, FOXP1
GOTERM_CC_FAT	GO:0031974~membrane-enclosed lumen	7	7	0,964	BACH1, E2F3, PDK4, GOLIM4, AHCTF1, SPEN, FOXP1

Functional Annotation of Up-regulated Genes

Annotation Cluster 1

List Total	Pop Hits	Pop Total	Fold Enrichment	Bonferroni	Benjamini	FDR
83	2330	14116	2,12	0,09	0,09	0,12
83	2527	14116	2,02	0,14	0,07	0,19
83	2865	14116	1,90	0,21	0,08	0,31
83	3148	14116	1,78	0,44	0,08	0,74

Annotation Cluster 2

List Total	Pop Hits	Pop Total	Fold Enrichment	Bonferroni	Benjamini	FDR
83	211	14116	6,45	0,25	0,07	0,36
81	211	13528	6,33	0,24	0,24	0,39
83	119	14116	8,58	0,55	0,08	1,03
81	119	13528	8,42	0,54	0,23	1,10
83	125	14116	8,16	0,63	0,08	1,29
81	125	13528	8,02	0,62	0,22	1,37

Annotation Cluster 3

List Total	Pop Hits	Pop Total	Fold Enrichment	Bonferroni	Benjamini	FDR
83	559	14116	3,65	0,38	0,08	0,60

83	601	14116	3,40	0,58	0,08	1,10
83	1088	14116	2,34	0,99	0,23	5,94

Annotation Cluster 4

List Total	Pop Hits	Pop Total	Fold Enrichment	Bonferroni	Benjamini	FDR
83	66	14116	10,31	1,00	0,32	10,28
83	66	14116	10,31	1,00	0,32	10,28
81	66	13528	10,12	1,00	0,59	10,63
81	66	13528	10,12	1,00	0,59	10,63

Annotation Cluster 5

List Total	Pop Hits	Pop Total	Fold Enrichment	Bonferroni	Benjamini	FDR
83	121	14116	7,03	1,00	0,28	8,39
81	121	13528	6,90	1,00	0,56	8,79
83	88	14116	7,73	1,00	0,40	21,22
81	88	13528	7,59	1,00	0,58	21,87

Annotation Cluster 6

List Total	Pop Hits	Pop Total	Fold Enrichment	Bonferroni	Benjamini	FDR
83	162	14116	7,35	0,36	0,09	0,58
81	162	13528	7,22	0,36	0,20	0,62
83	505	14116	3,03	1,00	0,35	13,56
83	505	14116	3,03	1,00	0,35	13,56
81	505	13528	2,98	1,00	0,61	14,66

81	505	13528	2,98	1,00	0,61	14,66
83	318	14116	3,74	1,00	0,39	15,68
81	318	13528	3,68	1,00	0,63	16,66
83	357	14116	3,33	1,00	0,41	25,06
81	357	13528	3,27	1,00	0,58	26,47
83	887	14116	2,11	1,00	0,55	41,40
83	665	14116	2,30	1,00	0,59	47,59
81	665	13528	2,26	1,00	0,77	50,14
83	710	14116	2,16	1,00	0,64	59,17
81	710	13528	2,12	1,00	0,79	61,79
83	1001	14116	1,87	1,00	0,67	65,87

Annotation Cluster 7

List Total	Pop Hits	Pop Total	Fold Enrichment	Bonferroni	Benjamini	FDR
83	256	14116	4,65	0,99	0,24	5,89
81	256	13528	4,57	0,99	0,54	6,30
83	209	14116	4,88	1,00	0,33	11,33
81	209	13528	4,79	1,00	0,59	11,97
83	213	14116	4,79	1,00	0,34	12,19
81	213	13528	4,70	1,00	0,59	12,87

83	438	14116	3,11	1,00	0,42	19,99
83	244	14116	4,18	1,00	0,41	20,18
83	339	14116	3,51	1,00	0,41	20,43
83	245	14116	4,17	1,00	0,40	20,47
81	244	13528	4,11	1,00	0,65	21,20
81	438	13528	3,05	1,00	0,63	21,35
81	245	13528	4,09	1,00	0,61	21,51
81	339	13528	3,45	1,00	0,60	21,64
83	256	14116	3,99	1,00	0,40	23,85
83	356	14116	3,34	1,00	0,41	24,79
81	256	13528	3,91	1,00	0,59	25,01
81	356	13528	3,28	1,00	0,59	26,19
83	368	14116	3,24	1,00	0,43	28,11
81	368	13528	3,18	1,00	0,62	29,64
83	193	14116	4,41	1,00	0,49	34,79
81	193	13528	4,33	1,00	0,66	36,02
83	397	14116	3,00	1,00	0,51	36,84
81	397	13528	2,94	1,00	0,68	38,65

Annotation Cluster 8

List Total	Pop Hits	Pop Total	Fold Enrichment	Bonferroni	Benjamini	FDR
83	61	14116	11,15	1,00	0,29	8,34
81	61	13528	10,95	1,00	0,60	8,63
83	83	14116	8,20	1,00	0,41	18,43
81	83	13528	8,05	1,00	0,66	19,00
83	85	14116	8,00	1,00	0,42	19,53
81	85	13528	7,86	1,00	0,65	20,13
83	163	14116	5,22	1,00	0,39	21,72

81	163	13528	5,12	1,00	0,58	22,60
83	34	14116	15,01	1,00	0,41	23,82
81	34	13528	14,74	1,00	0,59	24,25
83	67	14116	7,62	1,00	0,65	62,07
81	67	13528	7,48	1,00	0,79	62,77
83	207	14116	3,29	1,00	0,80	87,34
81	207	13528	3,23	1,00	0,90	88,06
83	300	14116	2,27	1,00	0,92	99,13
81	300	13528	2,23	1,00	0,97	99,23

Annotation Cluster 9

List Total	Pop Hits	Pop Total	Fold Enrichment	Bonferroni	Benjamini	FDR
89	8977	15908	1,21	0,96	0,96	18,30
89	8989	15908	1,21	0,97	0,68	18,89

89	7982	15908	1,21	1,00	0,72	37,63
89	7989	15908	1,21	1,00	0,67	38,18
89	10624	15908	1,13	1,00	0,76	54,56

Annotation Cluster 10

List Total	Pop Hits	Pop Total	Fold Enrichment	Bonferroni	Benjamini	FDR
89	1215	15908	1,91	1,00	0,76	36,09
74	1215	12782	1,85	1,00	0,98	40,86
89	1188	15908	1,81	1,00	0,73	55,70
74	1188	12782	1,74	1,00	0,97	61,02

Annotation Cluster 11

List Total	Pop Hits	Pop Total	Fold Enrichment	Bonferroni	Benjamini	FDR
97	130	19113	6,06	1,00	1,00	34,26
97	131	19113	6,02	1,00	0,99	34,81
89	184	16659	4,07	1,00	1,00	63,76
89	190	16659	3,94	1,00	1,00	66,62
56	190	9079	3,41	1,00	0,98	69,32

Annotation Cluster 12

List Total	Pop Hits	Pop Total	Fold Enrichment	Bonferroni	Benjamini	FDR
83	50	14116	10,20	1,00	0,56	43,16
81	50	13528	10,02	1,00	0,72	43,79
83	159	14116	4,28	1,00	0,67	66,64
81	159	13528	4,20	1,00	0,81	67,74
83	100	14116	5,10	1,00	0,80	86,25
81	100	13528	5,01	1,00	0,90	86,73

Annotation Cluster 13

List Total	Pop Hits	Pop Total	Fold Enrichment	Bonferroni	Benjamini	FDR
83	561	14116	2,43	1,00	0,60	52,14
81	561	13528	2,38	1,00	0,76	54,49
83	573	14116	2,37	1,00	0,62	55,58
81	573	13528	2,33	1,00	0,77	57,95
83	720	14116	2,13	1,00	0,65	61,68

83	780	14116	1,96	1,00	0,74	75,44
83	547	14116	2,18	1,00	0,77	81,40
81	547	13528	2,14	1,00	0,89	83,02
83	734	14116	1,85	1,00	0,82	90,35
81	734	13528	1,82	1,00	0,91	91,58

Annotation Cluster 14

List Total	Pop Hits	Pop Total	Fold Enrichment	Bonferroni	Benjamini	FDR
83	133	14116	5,11	1,00	0,61	50,48
81	133	13528	5,02	1,00	0,77	51,58
83	166	14116	4,10	1,00	0,71	70,48
81	166	13528	4,02	1,00	0,84	71,55
83	192	14116	3,54	1,00	0,77	82,29
81	192	13528	3,48	1,00	0,88	83,16
83	205	14116	3,32	1,00	0,80	86,74
81	205	13528	3,26	1,00	0,90	87,48

Annotation Cluster 15

List Total	Pop Hits	Pop Total	Fold Enrichment	Bonferroni	Benjamini	FDR
83	49	14116	10,41	1,00	0,55	41,95
81	49	13528	10,23	1,00	0,72	42,58
83	147	14116	4,63	1,00	0,63	59,51
81	147	13528	4,54	1,00	0,79	60,63
83	253	14116	2,69	1,00	0,88	96,20

81	253	13528	2,64	1,00	0,94	96,51
83	436	14116	1,95	1,00	0,92	99,02
81	436	13528	1,92	1,00	0,97	99,16

Annotation Cluster 16

List Total	Pop Hits	Pop Total	Fold Enrichment	Bonferroni	Benjamini	FDR
83	720	14116	2,13	1,00	0,65	61,68
83	519	14116	2,29	1,00	0,74	74,72
83	780	14116	1,96	1,00	0,74	75,44
81	519	13528	2,25	1,00	0,87	76,59
83	512	14116	1,99	1,00	0,88	95,78
81	512	13528	1,96	1,00	0,94	96,31

Annotation Cluster 17

List Total	Pop Hits	Pop Total	Fold Enrichment	Bonferroni	Benjamini	FDR
83	7106	14116	1,17	1,00	0,74	75,96
83	6819	14116	1,17	1,00	0,77	80,63

83	7484	14116	1,14	1,00	0,82	90,05

Annotation Cluster 18

List Total	Pop Hits	Pop Total	Fold Enrichment	Bonferroni	Benjamini	FDR
89	179	15908	3,99	1,00	0,77	63,43
74	179	12782	3,86	1,00	0,96	65,59
89	133	15908	4,03	1,00	0,83	90,03
74	133	12782	3,90	1,00	0,95	90,89

Annotation Cluster 19

List Total	Pop Hits	Pop Total	Fold Enrichment	Bonferroni	Benjamini	FDR
83	102	14116	5,00	1,00	0,80	87,18
81	102	13528	4,91	1,00	0,90	87,64
83	220	14116	3,09	1,00	0,82	90,75
81	220	13528	3,04	1,00	0,91	91,34

Annotation Cluster 20

List Total	Pop Hits	Pop Total	Fold Enrichment	Bonferroni	Benjamini	FDR
97	210	19235	3,78	1,00	0,78	68,28
89	213	16659	3,52	1,00	0,99	76,35
56	213	9079	3,04	1,00	0,93	78,64
97	177	19113	3,34	1,00	1,00	97,67

Annotation Cluster 21

List Total	Pop Hits	Pop Total	Fold Enrichment	Bonferroni	Benjamini	FDR
83	134	14116	5,08	1,00	0,61	51,15
81	134	13528	4,99	1,00	0,76	52,25

83	87	14116	5,86	1,00	0,76	78,81
81	87	13528	5,76	1,00	0,88	79,41
83	371	14116	1,38	1,00	0,99	100,00
81	371	13528	1,35	1,00	1,00	100,00

Annotation Cluster 22

List Total	Pop Hits	Pop Total	Fold Enrichment	Bonferroni	Benjamini	FDR
89	952	15908	1,88	1,00	0,76	64,88
74	952	12782	1,81	1,00	0,95	69,52
89	1381	15908	1,42	1,00	0,83	96,36
74	1381	12782	1,38	1,00	0,92	97,63

Annotation Cluster 23

List Total	Pop Hits	Pop Total	Fold Enrichment	Bonferroni	Benjamini	FDR
83	41	14116	12,44	1,00	0,47	32,22
81	41	13528	12,22	1,00	0,63	32,75
83	101	14116	5,05	1,00	0,81	86,72
81	101	13528	4,96	1,00	0,90	87,19
83	226	14116	2,26	1,00	0,95	99,96
83	385	14116	1,77	1,00	0,95	99,97

83	238	14116	2,14	1,00	0,96	99,98
81	238	13528	2,11	1,00	0,99	99,98

Annotation Cluster 24

List Total	Pop Hits	Pop Total	Fold Enrichment	Bonferroni	Benjamini	FDR
83	216	14116	3,15	1,00	0,82	89,79
81	216	13528	3,09	1,00	0,91	90,42
83	259	14116	2,63	1,00	0,88	96,81

Annotation Cluster 25

List Total	Pop Hits	Pop Total	Fold Enrichment	Bonferroni	Benjamini	FDR
83	295	14116	2,88	1,00	0,76	79,43
81	295	13528	2,83	1,00	0,88	80,66
83	329	14116	2,58	1,00	0,81	88,50
81	329	13528	2,54	1,00	0,90	89,39
83	878	14116	1,16	1,00	0,99	100,00

Annotation Cluster 26

List Total	Pop Hits	Pop Total	Fold Enrichment	Bonferroni	Benjamini	FDR
83	477	14116	2,50	1,00	0,65	62,70
83	481	14116	2,48	1,00	0,66	63,93
81	477	13528	2,45	1,00	0,80	64,78

81	481	13528	2,43	1,00	0,80	66,00
83	564	14116	2,11	1,00	0,79	84,85
81	564	13528	2,07	1,00	0,90	86,30
83	581	14116	2,05	1,00	0,81	87,83
81	581	13528	2,01	1,00	0,90	89,11
83	624	14116	1,91	1,00	0,85	93,47
81	624	13528	1,87	1,00	0,93	94,31
83	644	14116	1,85	1,00	0,87	95,27
83	654	14116	1,82	1,00	0,88	96,00
81	644	13528	1,82	1,00	0,94	95,93
81	654	13528	1,79	1,00	0,94	96,59
83	685	14116	1,74	1,00	0,90	97,71
83	695	14116	1,71	1,00	0,90	98,11
81	685	13528	1,71	1,00	0,96	98,09
81	695	13528	1,68	1,00	0,96	98,43
83	857	14116	1,39	1,00	0,95	99,96
81	857	13528	1,36	1,00	0,98	99,97

83	880	14116	1,35	1,00	0,96	99,98
83	921	14116	1,29	1,00	0,97	99,99

Annotation Cluster 27

List Total	Pop Hits	Pop Total	Fold Enrichment	Bonferroni	Benjamini	FDR
83	229	14116	3,71	1,00	0,61	52,13
81	229	13528	3,65	1,00	0,76	53,60
83	278	14116	3,06	1,00	0,73	73,51
81	278	13528	3,00	1,00	0,86	74,87
83	354	14116	1,92	1,00	0,95	99,88
83	359	14116	1,89	1,00	0,95	99,90
83	360	14116	1,89	1,00	0,95	99,91
81	354	13528	1,89	1,00	0,98	99,90
81	359	13528	1,86	1,00	0,98	99,92
81	360	13528	1,86	1,00	0,98	99,92

Annotation Cluster 28

List Total	Pop Hits	Pop Total	Fold Enrichment	Bonferroni	Benjamini	FDR
83	298	14116	2,85	1,00	0,77	80,38
81	298	13528	2,80	1,00	0,89	81,58
83	350	14116	2,43	1,00	0,83	92,35

81	350	13528	2,39	1,00	0,91	93,04
83	600	14116	1,70	1,00	0,93	99,39
81	600	13528	1,67	1,00	0,98	99,50
83	795	14116	1,50	1,00	0,94	99,79

Annotation Cluster 29

List Total	Pop Hits	Pop Total	Fold Enrichment	Bonferroni	Benjamini	FDR
83	138	14116	4,93	1,00	0,61	53,77
81	138	13528	4,84	1,00	0,76	54,89
83	381	14116	1,34	1,00	0,99	100,00
81	381	13528	1,32	1,00	1,00	100,00

Annotation Cluster 30

List Total	Pop Hits	Pop Total	Fold Enrichment	Bonferroni	Benjamini	FDR
89	809	15908	1,77	1,00	0,83	88,83
74	809	12782	1,71	1,00	0,94	91,15
89	839	15908	1,70	1,00	0,84	91,98
74	839	12782	1,65	1,00	0,95	93,84
89	1083	15908	1,49	1,00	0,84	97,28
74	1083	12782	1,44	1,00	0,93	98,17

Annotation Cluster 31

List Total	Pop Hits	Pop Total	Fold Enrichment	Bonferroni	Benjamini	FDR
89	205	16659	3,65	1,00	1,00	73,20
56	205	9079	3,16	1,00	0,96	75,65
97	470	19235	2,11	1,00	0,86	94,55
89	330	16659	2,27	1,00	1,00	97,92
89	501	16659	1,87	1,00	1,00	98,53
89	202	16659	2,78	1,00	1,00	98,92
56	330	9079	1,97	1,00	0,99	98,26

89	553	16659	1,69	1,00	1,00	99,55

Annotation Cluster 32

List Total	Pop Hits	Pop Total	Fold Enrichment	Bonferroni	Benjamini	FDR
83	556	14116	1,84	1,00	0,91	98,30
83	556	14116	1,84	1,00	0,91	98,30
83	560	14116	1,82	1,00	0,91	98,44
83	567	14116	1,80	1,00	0,91	98,67

Annotation Cluster 33

List Total	Pop Hits	Pop Total	Fold Enrichment	Bonferroni	Benjamini	FDR
89	155	15908	3,46	1,00	0,84	94,95
74	155	12782	3,34	1,00	0,94	95,50
89	172	15908	3,12	1,00	0,84	97,13
74	172	12782	3,01	1,00	0,93	97,49

Annotation Cluster 34

List Total	Pop Hits	Pop Total	Fold Enrichment	Bonferroni	Benjamini	FDR
83	115	14116	4,44	1,00	0,83	92,03
81	115	13528	4,36	1,00	0,91	92,38
83	123	14116	4,15	1,00	0,85	94,16
81	123	13528	4,07	1,00	0,93	94,44
83	319	14116	1,60	1,00	0,99	100,00
81	319	13528	1,57	1,00	1,00	100,00

Annotation Cluster 35

List Total	Pop Hits	Pop Total	Fold Enrichment	Bonferroni	Benjamini	FDR
97	803	19235	1,73	1,00	0,84	94,91
85	919	15143	1,55	1,00	1,00	97,88
76	919	12983	1,49	1,00	1,00	98,69

Annotation Cluster 36

List Total	Pop Hits	Pop Total	Fold Enrichment	Bonferroni	Benjamini	FDR
97	1689	19235	1,53	1,00	0,83	83,60
89	2010	15908	1,24	1,00	0,87	99,17
74	2010	12782	1,20	1,00	0,94	99,59

Annotation Cluster 37

List Total	Pop Hits	Pop Total	Fold Enrichment	Bonferroni	Benjamini	FDR
97	832	19235	1,67	1,00	0,86	96,51
89	1087	15908	1,48	1,00	0,83	97,42
74	1087	12782	1,43	1,00	0,92	98,27

Annotation Cluster 38

List Total	Pop Hits	Pop Total	Fold Enrichment	Bonferroni	Benjamini	FDR
83	530	14116	1,93	1,00	0,89	97,04
81	530	13528	1,89	1,00	0,95	97,45
83	914	14116	1,30	1,00	0,97	99,99

Annotation Cluster 39

List Total	Pop Hits	Pop Total	Fold Enrichment	Bonferroni	Benjamini	FDR
83	136	14116	3,75	1,00	0,88	96,56
81	136	13528	3,68	1,00	0,94	96,75
83	217	14116	2,35	1,00	0,95	99,93
81	217	13528	2,31	1,00	0,98	99,94

Annotation Cluster 40						
List Total	Pop Hits	Pop Total	Fold Enrichment	Bonferroni	Benjamini	FDR
83	153	14116	3,33	1,00	0,91	98,36
81	153	13528	3,27	1,00	0,96	98,46
83	195	14116	2,62	1,00	0,94	99,78
81	195	13528	2,57	1,00	0,98	99,80

Annotation Cluster 41						
List Total	Pop Hits	Pop Total	Fold Enrichment	Bonferroni	Benjamini	FDR
89	294	15908	2,43	1,00	0,83	95,75
74	294	12782	2,35	1,00	0,92	96,42
89	186	15908	2,88	1,00	0,85	98,24
74	186	12782	2,79	1,00	0,92	98,49
97	588	19235	1,69	1,00	0,89	99,42

Annotation Cluster 42						
List Total	Pop Hits	Pop Total	Fold Enrichment	Bonferroni	Benjamini	FDR
83	1503	14116	1,36	1,00	0,92	99,14
83	1210	14116	1,41	1,00	0,93	99,38
81	1210	13528	1,38	1,00	0,98	99,55

Annotation Cluster 43						
List Total	Pop Hits	Pop Total	Fold Enrichment	Bonferroni	Benjamini	FDR
85	1512	15143	1,41	1,00	1,00	96,19
83	1813	14116	1,31	1,00	0,92	99,07
76	1512	12983	1,36	1,00	1,00	97,88
81	1813	13528	1,29	1,00	0,97	99,37
83	1773	14116	1,25	1,00	0,95	99,86

81	1773	13528	1,22	1,00	0,98	99,91

Annotation Cluster 44

List Total	Pop Hits	Pop Total	Fold Enrichment	Bonferroni	Benjamini	FDR
83	109	14116	4,68	1,00	0,82	90,03
81	109	13528	4,60	1,00	0,90	90,43
83	290	14116	1,76	1,00	0,98	100,00
83	384	14116	1,33	1,00	0,99	100,00

Annotation Cluster 45

List Total	Pop Hits	Pop Total	Fold Enrichment	Bonferroni	Benjamini	FDR
25	717	3361	1,88	0,99	0,99	43,40
15	405	2121	2,09	1,00	1,00	71,10
25	787	3361	1,54	1,00	1,00	87,94
38	2563	5846	1,14	1,00	0,99	98,90
38	1826	5846	1,18	1,00	0,99	99,31
38	1461	5846	1,16	1,00	0,98	99,87
38	1514	5846	1,12	1,00	0,96	99,95
27	1461	3811	1,06	1,00	1,00	99,99
27	1514	3811	1,03	1,00	1,00	100,00

Annotation Cluster 46

List Total	Pop Hits	Pop Total	Fold Enrichment	Bonferroni	Benjamini	FDR
83	186	14116	2,74	1,00	0,94	99,65
83	186	14116	2,74	1,00	0,94	99,65
81	186	13528	2,69	1,00	0,98	99,68

81	186	13528	2,69	1,00	0,98	99,68

Annotation Cluster 47

List Total	Pop Hits	Pop Total	Fold Enrichment	Bonferroni	Benjamini	FDR
89	129	15908	4,16	1,00	0,84	88,79
74	129	12782	4,02	1,00	0,97	89,72
89	386	15908	1,85	1,00	0,90	99,64
74	386	12782	1,79	1,00	0,95	99,73
89	460	15908	1,55	1,00	0,94	99,97
74	460	12782	1,50	1,00	0,96	99,98

Annotation Cluster 48

List Total	Pop Hits	Pop Total	Fold Enrichment	Bonferroni	Benjamini	FDR
83	211	14116	3,22	1,00	0,81	88,48
81	211	13528	3,17	1,00	0,90	89,16
83	137	14116	3,72	1,00	0,88	96,70
81	137	13528	3,66	1,00	0,94	96,89
83	474	14116	1,08	1,00	1,00	100,00
81	474	13528	1,06	1,00	1,00	100,00
83	546	14116	0,93	1,00	1,00	100,00

Annotation Cluster 49

List Total	Pop Hits	Pop Total	Fold Enrichment	Bonferroni	Benjamini	FDR
97	3374	19113	1,46	1,00	0,98	48,57
97	4911	19113	1,16	1,00	1,00	98,63

97	4973	19235	1,16	1,00	0,87	97,74
89	5297	15908	1,08	1,00	0,91	99,79
74	5297	12782	1,04	1,00	0,97	99,96
89	5485	15908	1,04	1,00	0,93	99,97
74	5485	12782	1,01	1,00	0,97	100,00
89	6578	15908	0,98	1,00	0,96	100,00

Annotation Cluster 50

List Total	Pop Hits	Pop Total	Fold Enrichment	Bonferroni	Benjamini	FDR
83	512	14116	1,99	1,00	0,88	95,78

81	512	13528	1,96	1,00	0,94	96,31
83	459	14116	1,85	1,00	0,94	99,49
81	459	13528	1,82	1,00	0,98	99,57
83	504	14116	1,69	1,00	0,95	99,87
83	356	14116	1,91	1,00	0,95	99,89
81	504	13528	1,66	1,00	0,98	99,90
83	362	14116	1,88	1,00	0,95	99,91
81	356	13528	1,88	1,00	0,98	99,91
81	362	13528	1,85	1,00	0,98	99,93
83	266	14116	1,92	1,00	0,97	100,00
81	266	13528	1,88	1,00	0,99	100,00

Annotation Cluster 51

List Total	Pop Hits	Pop Total	Fold Enrichment	Bonferroni	Benjamini	FDR
83	586	14116	1,74	1,00	0,92	99,15
81	586	13528	1,71	1,00	0,97	99,29
83	969	14116	1,23	1,00	0,98	100,00

Annotation Cluster 52

List Total	Pop Hits	Pop Total	Fold Enrichment	Bonferroni	Benjamini	FDR

97	461	19235	2,58	1,00	0,84	65,12
83	776	14116	1,10	1,00	0,99	100,00
81	776	13528	1,08	1,00	1,00	100,00

Annotation Cluster 53

List Total	Pop Hits	Pop Total	Fold Enrichment	Bonferroni	Benjamini	FDR
38	71	5846	6,50	1,00	1,00	57,28
38	71	5846	6,50	1,00	1,00	57,28
27	71	3811	5,96	1,00	1,00	60,60
27	71	3811	5,96	1,00	1,00	60,60
38	1029	5846	1,50	1,00	1,00	90,50
38	272	5846	2,26	1,00	1,00	95,67
27	272	3811	2,08	1,00	1,00	97,12
38	1021	5846	1,36	1,00	1,00	98,45
38	386	5846	1,59	1,00	0,99	99,86
38	861	5846	1,25	1,00	0,98	99,91
27	386	3811	1,46	1,00	1,00	99,94
49	305	6610	1,33	1,00	1,00	100,00
61	305	8067	1,30	1,00	1,00	100,00
38	1066	5846	1,01	1,00	0,99	100,00
38	683	5846	0,90	1,00	1,00	100,00
38	683	5846	0,90	1,00	1,00	100,00
27	683	3811	0,83	1,00	1,00	100,00
27	683	3811	0,83	1,00	1,00	100,00

Annotation Cluster 54

List Total	Pop Hits	Pop Total	Fold Enrichment	Bonferroni	Benjamini	FDR
83	3464	14116	1,23	1,00	0,88	96,81
83	3621	14116	1,17	1,00	0,93	99,29
83	2839	14116	1,20	1,00	0,94	99,56
83	2856	14116	1,19	1,00	0,94	99,64
83	2947	14116	1,15	1,00	0,95	99,90
83	3291	14116	1,14	1,00	0,95	99,91
83	2966	14116	1,15	1,00	0,95	99,92
83	2814	14116	1,15	1,00	0,95	99,94
83	2830	14116	1,14	1,00	0,95	99,95
83	3259	14116	1,10	1,00	0,97	99,99
83	2601	14116	1,11	1,00	0,97	99,99

81	2601	13528	1,09	1,00	0,99	100,00

Annotation Cluster 55

List Total	Pop Hits	Pop Total	Fold Enrichment	Bonferroni	Benjamini	FDR
83	804	14116	1,48	1,00	0,95	99,83
83	812	14116	1,47	1,00	0,95	99,86
83	815	14116	1,46	1,00	0,95	99,87
81	804	13528	1,45	1,00	0,98	99,87
81	812	13528	1,44	1,00	0,98	99,90
81	815	13528	1,43	1,00	0,98	99,90

Annotation Cluster 56

List Total	Pop Hits	Pop Total	Fold Enrichment	Bonferroni	Benjamini	FDR
25	717	3361	1,88	0,99	0,99	43,40
15	405	2121	2,09	1,00	1,00	71,10
25	787	3361	1,54	1,00	1,00	87,94
15	233	2121	1,82	1,00	1,00	99,87
15	295	2121	1,44	1,00	1,00	99,99
15	410	2121	1,03	1,00	1,00	100,00
25	430	3361	0,94	1,00	1,00	100,00
22	1713	2963	0,63	1,00	1,00	100,00

Annotation Cluster 57

List Total	Pop Hits	Pop Total	Fold Enrichment	Bonferroni	Benjamini	FDR
97	688	19235	1,73	1,00	0,88	97,66
85	834	15143	1,50	1,00	1,00	99,43
85	721	15143	1,48	1,00	1,00	99,81

85	964	15143	1,29	1,00	1,00	99,97

Annotation Cluster 58

List Total	Pop Hits	Pop Total	Fold Enrichment	Bonferroni	Benjamini	FDR
83	114	14116	4,48	1,00	0,83	91,72
81	114	13528	4,40	1,00	0,91	92,08
83	466	14116	1,82	1,00	0,94	99,59
81	466	13528	1,79	1,00	0,98	99,65
83	485	14116	1,75	1,00	0,94	99,77
83	485	14116	1,75	1,00	0,94	99,77
81	485	13528	1,72	1,00	0,98	99,81
81	485	13528	1,72	1,00	0,98	99,81
83	345	14116	1,97	1,00	0,95	99,83
81	345	13528	1,94	1,00	0,98	99,86
83	357	14116	1,91	1,00	0,95	99,89
81	357	13528	1,87	1,00	0,98	99,91
83	372	14116	1,83	1,00	0,95	99,94
83	223	14116	2,29	1,00	0,95	99,95
81	372	13528	1,80	1,00	0,98	99,95
81	223	13528	2,25	1,00	0,98	99,95

83	231	14116	2,21	1,00	0,95	99,97
81	231	13528	2,17	1,00	0,98	99,97
83	240	14116	2,13	1,00	0,96	99,98
81	240	13528	2,09	1,00	0,99	99,98
83	520	14116	1,31	1,00	0,99	100,00
81	520	13528	1,28	1,00	1,00	100,00
83	846	14116	1,01	1,00	1,00	100,00

Annotation Cluster 59

List Total	Pop Hits	Pop Total	Fold Enrichment	Bonferroni	Benjamini	FDR
97	231	19113	2,56	1,00	1,00	99,70
97	232	19113	2,55	1,00	1,00	99,71
97	240	19235	2,48	1,00	0,89	99,41
89	238	16659	2,36	1,00	1,00	99,72
56	238	9079	2,04	1,00	0,99	99,68

Annotation Cluster 60

List Total	Pop Hits	Pop Total	Fold Enrichment	Bonferroni	Benjamini	FDR
97	135	19113	5,84	1,00	0,98	37,03
89	211	16659	2,66	1,00	1,00	99,22
89	213	16659	2,64	1,00	1,00	99,28
56	211	9079	2,31	1,00	0,99	99,13
97	540	19235	1,10	1,00	0,99	100,00
85	718	15143	0,74	1,00	1,00	100,00
76	718	12983	0,71	1,00	1,00	100,00

Annotation Cluster 61

List Total	Pop Hits	Pop Total	Fold Enrichment	Bonferroni	Benjamini	FDR
89	159	15908	3,37	1,00	0,83	95,57

74	159	12782	3,26	1,00	0,92	96,07
89	342	15908	1,57	1,00	0,95	100,00
74	342	12782	1,52	1,00	0,97	100,00

Annotation Cluster 62

List Total	Pop Hits	Pop Total	Fold Enrichment	Bonferroni	Benjamini	FDR
83	154	14116	3,31	1,00	0,91	98,43
81	154	13528	3,25	1,00	0,96	98,53
83	192	14116	2,66	1,00	0,94	99,74
81	192	13528	2,61	1,00	0,98	99,77
83	367	14116	1,85	1,00	0,95	99,93
81	367	13528	1,82	1,00	0,98	99,94
83	405	14116	1,68	1,00	0,96	99,99
81	405	13528	1,65	1,00	0,99	99,99
83	721	14116	1,18	1,00	0,99	100,00
81	721	13528	1,16	1,00	1,00	100,00

Annotation Cluster 63

List Total	Pop Hits	Pop Total	Fold Enrichment	Bonferroni	Benjamini	FDR
83	198	14116	2,58	1,00	0,95	99,81
81	198	13528	2,53	1,00	0,98	99,83
83	301	14116	1,70	1,00	0,98	100,00

Annotation Cluster 64

List Total	Pop Hits	Pop Total	Fold Enrichment	Bonferroni	Benjamini	FDR
89	345	15908	2,07	1,00	0,86	98,83

74	345	12782	2,00	1,00	0,93	99,08
97	241	19235	2,47	1,00	0,88	99,43
89	320	15908	1,68	1,00	0,94	99,99
74	320	12782	1,62	1,00	0,97	99,99

Annotation Cluster 65

List Total	Pop Hits	Pop Total	Fold Enrichment	Bonferroni	Benjamini	FDR
97	6256	19235	1,17	1,00	0,82	90,90
89	7266	15908	1,01	1,00	0,95	100,00
89	6578	15908	0,98	1,00	0,96	100,00

Annotation Cluster 66

List Total	Pop Hits	Pop Total	Fold Enrichment	Bonferroni	Benjamini	FDR
83	199	14116	2,56	1,00	0,95	99,82
81	199	13528	2,52	1,00	0,98	99,84
83	242	14116	2,11	1,00	0,96	99,98
81	242	13528	2,07	1,00	0,99	99,98
83	276	14116	1,85	1,00	0,97	100,00

81	276	13528	1,82	1,00	0,99	100,00

Annotation Cluster 67

List Total	Pop Hits	Pop Total	Fold Enrichment	Bonferroni	Benjamini	FDR
85	4140	15143	1,12	1,00	1,00	99,69
85	4179	15143	1,11	1,00	1,00	99,80
85	4241	15143	1,09	1,00	1,00	99,90
76	4140	12983	1,07	1,00	1,00	99,95
76	4179	12983	1,06	1,00	1,00	99,97
76	4241	12983	1,05	1,00	1,00	99,99

Annotation Cluster 68

List Total	Pop Hits	Pop Total	Fold Enrichment	Bonferroni	Benjamini	FDR
83	276	14116	2,46	1,00	0,90	98,10
81	276	13528	2,42	1,00	0,96	98,29
83	700	14116	1,21	1,00	0,99	100,00
83	701	14116	1,21	1,00	0,99	100,00
81	700	13528	1,19	1,00	1,00	100,00

81	701	13528	1,19	1,00	1,00	100,00

Annotation Cluster 69

List Total	Pop Hits	Pop Total	Fold Enrichment	Bonferroni	Benjamini	FDR
85	363	15143	1,96	1,00	1,00	99,53
76	363	12983	1,88	1,00	1,00	99,66
85	513	15143	1,39	1,00	1,00	100,00
76	513	12983	1,33	1,00	1,00	100,00

Annotation Cluster 70

List Total	Pop Hits	Pop Total	Fold Enrichment	Bonferroni	Benjamini	FDR
83	134	14116	3,81	1,00	0,88	96,26
81	134	13528	3,74	1,00	0,94	96,47
83	690	14116	0,99	1,00	1,00	100,00
81	690	13528	0,97	1,00	1,00	100,00
83	998	14116	0,85	1,00	1,00	100,00

Annotation Cluster 71

List Total	Pop Hits	Pop Total	Fold Enrichment	Bonferroni	Benjamini	FDR
44	99	5798	3,99	1,00	1,00	91,73
49	99	6441	3,98	1,00	1,00	92,70
44	115	5798	3,44	1,00	1,00	95,99
49	115	6441	3,43	1,00	1,00	96,58
44	131	5798	3,02	1,00	1,00	98,17
49	131	6441	3,01	1,00	1,00	98,50
38	432	5846	1,78	1,00	1,00	97,79
27	432	3811	1,63	1,00	1,00	98,82
49	166	6441	2,38	1,00	1,00	99,79
49	177	6441	2,23	1,00	1,00	99,89
38	518	5846	1,48	1,00	0,99	99,75
38	688	5846	1,34	1,00	0,99	99,85
27	518	3811	1,36	1,00	1,00	99,91
49	622	6441	1,27	1,00	1,00	99,99

27	688	3811	1,23	1,00	1,00	99,96
49	383	6441	1,37	1,00	1,00	100,00
49	284	6441	1,39	1,00	1,00	100,00
49	1024	6441	1,03	1,00	1,00	100,00
49	465	6441	1,13	1,00	1,00	100,00
49	481	6441	1,09	1,00	1,00	100,00
49	327	6441	1,21	1,00	1,00	100,00
49	636	6441	1,03	1,00	1,00	100,00
49	823	6441	0,96	1,00	1,00	100,00
49	685	6441	0,96	1,00	1,00	100,00
49	888	6441	0,89	1,00	1,00	100,00
49	541	6441	0,73	1,00	1,00	100,00
49	554	6441	0,71	1,00	1,00	100,00

Annotation Cluster 72

List Total	Pop Hits	Pop Total	Fold Enrichment	Bonferroni	Benjamini	FDR
97	264	19235	2,25	1,00	0,90	99,75
83	295	14116	1,73	1,00	0,98	100,00
81	295	13528	1,70	1,00	1,00	100,00

Annotation Cluster 73

List Total	Pop Hits	Pop Total	Fold Enrichment	Bonferroni	Benjamini	FDR
89	394	15908	1,81	1,00	0,90	99,71
74	394	12782	1,75	1,00	0,94	99,79
89	419	15908	1,71	1,00	0,92	99,87
74	419	12782	1,65	1,00	0,96	99,91
89	620	15908	1,44	1,00	0,93	99,95
89	622	15908	1,44	1,00	0,93	99,95
74	620	12782	1,39	1,00	0,96	99,97

74	622	12782	1,39	1,00	0,96	99,97
89	595	15908	1,20	1,00	0,96	100,00
74	595	12782	1,16	1,00	0,98	100,00

Annotation Cluster 74

List Total	Pop Hits	Pop Total	Fold Enrichment	Bonferroni	Benjamini	FDR
97	2026	19235	1,27	1,00	0,90	99,02
97	2071	19235	1,24	1,00	0,90	99,41
83	3542	14116	1,10	1,00	0,96	99,97
83	3442	14116	1,09	1,00	0,97	99,99
83	2101	14116	1,13	1,00	0,97	99,99
83	2812	14116	1,09	1,00	0,97	100,00
81	2101	13528	1,11	1,00	0,99	100,00
83	2832	14116	1,08	1,00	0,97	100,00
83	2999	14116	0,91	1,00	1,00	100,00
83	3409	14116	0,90	1,00	1,00	100,00

Annotation Cluster 75

List Total	Pop Hits	Pop Total	Fold Enrichment	Bonferroni	Benjamini	FDR
89	2596	15908	1,10	1,00	0,93	99,97

89	2596	15908	1,10	1,00	0,93	99,97
74	2596	12782	1,06	1,00	0,97	99,99
74	2596	12782	1,06	1,00	0,97	99,99

Annotation Cluster 76

List Total	Pop Hits	Pop Total	Fold Enrichment	Bonferroni	Benjamini	FDR
44	164	5798	2,41	1,00	1,00	99,69
49	164	6441	2,40	1,00	1,00	99,77
49	711	6441	1,29	1,00	1,00	99,97
49	225	6441	1,75	1,00	1,00	100,00
49	1024	6441	1,03	1,00	1,00	100,00
49	1210	6441	0,76	1,00	1,00	100,00

Annotation Cluster 77

List Total	Pop Hits	Pop Total	Fold Enrichment	Bonferroni	Benjamini	FDR
85	183	15143	2,92	1,00	1,00	98,59
76	183	12983	2,80	1,00	1,00	98,82
85	549	15143	1,30	1,00	1,00	100,00
76	549	12983	1,24	1,00	1,00	100,00
85	574	15143	1,24	1,00	1,00	100,00
76	574	12983	1,19	1,00	1,00	100,00
97	484	19235	1,23	1,00	0,99	100,00

Annotation Cluster 78

List Total	Pop Hits	Pop Total	Fold Enrichment	Bonferroni	Benjamini	FDR
83	431	14116	1,58	1,00	0,97	100,00
83	276	14116	1,85	1,00	0,97	100,00
81	276	13528	1,82	1,00	0,99	100,00
83	307	14116	1,66	1,00	0,98	100,00
83	307	14116	1,66	1,00	0,98	100,00
81	307	13528	1,63	1,00	1,00	100,00

81	307	13528	1,63	1,00	1,00	100,00

Annotation Cluster 79

List Total	Pop Hits	Pop Total	Fold Enrichment	Bonferroni	Benjamini	FDR
38	1029	5846	1,50	1,00	1,00	90,50
38	2542	5846	1,09	1,00	0,99	99,85
38	2546	5846	1,09	1,00	0,98	99,86
38	5421	5846	1,02	1,00	0,98	99,94
38	5421	5846	1,02	1,00	0,98	99,94
38	2137	5846	1,08	1,00	0,97	99,95
38	2140	5846	1,08	1,00	0,97	99,95
38	1131	5846	1,09	1,00	0,98	100,00
38	3058	5846	1,01	1,00	0,98	100,00
38	3598	5846	0,98	1,00	0,99	100,00

38	1419	5846	0,98	1,00	0,99	100,00
38	1788	5846	0,86	1,00	1,00	100,00

Annotation Cluster 80

List Total	Pop Hits	Pop Total	Fold Enrichment	Bonferroni	Benjamini	FDR
83	973	14116	1,22	1,00	0,98	100,00
83	973	14116	1,22	1,00	0,98	100,00
81	973	13528	1,20	1,00	1,00	100,00
81	973	13528	1,20	1,00	1,00	100,00
83	1182	14116	1,15	1,00	0,98	100,00

Annotation Cluster 81

List Total	Pop Hits	Pop Total	Fold Enrichment	Bonferroni	Benjamini	FDR
85	721	15143	1,48	1,00	1,00	99,81
85	606	15143	1,47	1,00	1,00	99,96
76	606	12983	1,41	1,00	1,00	99,98
97	542	19113	1,45	1,00	1,00	100,00
83	667	14116	1,27	1,00	0,98	100,00
81	667	13528	1,25	1,00	1,00	100,00
83	800	14116	1,06	1,00	1,00	100,00
81	800	13528	1,04	1,00	1,00	100,00

Annotation Cluster 82

List Total	Pop Hits	Pop Total	Fold Enrichment	Bonferroni	Benjamini	FDR
97	1326	19235	1,35	1,00	0,88	99,46
85	1477	15143	1,21	1,00	1,00	99,96

85	1497	15143	1,19	1,00	1,00	99,97
97	1686	19235	1,18	1,00	0,94	99,96
85	2245	15143	1,11	1,00	1,00	99,99
76	1477	12983	1,16	1,00	1,00	99,98
76	1497	12983	1,14	1,00	1,00	99,99
85	1577	15143	1,13	1,00	1,00	99,99
85	1601	15143	1,11	1,00	1,00	100,00
76	2245	12983	1,07	1,00	1,00	100,00
85	1612	15143	1,11	1,00	1,00	100,00
76	1577	12983	1,08	1,00	1,00	100,00
85	1836	15143	1,07	1,00	1,00	100,00
85	1836	15143	1,07	1,00	1,00	100,00
76	1601	12983	1,07	1,00	1,00	100,00
76	1612	12983	1,06	1,00	1,00	100,00
76	1836	12983	1,02	1,00	1,00	100,00
76	1836	12983	1,02	1,00	1,00	100,00
85	1918	15143	1,02	1,00	1,00	100,00
76	1918	12983	0,98	1,00	1,00	100,00

Annotation Cluster 83						
List Total	Pop Hits	Pop Total	Fold Enrichment	Bonferroni	Benjamini	FDR
83	2993	14116	1,08	1,00	0,97	100,00
83	2656	14116	1,09	1,00	0,97	100,00
83	2629	14116	0,97	1,00	1,00	100,00

Annotation Cluster 84						
List Total	Pop Hits	Pop Total	Fold Enrichment	Bonferroni	Benjamini	FDR
89	642	15908	1,39	1,00	0,93	99,97
74	642	12782	1,35	1,00	0,96	99,98
89	670	15908	1,33	1,00	0,94	99,99
74	670	12782	1,29	1,00	0,97	99,99
89	550	15908	1,30	1,00	0,95	100,00
89	568	15908	1,26	1,00	0,95	100,00
74	550	12782	1,26	1,00	0,97	100,00
74	568	12782	1,22	1,00	0,98	100,00

Annotation Cluster 85						
List Total	Pop Hits	Pop Total	Fold Enrichment	Bonferroni	Benjamini	FDR
97	205	19235	2,90	1,00	0,87	98,12
83	373	14116	1,37	1,00	0,99	100,00
83	566	14116	1,20	1,00	0,99	100,00
81	373	13528	1,34	1,00	1,00	100,00
81	566	13528	1,18	1,00	1,00	100,00
83	820	14116	1,04	1,00	1,00	100,00

Annotation Cluster 86

List Total	Pop Hits	Pop Total	Fold Enrichment	Bonferroni	Benjamini	FDR
49	711	6441	1,29	1,00	1,00	99,97
15	476	2121	1,19	1,00	1,00	100,00
25	478	3361	1,13	1,00	1,00	100,00

Annotation Cluster 87

List Total	Pop Hits	Pop Total	Fold Enrichment	Bonferroni	Benjamini	FDR
83	1453	14116	1,17	1,00	0,97	100,00
83	1526	14116	1,11	1,00	0,98	100,00
83	1182	14116	1,15	1,00	0,98	100,00
83	2355	14116	0,87	1,00	1,00	100,00

Annotation Cluster 88

List Total	Pop Hits	Pop Total	Fold Enrichment	Bonferroni	Benjamini	FDR
85	334	15143	1,60	1,00	1,00	100,00
76	334	12983	1,53	1,00	1,00	100,00
85	542	15143	1,31	1,00	1,00	100,00
76	542	12983	1,26	1,00	1,00	100,00

Annotation Cluster 89

List Total	Pop Hits	Pop Total	Fold Enrichment	Bonferroni	Benjamini	FDR
83	331	14116	1,54	1,00	0,99	100,00
83	334	14116	1,53	1,00	0,99	100,00
81	331	13528	1,51	1,00	1,00	100,00
81	334	13528	1,50	1,00	1,00	100,00

Annotation Cluster 90

List Total	Pop Hits	Pop Total	Fold Enrichment	Bonferroni	Benjamini	FDR
85	2311	15143	1,08	1,00	1,00	100,00
97	2189	19235	1,09	1,00	0,96	99,99
76	2311	12983	1,03	1,00	1,00	100,00
85	2785	15143	0,96	1,00	1,00	100,00
76	2785	12983	0,92	1,00	1,00	100,00

Annotation Cluster 91

List Total	Pop Hits	Pop Total	Fold Enrichment	Bonferroni	Benjamini	FDR
97	542	19113	1,45	1,00	1,00	100,00
89	354	16659	1,59	1,00	1,00	100,00
97	381	19235	1,56	1,00	0,97	100,00
85	430	15143	1,24	1,00	1,00	100,00
89	455	16659	1,23	1,00	1,00	100,00
76	430	12983	1,19	1,00	1,00	100,00
89	476	16659	1,18	1,00	1,00	100,00
97	658	19113	0,90	1,00	1,00	100,00

Annotation Cluster 92

List Total	Pop Hits	Pop Total	Fold Enrichment	Bonferroni	Benjamini	FDR
83	466	14116	1,46	1,00	0,98	100,00
81	466	13528	1,43	1,00	1,00	100,00
83	374	14116	1,36	1,00	0,99	100,00
83	380	14116	1,34	1,00	0,99	100,00
81	374	13528	1,34	1,00	1,00	100,00

81	380	13528	1,32	1,00	1,00	100,00
83	409	14116	1,25	1,00	1,00	100,00
81	409	13528	1,23	1,00	1,00	100,00
83	512	14116	1,00	1,00	1,00	100,00
81	512	13528	0,98	1,00	1,00	100,00

Annotation Cluster 93

List Total	Pop Hits	Pop Total	Fold Enrichment	Bonferroni	Benjamini	FDR
97	562	19235	1,41	1,00	0,97	99,99
83	639	14116	1,06	1,00	1,00	100,00
81	639	13528	1,05	1,00	1,00	100,00
85	689	15143	1,03	1,00	1,00	100,00

Annotation Cluster 94

List Total	Pop Hits	Pop Total	Fold Enrichment	Bonferroni	Benjamini	FDR
83	6636	14116	1,00	1,00	0,99	100,00
83	6923	14116	0,96	1,00	1,00	100,00
83	7647	14116	0,96	1,00	1,00	100,00

Annotation Cluster 95

List Total	Pop Hits	Pop Total	Fold Enrichment	Bonferroni	Benjamini	FDR

97	1868	19235	1,06	1,00	0,97	100,00
85	2331	15143	0,99	1,00	1,00	100,00
76	2331	12983	0,95	1,00	1,00	100,00
85	3264	15143	0,76	1,00	1,00	100,00

List Total	Pop Hits	Pop Total	Fold Enrichment	Bonferroni	Benjamini	FDR
83	487	14116	1,05	1,00	1,00	100,00
83	487	14116	1,05	1,00	1,00	100,00
81	487	13528	1,03	1,00	1,00	100,00
81	487	13528	1,03	1,00	1,00	100,00
83	762	14116	0,89	1,00	1,00	100,00
83	767	14116	0,89	1,00	1,00	100,00

Annotation Cluster 97

List Total	Pop Hits	Pop Total	Fold Enrichment	Bonferroni	Benjamini	FDR
97	484	19235	1,23	1,00	0,99	100,00
83	1054	14116	0,65	1,00	1,00	100,00
81	1054	13528	0,63	1,00	1,00	100,00

Annotation Cluster 98

List Total	Pop Hits	Pop Total	Fold Enrichment	Bonferroni	Benjamini	FDR
83	781	14116	0,87	1,00	1,00	100,00
81	781	13528	0,86	1,00	1,00	100,00
83	725	14116	0,70	1,00	1,00	100,00

81	725	13528	0,69	1,00	1,00	100,00

Annotation Cluster 99

List Total	Pop Hits	Pop Total	Fold Enrichment	Bonferroni	Benjamini	FDR
83	602	14116	0,85	1,00	1,00	100,00
83	611	14116	0,84	1,00	1,00	100,00
81	602	13528	0,83	1,00	1,00	100,00
81	611	13528	0,82	1,00	1,00	100,00
83	719	14116	0,71	1,00	1,00	100,00
83	724	14116	0,70	1,00	1,00	100,00
81	719	13528	0,70	1,00	1,00	100,00
81	724	13528	0,69	1,00	1,00	100,00

Annotation Cluster 100

List Total	Pop Hits	Pop Total	Fold Enrichment	Bonferroni	Benjamini	FDR
83	882	14116	0,77	1,00	1,00	100,00
81	882	13528	0,76	1,00	1,00	100,00
83	769	14116	0,66	1,00	1,00	100,00
81	769	13528	0,65	1,00	1,00	100,00

Annotation Cluster 101

List Total	Pop Hits	Pop Total	Fold Enrichment	Bonferroni	Benjamini	FDR
85	725	15143	0,74	1,00	1,00	100,00
85	827	15143	0,65	1,00	1,00	100,00
85	906	15143	0,59	1,00	1,00	100,00

Annotation Cluster 102

List Total	Pop Hits	Pop Total	Fold Enrichment	Bonferroni	Benjamini	FDR
89	1779	15908	0,70	1,00	1,00	100,00
89	1820	15908	0,69	1,00	1,00	100,00
74	1779	12782	0,68	1,00	1,00	100,00

89	1856	15908	0,67	1,00	1,00	100,00
74	1820	12782	0,66	1,00	1,00	100,00
74	1856	12782	0,65	1,00	1,00	100,00

Functional Annotation of Down-regulated Genes

Annotation Cluster 1	Fatty acid metabolism	Enrichment Score: 2,040712140204664			
Category	Term	Count	%	PValue	Genes
GOTERM_BP_ALL	GO:0006631~fatty acid metabolic process	5	14	5,91E-04	ACOX2, JMJD7-PLA2G4B, CYP2J2, ELOVL2, TMEM195
GOTERM_BP_FAT	GO:0006631~fatty acid metabolic process	5	14	6,00E-04	ACOX2, JMJD7-PLA2G4B, CYP2J2, ELOVL2, TMEM195
GOTERM_BP_ALL	GO:0032787~monocarboxylic acid metabolic process	5	14	0,003	ACOX2, JMJD7-PLA2G4B, CYP2J2, ELOVL2, TMEM195
GOTERM_BP_ALL	GO:0044255~cellular lipid metabolic process	5	14	0,019	ACOX2, JMJD7-PLA2G4B, CYP2J2, ELOVL2, TMEM195
GOTERM_BP_ALL	GO:0019752~carboxylic acid metabolic process	5	14	0,023	ACOX2, JMJD7-PLA2G4B, CYP2J2, ELOVL2, TMEM195
GOTERM_BP_ALL	GO:0043436~oxoacid metabolic process	5	14	0,023	ACOX2, JMJD7-PLA2G4B, CYP2J2, ELOVL2, TMEM195
GOTERM_BP_ALL	GO:0006082~organic acid metabolic process	5	14	0,024	ACOX2, JMJD7-PLA2G4B, CYP2J2, ELOVL2, TMEM195
GOTERM_BP_ALL	GO:0042180~cellular ketone metabolic process	5	14	0,025	ACOX2, JMJD7-PLA2G4B, CYP2J2, ELOVL2, TMEM195
GOTERM_BP_ALL	GO:0006629~lipid metabolic process	5	14	0,075	ACOX2, JMJD7-PLA2G4B, CYP2J2, ELOVL2, TMEM195

Annotation Cluster 2	Mitochondrion	Enrichment Score: 1,231951997706593			
Category	Term	Count	%	PValue	Genes
GOTERM_CC_FAT	GO:0005739~mitochondrion	6	16	0,056	IMMP2L, JMJD7-PLA2G4B, CREB1, CABC1, DNAJC19, CMPK2
GOTERM_CC_ALL	GO:0005739~mitochondrion	6	16	0,057	IMMP2L, JMJD7-PLA2G4B, CREB1, CABC1, DNAJC19, CMPK2
SP_PIR_KEYWORDS	mitochondrion	5	14	0,063	IMMP2L, JMJD7-PLA2G4B, CABC1, DNAJC19, CMPK2

Annotation Cluster 3	Membrane	Enrichment Score: 0,8200340131479906			
Category	Term	Count	%	PValue	Genes
GOTERM_CC_FAT	GO:0016021~integral to membrane	15	41	0,102	ABCB9, MGAT5B, IFITM1, NOXO1, TRPA1, GPR98, IMMP2L, SPAG4, ELOVL2, HS6ST3, TMPRSS12, DCAF17, NRG2, DNAJC19, TMEM195
GOTERM_CC_ALL	GO:0016021~integral to membrane	15	41	0,114	ABCB9, MGAT5B, IFITM1, NOXO1, TRPA1, GPR98, IMMP2L, SPAG4, ELOVL2, HS6ST3, TMPRSS12, DCAF17, NRG2, DNAJC19, TMEM195
SP_PIR_KEYWORDS	membrane	16	43	0,131	ABCB9, CYP2J2, MGAT5B, IFITM1, NOXO1, GPR98, IMMP2L, JMJD7-PLA2G4B, SPAG4, ELOVL2, HS6ST3, TMPRSS12, DCAF17, NRG2, DNAJC19, TMEM195

GOTERM_CC_FAT	GO:0031224~intrinsic to membrane	15	41	0,131	ABCB9, MGAT5B, IFITM1, NOXO1, TRPA1, GPR98, IMMP2L, SPAG4, ELOVL2, HS6ST3, TMPRSS12, DCAF17, NRG2, DNAJC19, TMEM195
GOTERM_CC_ALL	GO:0031224~intrinsic to membrane	15	41	0,144	ABCB9, MGAT5B, IFITM1, NOXO1, TRPA1, GPR98, IMMP2L, SPAG4, ELOVL2, HS6ST3, TMPRSS12, DCAF17, NRG2, DNAJC19, TMEM195
GOTERM_CC_ALL	GO:0044425~membrane part	17	46	0,164	ABCB9, CYP2J2, MGAT5B, IFITM1, NOXO1, TRPA1, GPR98, IMMP2L, JMJD7-PLA2G4B, SPAG4, ELOVL2, HS6ST3, TMPRSS12, DCAF17, NRG2, DNAJC19, TMEM195
UP_SEQ_FEATURE	transmembrane region	13	35	0,165	ABCB9, MGAT5B, IFITM1, GPR98, IMMP2L, SPAG4, ELOVL2, HS6ST3, TMPRSS12, DCAF17, NRG2, DNAJC19, TMEM195
SP_PIR_KEYWORDS	transmembrane	13	35	0,171	ABCB9, MGAT5B, IFITM1, GPR98, IMMP2L, SPAG4, ELOVL2, HS6ST3, TMPRSS12, DCAF17, NRG2, DNAJC19, TMEM195
GOTERM_CC_ALL	GO:0016020~membrane	17	46	0,313	ABCB9, CYP2J2, MGAT5B, IFITM1, NOXO1, TRPA1, GPR98, IMMP2L, JMJD7-PLA2G4B, SPAG4, ELOVL2, HS6ST3, TMPRSS12, DCAF17, NRG2, DNAJC19, TMEM195

**Annotation Cluster 4**	**Oxidoreductase activity**	**Enrichment Score: 0,8188194175139348**			
Category	Term	Count	%	PValue	Genes
GOTERM_BP_ALL	GO:0055114~oxidation reduction	4	11	0,131	ACOX2, CYP2J2, HTATIP2, TMEM195
GOTERM_BP_FAT	GO:0055114~oxidation reduction	4	11	0,133	ACOX2, CYP2J2, HTATIP2, TMEM195
GOTERM_MF_ALL	GO:0016491~oxidoreductase activity	4	11	0,200	ACOX2, CYP2J2, HTATIP2, TMEM195

**Annotation Cluster 5**	**Protein transport and localization**	**Enrichment Score: 0,7260943736083918**			
Category	Term	Count	%	PValue	Genes
GOTERM_BP_ALL	GO:0006886~intracellular protein transport	3	8	0,169	IMMP2L, STAP1, DNAJC19
GOTERM_BP_FAT	GO:0006886~intracellular protein transport	3	8	0,171	IMMP2L, STAP1, DNAJC19
GOTERM_BP_ALL	GO:0034613~cellular protein localization	3	8	0,196	IMMP2L, STAP1, DNAJC19
GOTERM_BP_FAT	GO:0034613~cellular protein localization	3	8	0,197	IMMP2L, STAP1, DNAJC19
GOTERM_BP_ALL	GO:0070727~cellular macromolecule localization	3	8	0,198	IMMP2L, STAP1, DNAJC19
GOTERM_BP_FAT	GO:0070727~cellular macromolecule localization	3	8	0,200	IMMP2L, STAP1, DNAJC19

**Annotation Cluster 6**	**Transport and localization**	**Enrichment Score: 0,7212367461632663**			
Category	Term	Count	%	PValue	Genes
GOTERM_BP_ALL	GO:0046907~intracellular transport	4	11	0,140	IMMP2L, HTATIP2, STAP1, DNAJC19
GOTERM_BP_FAT	GO:0046907~intracellular transport	4	11	0,141	IMMP2L, HTATIP2, STAP1, DNAJC19
GOTERM_BP_ALL	GO:0051649~establishment of localization in cell	4	11	0,237	IMMP2L, HTATIP2, STAP1, DNAJC19
GOTERM_BP_ALL	GO:0051641~cellular localization	4	11	0,279	IMMP2L, HTATIP2, STAP1, DNAJC19

**Annotation Cluster 7**	**Protein transport and localization**	**Enrichment Score: 0,6407169466358207**			
Category	Term	Count	%	PValue	Genes
GOTERM_BP_ALL	GO:0015031~protein transport	4	11	0,190	IMMP2L, ABCB9, STAP1, DNAJC19
GOTERM_BP_FAT	GO:0015031~protein transport	4	11	0,193	IMMP2L, ABCB9, STAP1, DNAJC19
GOTERM_BP_ALL	GO:0045184~establishment of protein localization	4	11	0,194	IMMP2L, ABCB9, STAP1, DNAJC19
GOTERM_BP_FAT	GO:0045184~establishment of protein localization	4	11	0,196	IMMP2L, ABCB9, STAP1, DNAJC19
GOTERM_BP_ALL	GO:0008104~protein localization	4	11	0,254	IMMP2L, ABCB9, STAP1, DNAJC19
GOTERM_BP_FAT	GO:0008104~protein localization	4	11	0,256	IMMP2L, ABCB9, STAP1, DNAJC19
GOTERM_BP_ALL	GO:0033036~macromolecule localization	4	11	0,361	IMMP2L, ABCB9, STAP1, DNAJC19

**Annotation Cluster 8**	**Catabolic activity**	**Enrichment Score: 0,6244122810601149**			
Category	Term	Count	%	PValue	Genes
GOTERM_MF_ALL	GO:0017111~nucleoside-triphosphatase activity	4	11	0,223	ABCB9, DDX60, MX1, DYNLRB2
GOTERM_MF_ALL	GO:0016462~pyrophosphatase activity	4	11	0,240	ABCB9, DDX60, MX1, DYNLRB2
GOTERM_MF_ALL	GO:0016818~hydrolase activity, acting on acid anhydrides, in phosphorus-containing anhydrides	4	11	0,242	ABCB9, DDX60, MX1, DYNLRB2
GOTERM_MF_ALL	GO:0016817~hydrolase activity, acting on acid anhydrides	4	11	0,245	ABCB9, DDX60, MX1, DYNLRB2

**Annotation Cluster 9**	**Catabolic activity**	**Enrichment Score: 0,6066796518131533**			
Category	Term	Count	%	PValue	Genes
GOTERM_BP_ALL	GO:0008152~metabolic process	9	24	0,049	USP18, CYP2J2, GSTA, HTATIP2, CREB1, LOC429567, TMPRSS12, TMEM195, CMPK2
GOTERM_BP_FAT	GO:0006508~proteolysis	3	8	0,096	USP18, LOC429567, TMPRSS12

GOTERM_BP_ALL	GO:0006508~proteolysis	3	8	0,119	USP18, LOC429567, TMPRSS12
GOTERM_MF_ALL	GO:0016787~hydrolase activity	5	14	0,182	USP18, ABCB9, LOC429567, TMPRSS12, MX1
GOTERM_BP_ALL	GO:0019538~protein metabolic process	4	11	0,329	USP18, CREB1, LOC429567, TMPRSS12
GOTERM_BP_ALL	GO:0044238~primary metabolic process	6	16	0,373	USP18, CREB1, LOC429567, TMPRSS12, TMEM195, CMPK2
GOTERM_BP_ALL	GO:0044237~cellular metabolic process	5	14	0,525	USP18, CREB1, LOC429567, TMEM195, CMPK2
GOTERM_BP_ALL	GO:0043170~macromolecule metabolic process	4	11	0,651	USP18, CREB1, LOC429567, TMPRSS12
GOTERM_BP_ALL	GO:0009987~cellular process	6	16	0,818	USP18, ABCB9, CREB1, LOC429567, TMEM195, CMPK2

**Annotation Cluster 10**	**Reproductive process**	**Enrichment Score: 0,5976336321295606**			
Category	Term	Count	%	PValue	Genes
GOTERM_BP_ALL	GO:0048609~reproductive process in a multicellular organism	3	8	0,251	ABCB9, CREB1, SPAG4
GOTERM_BP_ALL	GO:0032504~multicellular organism reproduction	3	8	0,251	ABCB9, CREB1, SPAG4
GOTERM_BP_FAT	GO:0032504~multicellular organism reproduction	3	8	0,254	ABCB9, CREB1, SPAG4
GOTERM_BP_FAT	GO:0048609~reproductive process in a multicellular organism	3	8	0,254	ABCB9, CREB1, SPAG4

**Annotation Cluster 11**	**Cytoskeleton**	**Enrichment Score: 0,47584185691055897**			
Category	Term	Count	%	PValue	Genes
GOTERM_CC_ALL	GO:0005874~microtubule	3	8	0,099	SPAG4, DYNLRB2, RANBP10
GOTERM_CC_FAT	GO:0005874~microtubule	3	8	0,100	SPAG4, DYNLRB2, RANBP10
GOTERM_CC_ALL	GO:0015630~microtubule cytoskeleton	3	8	0,290	SPAG4, DYNLRB2, RANBP10
GOTERM_CC_FAT	GO:0015630~microtubule cytoskeleton	3	8	0,292	SPAG4, DYNLRB2, RANBP10
GOTERM_CC_ALL	GO:0044430~cytoskeletal part	3	8	0,561	SPAG4, DYNLRB2, RANBP10
GOTERM_CC_FAT	GO:0044430~cytoskeletal part	3	8	0,565	SPAG4, DYNLRB2, RANBP10
GOTERM_CC_ALL	GO:0005856~cytoskeleton	3	8	0,764	SPAG4, DYNLRB2, RANBP10
GOTERM_CC_FAT	GO:0005856~cytoskeleton	3	8	0,769	SPAG4, DYNLRB2, RANBP10

**Annotation Cluster 12**	**Organelle**	**Enrichment Score: 0,46396721606100505**			
Category	Term	Count	%	PValue	Genes
GOTERM_CC_ALL	GO:0031967~organelle envelope	3	8	0,342	IMMP2L, HTATIP2, DNAJC19
GOTERM_CC_ALL	GO:0031975~envelope	3	8	0,343	IMMP2L, HTATIP2, DNAJC19

GOTERM_CC_FAT	GO:0031967~organelle envelope	3	8	0,344	IMMP2L, HTATIP2, DNAJC19
GOTERM_CC_FAT	GO:0031975~envelope	3	8	0,345	IMMP2L, HTATIP2, DNAJC19

**Annotation Cluster 13**	**Cytoplasm**	**Enrichment Score: 0,4161976873942851**			
Category	Term	Count	%	PValue	Genes
SP_PIR_KEYWORDS	cytoplasm	3	8	0,132	GSTA, GSTT1, MX1
GOTERM_CC_ALL	GO:0005737~cytoplasm	4	11	0,267	GSTA, GSTT1, MX1, TMEM195
GOTERM_CC_ALL	GO:0044424~intracellular part	5	14	0,388	GSTA, CREB1, GSTT1, MX1, TMEM195
GOTERM_CC_ALL	GO:0005622~intracellular	5	14	0,576	GSTA, CREB1, GSTT1, MX1, TMEM195
GOTERM_CC_ALL	GO:0005623~cell	7	19	0,636	ABCB9, GSTA, CREB1, GSTT1, MX1, LOC418109, TMEM195
GOTERM_CC_ALL	GO:0044464~cell part	7	19	0,636	ABCB9, GSTA, CREB1, GSTT1, MX1, LOC418109, TMEM195

**Annotation Cluster 14**	**Proteolitic activity**	**Enrichment Score: 0,41038783255096956**			
Category	Term	Count	%	PValue	Genes
SP_PIR_KEYWORDS	Protease	3	8	0,220	IMMP2L, USP18, TMPRSS12
GOTERM_MF_FAT	GO:0070011~peptidase activity, acting on L-amino acid peptides	3	8	0,333	IMMP2L, USP18, TMPRSS12
GOTERM_MF_ALL	GO:0070011~peptidase activity, acting on L-amino acid peptides	3	8	0,351	IMMP2L, USP18, TMPRSS12
GOTERM_MF_FAT	GO:0008233~peptidase activity	3	8	0,353	IMMP2L, USP18, TMPRSS12
GOTERM_MF_ALL	GO:0008233~peptidase activity	3	8	0,371	IMMP2L, USP18, TMPRSS12
GOTERM_BP_ALL	GO:0006508~proteolysis	3	8	0,629	IMMP2L, USP18, TMPRSS12
GOTERM_BP_FAT	GO:0006508~proteolysis	3	8	0,633	IMMP2L, USP18, TMPRSS12

**Annotation Cluster 15**	**Nucleotide binding**	**Enrichment Score: 0,2837008226120095**			
Category	Term	Count	%	PValue	Genes
SP_PIR_KEYWORDS	nucleotide-binding	5	14	0,369	ABCB9, DDX60, CABC1, MX1, CMPK2
GOTERM_MF_FAT	GO:0017076~purine nucleotide binding	6	16	0,400	ACOX2, ABCB9, DDX60, CABC1, MX1, CMPK2
GOTERM_MF_ALL	GO:0017076~purine nucleotide binding	6	16	0,435	ACOX2, ABCB9, DDX60, CABC1, MX1, CMPK2
SP_PIR_KEYWORDS	atp-binding	4	11	0,438	ABCB9, DDX60, CABC1, CMPK2
GOTERM_MF_FAT	GO:0030554~adenyl nucleotide binding	5	14	0,448	ACOX2, ABCB9, DDX60, CABC1, CMPK2
GOTERM_MF_FAT	GO:0001883~purine nucleoside binding	5	14	0,460	ACOX2, ABCB9, DDX60, CABC1, CMPK2
GOTERM_MF_FAT	GO:0001882~nucleoside binding	5	14	0,465	ACOX2, ABCB9, DDX60, CABC1, CMPK2
GOTERM_MF_ALL	GO:0030554~adenyl nucleotide binding	5	14	0,479	ACOX2, ABCB9, DDX60, CABC1, CMPK2
GOTERM_MF_ALL	GO:0001883~purine nucleoside binding	5	14	0,491	ACOX2, ABCB9, DDX60, CABC1, CMPK2

GOTERM_MF_ALL	GO:0001882~nucleoside binding	5	14	0,496	ACOX2, ABCB9, DDX60, CABC1, CMPK2
GOTERM_MF_FAT	GO:0000166~nucleotide binding	6	16	0,546	ACOX2, ABCB9, DDX60, CABC1, MX1, CMPK2
GOTERM_MF_FAT	GO:0032555~purine ribonucleotide binding	5	14	0,573	ABCB9, DDX60, CABC1, MX1, CMPK2
GOTERM_MF_FAT	GO:0032553~ribonucleotide binding	5	14	0,573	ABCB9, DDX60, CABC1, MX1, CMPK2
GOTERM_MF_ALL	GO:0000166~nucleotide binding	6	16	0,581	ACOX2, ABCB9, DDX60, CABC1, MX1, CMPK2
GOTERM_MF_ALL	GO:0032553~ribonucleotide binding	5	14	0,604	ABCB9, DDX60, CABC1, MX1, CMPK2
GOTERM_MF_ALL	GO:0032555~purine ribonucleotide binding	5	14	0,604	ABCB9, DDX60, CABC1, MX1, CMPK2
GOTERM_MF_FAT	GO:0005524~ATP binding	4	11	0,632	ABCB9, DDX60, CABC1, CMPK2
GOTERM_MF_FAT	GO:0032559~adenyl ribonucleotide binding	4	11	0,642	ABCB9, DDX60, CABC1, CMPK2
GOTERM_MF_ALL	GO:0005524~ATP binding	4	11	0,658	ABCB9, DDX60, CABC1, CMPK2
GOTERM_MF_ALL	GO:0032559~adenyl ribonucleotide binding	4	11	0,667	ABCB9, DDX60, CABC1, CMPK2
**Annotation Cluster 16**	**Apoptosis**	**Enrichment Score: 0,24559109466733536**			
Category	Term	Count	%	PValue	Genes
GOTERM_BP_ALL	GO:0042981~regulation of apoptosis	3	8	0,479	HTATIP2, CREB1, MX1
GOTERM_BP_FAT	GO:0042981~regulation of apoptosis	3	8	0,483	HTATIP2, CREB1, MX1
GOTERM_BP_ALL	GO:0043067~regulation of programmed cell death	3	8	0,485	HTATIP2, CREB1, MX1
GOTERM_BP_ALL	GO:0010941~regulation of cell death	3	8	0,486	HTATIP2, CREB1, MX1
GOTERM_BP_FAT	GO:0043067~regulation of programmed cell death	3	8	0,488	HTATIP2, CREB1, MX1
GOTERM_BP_FAT	GO:0010941~regulation of cell death	3	8	0,490	HTATIP2, CREB1, MX1
GOTERM_BP_ALL	GO:0048522~positive regulation of cellular process	3	8	0,897	HTATIP2, CREB1, MX1
GOTERM_BP_ALL	GO:0048518~positive regulation of biological process	3	8	0,927	HTATIP2, CREB1, MX1
**Annotation Cluster 17**	**Organelle**	**Enrichment Score: 0,22878901435948978**			
Category	Term	Count	%	PValue	Genes
GOTERM_CC_ALL	GO:0043228~non-membrane-bounded organelle	6	16	0,586	CREB1, SPAG4, TRPA1, DYNLRB2, DCAF17, RANBP10
GOTERM_CC_ALL	GO:0043232~intracellular non-membrane-bounded organelle	6	16	0,586	CREB1, SPAG4, TRPA1, DYNLRB2, DCAF17, RANBP10
GOTERM_CC_FAT	GO:0043232~intracellular non-membrane-bounded organelle	6	16	0,595	CREB1, SPAG4, TRPA1, DYNLRB2, DCAF17, RANBP10
GOTERM_CC_FAT	GO:0043228~non-membrane-bounded organelle	6	16	0,595	CREB1, SPAG4, TRPA1, DYNLRB2, DCAF17, RANBP10

Annotation Cluster 18	Sensory perception	Enrichment Score: 0,22006307509956402			
Category	Term	Count	%	PValue	Genes
GOTERM_BP_ALL	GO:0007600~sensory perception	3	8	0,483	TRPA1, DNAJC19, GPR98
GOTERM_BP_FAT	GO:0007600~sensory perception	3	8	0,487	TRPA1, DNAJC19, GPR98
GOTERM_BP_ALL	GO:0050890~cognition	3	8	0,546	TRPA1, DNAJC19, GPR98
GOTERM_BP_FAT	GO:0050890~cognition	3	8	0,550	TRPA1, DNAJC19, GPR98
GOTERM_BP_ALL	GO:0050877~neurological system process	3	8	0,705	TRPA1, DNAJC19, GPR98
GOTERM_BP_FAT	GO:0050877~neurological system process	3	8	0,709	TRPA1, DNAJC19, GPR98
GOTERM_BP_ALL	GO:0003008~system process	3	8	0,815	TRPA1, DNAJC19, GPR98

Annotation Cluster 19	Metabolic process	Enrichment Score: 0,20416541409102573			
Category	Term	Count	%	PValue	Genes
GOTERM_BP_ALL	GO:0044237~cellular metabolic process	14	38	0,598	ACOX2, CYP2J2, NOXO1, CREB1, ABTB1, SNORA32, GSTT1, CMPK2, IMMP2L, JMJD7-PLA2G4B, USP18, ELOVL2, DNAJC19, TMEM195
GOTERM_BP_ALL	GO:0008152~metabolic process	16	43	0,602	ACOX2, HTATIP2, CYP2J2, NOXO1, CREB1, ABTB1, SNORA32, GSTT1, CMPK2, IMMP2L, JMJD7-PLA2G4B, USP18, ELOVL2, TMPRSS12, DNAJC19, TMEM195
GOTERM_BP_ALL	GO:0044238~primary metabolic process	14	38	0,679	ACOX2, CYP2J2, CREB1, ABTB1, SNORA32, GSTT1, CMPK2, IMMP2L, JMJD7-PLA2G4B, USP18, ELOVL2, TMPRSS12, DNAJC19, TMEM195

Annotation Cluster 20	Transport and localization	Enrichment Score: 0,18111611011189943			
Category	Term	Count	%	PValue	Genes
GOTERM_BP_ALL	GO:0006810~transport	6	16	0,618	IMMP2L, ABCB9, HTATIP2, STAP1, TRPA1, DNAJC19
GOTERM_BP_ALL	GO:0051234~establishment of localization	6	16	0,628	IMMP2L, ABCB9, HTATIP2, STAP1, TRPA1, DNAJC19
GOTERM_BP_ALL	GO:0051179~localization	6	16	0,738	IMMP2L, ABCB9, HTATIP2, STAP1, TRPA1, DNAJC19

Annotation Cluster 21	Development	Enrichment Score: 0,07778930418795711			
Category	Term	Count	%	PValue	Genes
GOTERM_BP_ALL	GO:0048513~organ development	4	11	0,688	HTATIP2, CREB1, DNAJC19, GPR98
GOTERM_BP_ALL	GO:0007275~multicellular organismal development	5	14	0,849	HTATIP2, CREB1, NRG2, DNAJC19, GPR98
GOTERM_BP_ALL	GO:0048731~system development	4	11	0,864	HTATIP2, CREB1, DNAJC19, GPR98
GOTERM_BP_ALL	GO:0032502~developmental process	5	14	0,900	HTATIP2, CREB1, NRG2, DNAJC19, GPR98

GOTERM_BP_ALL	GO:0048856~anatomical structure development	4	11	0,900	HTATIP2, CREB1, DNAJC19, GPR98

**Annotation Cluster 22**	**Biological regulation**	**Enrichment Score: 0,029813859892181764**			
Category	Term	Count	%	PValue	Genes
GOTERM_BP_ALL	GO:0050789~regulation of biological process	12	32	0,914	JMJD7-PLA2G4B, CYP2J2, HTATIP2, IFITM1, STAP1, NOXO1, CREB1, SNORA32, MX1, NRG2, GPR98, CCDC79
GOTERM_BP_ALL	GO:0050794~regulation of cellular process	11	30	0,937	JMJD7-PLA2G4B, HTATIP2, IFITM1, STAP1, NOXO1, CREB1, SNORA32, MX1, NRG2, GPR98, CCDC79
GOTERM_BP_ALL	GO:0065007~biological regulation	12	32	0,950	JMJD7-PLA2G4B, CYP2J2, HTATIP2, IFITM1, STAP1, NOXO1, CREB1, SNORA32, MX1, NRG2, GPR98, CCDC79

**Annotation Cluster 23**	**Regulation of transcription**	**Enrichment Score: 0,0251153768605488**			
Category	Term	Count	%	PValue	Genes
GOTERM_BP_ALL	GO:0045449~regulation of transcription	4	11	0,911	HTATIP2, CREB1, SNORA32, CCDC79
GOTERM_BP_FAT	GO:0045449~regulation of transcription	4	11	0,915	HTATIP2, CREB1, SNORA32, CCDC79
GOTERM_BP_ALL	GO:0019219~regulation of nucleobase, nucleoside, nucleotide and nucleic acid metabolic process	4	11	0,938	HTATIP2, CREB1, SNORA32, CCDC79
GOTERM_BP_ALL	GO:0031323~regulation of cellular metabolic process	5	14	0,939	HTATIP2, NOXO1, CREB1, SNORA32, CCDC79
GOTERM_BP_ALL	GO:0010556~regulation of macromolecule biosynthetic process	4	11	0,940	HTATIP2, CREB1, SNORA32, CCDC79
GOTERM_BP_ALL	GO:0051171~regulation of nitrogen compound metabolic process	4	11	0,941	HTATIP2, CREB1, SNORA32, CCDC79
GOTERM_BP_ALL	GO:0010468~regulation of gene expression	4	11	0,942	HTATIP2, CREB1, SNORA32, CCDC79
GOTERM_BP_ALL	GO:0031326~regulation of cellular biosynthetic process	4	11	0,951	HTATIP2, CREB1, SNORA32, CCDC79
GOTERM_BP_ALL	GO:0009889~regulation of biosynthetic process	4	11	0,952	HTATIP2, CREB1, SNORA32, CCDC79
GOTERM_BP_ALL	GO:0019222~regulation of metabolic process	5	14	0,953	HTATIP2, NOXO1, CREB1, SNORA32, CCDC79
GOTERM_BP_ALL	GO:0060255~regulation of macromolecule metabolic process	4	11	0,972	HTATIP2, CREB1, SNORA32, CCDC79
GOTERM_BP_ALL	GO:0080090~regulation of primary metabolic process	4	11	0,974	HTATIP2, CREB1, SNORA32, CCDC79

Annotation Cluster 24	Membrane	Enrichment Score: 0,019975666518805228			
Category	Term	Count	%	PValue	Genes
UP_SEQ_FEATURE	topological domain:Cytoplasmic	5	14	0,888	MGAT5B, HS6ST3, TMPRSS12, NRG2, GPR98
UP_SEQ_FEATURE	glycosylation site:N-linked (GlcNAc,,,)	4	11	0,989	MGAT5B, HS6ST3, TMPRSS12, NRG2
SP_PIR_KEYWORDS	glycoprotein	4	11	0,992	MGAT5B, HS6ST3, TMPRSS12, NRG2

Annotation Cluster 25	Metal ion binding	Enrichment Score: 0,0033735008096264637			
Category	Term	Count	%	PValue	Genes
GOTERM_MF_FAT	GO:0046872~metal ion binding	5	14	0,991	JMJD7-PLA2G4B, CYP2J2, MGAT5B, GPR98, TMEM195
GOTERM_MF_FAT	GO:0043169~cation binding	5	14	0,992	JMJD7-PLA2G4B, CYP2J2, MGAT5B, GPR98, TMEM195
GOTERM_MF_ALL	GO:0046872~metal ion binding	5	14	0,992	JMJD7-PLA2G4B, CYP2J2, MGAT5B, GPR98, TMEM195
GOTERM_MF_FAT	GO:0043167~ion binding	5	14	0,993	JMJD7-PLA2G4B, CYP2J2, MGAT5B, GPR98, TMEM195
GOTERM_MF_ALL	GO:0043169~cation binding	5	14	0,993	JMJD7-PLA2G4B, CYP2J2, MGAT5B, GPR98, TMEM195
GOTERM_MF_ALL	GO:0043167~ion binding	5	14	0,994	JMJD7-PLA2G4B, CYP2J2, MGAT5B, GPR98, TMEM195

Annotation Cluster 26	Transcription	Enrichment Score: 0,001225773783345078			
Category	Term	Count	%	PValue	Genes
GOTERM_BP_ALL	GO:0006139~nucleobase, nucleoside, nucleotide and nucleic acid metabolic process	3	8	0,996	CREB1, SNORA32, CMPK2
GOTERM_BP_ALL	GO:0034641~cellular nitrogen compound metabolic process	3	8	0,998	CREB1, SNORA32, CMPK2
GOTERM_BP_ALL	GO:0006807~nitrogen compound metabolic process	3	8	0,998	CREB1, SNORA32, CMPK2

Annotation Cluster 1

List Total	Pop Hits	Pop Total	Fold Enrichment	Bonferroni	Benjamini	FDR
29	198	14116	12,29	0,19	0,19	0,81
28	198	13528	12,20	0,15	0,15	0,78
29	301	14116	8,09	0,64	0,40	3,75
29	526	14116	4,63	1,00	0,91	23,41
29	556	14116	4,38	1,00	0,88	27,42
29	556	14116	4,38	1,00	0,88	27,42
29	560	14116	4,35	1,00	0,82	27,97
29	567	14116	4,29	1,00	0,78	28,95
29	813	14116	2,99	1,00	0,96	65,57

Annotation Cluster 2

List Total	Pop Hits	Pop Total	Fold Enrichment	Bonferroni	Benjamini	FDR
26	1087	12782	2,71	0,99	0,99	45,24
32	1087	15908	2,74	1,00	0,74	47,28
36	832	19235	3,21	1,00	1,00	51,70

Annotation Cluster 3

List Total	Pop Hits	Pop Total	Fold Enrichment	Bonferroni	Benjamini	FDR
26	5297	12782	1,39	1,00	0,93	67,38
32	5297	15908	1,41	1,00	0,75	73,31
36	6256	19235	1,37	1,00	1,00	79,20

26	5485	12782	1,34	1,00	0,92	77,02
32	5485	15908	1,36	1,00	0,69	81,68
32	6578	15908	1,28	1,00	0,71	85,76
36	4911	19113	1,41	1,00	1,00	88,55
36	4973	19235	1,40	1,00	1,00	87,67
32	7266	15908	1,16	1,00	0,83	98,34

Annotation Cluster 4

List Total	Pop Hits	Pop Total	Fold Enrichment	Bonferroni	Benjamini	FDR
29	639	14116	3,05	1,00	0,99	85,60
28	639	13528	3,02	1,00	1,00	84,60
35	689	15143	2,51	1,00	1,00	93,29

Annotation Cluster 5

List Total	Pop Hits	Pop Total	Fold Enrichment	Bonferroni	Benjamini	FDR
29	374	14116	3,90	1,00	0,99	92,19
28	374	13528	3,88	1,00	1,00	91,40
29	411	14116	3,55	1,00	0,99	95,00
28	411	13528	3,53	1,00	1,00	94,40
29	414	14116	3,53	1,00	0,99	95,18
28	414	13528	3,50	1,00	1,00	94,60

Annotation Cluster 6

List Total	Pop Hits	Pop Total	Fold Enrichment	Bonferroni	Benjamini	FDR
29	657	14116	2,96	1,00	0,99	87,36
28	657	13528	2,94	1,00	1,00	86,41
29	852	14116	2,29	1,00	1,00	97,60
29	928	14116	2,10	1,00	1,00	98,88

Annotation Cluster 7

List Total	Pop Hits	Pop Total	Fold Enrichment	Bonferroni	Benjamini	FDR
29	762	14116	2,56	1,00	1,00	94,53
28	762	13528	2,54	1,00	1,00	93,95
29	769	14116	2,53	1,00	0,99	94,85
28	769	13528	2,51	1,00	1,00	94,29
29	882	14116	2,21	1,00	1,00	98,21
28	882	13528	2,19	1,00	1,00	97,94
29	1076	14116	1,81	1,00	1,00	99,79

Annotation Cluster 8

List Total	Pop Hits	Pop Total	Fold Enrichment	Bonferroni	Benjamini	FDR
35	728	15143	2,38	1,00	1,00	95,28
35	757	15143	2,29	1,00	1,00	96,41
35	760	15143	2,28	1,00	1,00	96,52
35	764	15143	2,27	1,00	1,00	96,65

Annotation Cluster 9

List Total	Pop Hits	Pop Total	Fold Enrichment	Bonferroni	Benjamini	FDR
11	3165	6441	1,67	1,00	1,00	45,97
9	389	5798	4,97	1,00	1,00	67,80

11	389	6441	4,52	1,00	1,00	78,93
16	1222	8067	2,06	1,00	1,00	87,82
11	1302	6441	1,80	1,00	1,00	99,26
11	2593	6441	1,35	1,00	1,00	99,68
11	2346	6441	1,25	1,00	1,00	99,99
11	2015	6441	1,16	1,00	1,00	100,00
11	3806	6441	0,92	1,00	1,00	100,00

Annotation Cluster 10

List Total	Pop Hits	Pop Total	Fold Enrichment	Bonferroni	Benjamini	FDR
29	487	14116	3,00	1,00	1,00	98,14
29	487	14116	3,00	1,00	1,00	98,14
28	487	13528	2,98	1,00	1,00	97,84
28	487	13528	2,98	1,00	1,00	97,84

Annotation Cluster 11

List Total	Pop Hits	Pop Total	Fold Enrichment	Bonferroni	Benjamini	FDR
32	274	15908	5,44	1,00	0,85	68,00
26	274	12782	5,38	1,00	0,98	66,52
32	549	15908	2,72	1,00	0,82	97,62
26	549	12782	2,69	1,00	0,99	97,28
32	952	15908	1,57	1,00	0,96	99,99
26	952	12782	1,55	1,00	1,00	99,98
32	1381	15908	1,08	1,00	0,99	100,00
26	1381	12782	1,07	1,00	1,00	100,00

Annotation Cluster 12

List Total	Pop Hits	Pop Total	Fold Enrichment	Bonferroni	Benjamini	FDR
32	620	15908	2,41	1,00	0,85	98,95
32	622	15908	2,40	1,00	0,84	98,98

26	620	12782	2,38	1,00	0,99	98,77
26	622	12782	2,37	1,00	0,98	98,80

Annotation Cluster 13

List Total	Pop Hits	Pop Total	Fold Enrichment	Bonferroni	Benjamini	FDR
6	443	3361	3,79	0,84	0,84	60,88
7	1788	5846	1,87	1,00	1,00	92,52
7	3058	5846	1,37	1,00	1,00	98,35
7	3598	5846	1,16	1,00	1,00	99,92
7	5421	5846	1,08	1,00	1,00	99,98
7	5421	5846	1,08	1,00	1,00	99,98

Annotation Cluster 14

List Total	Pop Hits	Pop Total	Fold Enrichment	Bonferroni	Benjamini	FDR
36	484	19235	3,31	1,00	1,00	93,77
29	549	12983	2,45	1,00	1,00	99,08
35	549	15143	2,36	1,00	1,00	99,46
29	574	12983	2,34	1,00	1,00	99,35
35	574	15143	2,26	1,00	1,00	99,64
29	1054	14116	1,39	1,00	1,00	100,00
28	1054	13528	1,38	1,00	1,00	100,00

Annotation Cluster 15

List Total	Pop Hits	Pop Total	Fold Enrichment	Bonferroni	Benjamini	FDR
36	1686	19235	1,58	1,00	1,00	99,41
29	1918	12983	1,40	1,00	1,00	99,73
35	1918	15143	1,35	1,00	1,00	99,90
36	1326	19235	1,61	1,00	0,99	99,84
29	1577	12983	1,42	1,00	1,00	99,90
29	1601	12983	1,40	1,00	1,00	99,92
29	1612	12983	1,39	1,00	1,00	99,93
35	1577	15143	1,37	1,00	1,00	99,96
35	1601	15143	1,35	1,00	1,00	99,97

35	1612	15143	1,34	1,00	1,00	99,98
29	2245	12983	1,20	1,00	1,00	99,99
29	1836	12983	1,22	1,00	1,00	99,99
29	1836	12983	1,22	1,00	1,00	99,99
35	2245	15143	1,16	1,00	1,00	100,00
35	1836	15143	1,18	1,00	1,00	100,00
35	1836	15143	1,18	1,00	1,00	100,00
29	1477	12983	1,21	1,00	1,00	100,00
29	1497	12983	1,20	1,00	1,00	100,00
35	1477	15143	1,17	1,00	1,00	100,00
35	1497	15143	1,16	1,00	1,00	100,00

Annotation Cluster 16

List Total	Pop Hits	Pop Total	Fold Enrichment	Bonferroni	Benjamini	FDR
29	804	14116	1,82	1,00	1,00	99,99
28	804	13528	1,80	1,00	1,00	99,98
29	812	14116	1,80	1,00	1,00	99,99
29	815	14116	1,79	1,00	1,00	99,99
28	812	13528	1,79	1,00	1,00	99,98
28	815	13528	1,78	1,00	1,00	99,99
29	1847	14116	0,79	1,00	1,00	100,00
29	2033	14116	0,72	1,00	1,00	100,00

Annotation Cluster 17

List Total	Pop Hits	Pop Total	Fold Enrichment	Bonferroni	Benjamini	FDR
32	2596	15908	1,15	1,00	0,96	99,99
32	2596	15908	1,15	1,00	0,96	99,99
26	2596	12782	1,14	1,00	1,00	99,99
26	2596	12782	1,14	1,00	1,00	99,99

Annotation Cluster 18

List Total	Pop Hits	Pop Total	Fold Enrichment	Bonferroni	Benjamini	FDR
29	810	14116	1,80	1,00	1,00	99,99
28	810	13528	1,79	1,00	1,00	99,98
29	909	14116	1,61	1,00	1,00	100,00
28	909	13528	1,59	1,00	1,00	100,00
29	1210	14116	1,21	1,00	1,00	100,00
28	1210	13528	1,20	1,00	1,00	100,00
29	1503	14116	0,97	1,00	1,00	100,00

Annotation Cluster 19

List Total	Pop Hits	Pop Total	Fold Enrichment	Bonferroni	Benjamini	FDR
29	6636	14116	1,03	1,00	1,00	100,00
29	7647	14116	1,02	1,00	1,00	100,00
29	6923	14116	0,98	1,00	1,00	100,00

Annotation Cluster 20

List Total	Pop Hits	Pop Total	Fold Enrichment	Bonferroni	Benjamini	FDR
29	2629	14116	1,11	1,00	1,00	100,00
29	2656	14116	1,10	1,00	1,00	100,00
29	2993	14116	0,98	1,00	1,00	100,00

Annotation Cluster 21

List Total	Pop Hits	Pop Total	Fold Enrichment	Bonferroni	Benjamini	FDR
29	1738	14116	1,12	1,00	1,00	100,00
29	2865	14116	0,85	1,00	1,00	100,00
29	2330	14116	0,84	1,00	1,00	100,00
29	3148	14116	0,77	1,00	1,00	100,00

29	2527	14116	0,77	1,00	1,00	100,00

Annotation Cluster 22

List Total	Pop Hits	Pop Total	Fold Enrichment	Bonferroni	Benjamini	FDR
29	7106	14116	0,82	1,00	1,00	100,00
29	6819	14116	0,79	1,00	1,00	100,00
29	7484	14116	0,78	1,00	1,00	100,00

Annotation Cluster 23

List Total	Pop Hits	Pop Total	Fold Enrichment	Bonferroni	Benjamini	FDR
29	2601	14116	0,75	1,00	1,00	100,00
28	2601	13528	0,74	1,00	1,00	100,00
29	2814	14116	0,69	1,00	1,00	100,00
29	3464	14116	0,70	1,00	1,00	100,00
29	2830	14116	0,69	1,00	1,00	100,00
29	2839	14116	0,69	1,00	1,00	100,00
29	2856	14116	0,68	1,00	1,00	100,00
29	2947	14116	0,66	1,00	1,00	100,00
29	2966	14116	0,66	1,00	1,00	100,00
29	3621	14116	0,67	1,00	1,00	100,00
29	3259	14116	0,60	1,00	1,00	100,00
29	3291	14116	0,59	1,00	1,00	100,00

Annotation Cluster 24

List Total	Pop Hits	Pop Total	Fold Enrichment	Bonferroni	Benjamini	FDR
36	3374	19113	0,79	1,00	1,00	100,00
36	4129	19113	0,51	1,00	1,00	100,00
36	4318	19235	0,49	1,00	1,00	100,00

Annotation Cluster 25

List Total	Pop Hits	Pop Total	Fold Enrichment	Bonferroni	Benjamini	FDR
29	4140	12983	0,54	1,00	1,00	100,00
29	4179	12983	0,54	1,00	1,00	100,00
35	4140	15143	0,52	1,00	1,00	100,00
29	4241	12983	0,53	1,00	1,00	100,00
35	4179	15143	0,52	1,00	1,00	100,00
35	4241	15143	0,51	1,00	1,00	100,00

Annotation Cluster 26

List Total	Pop Hits	Pop Total	Fold Enrichment	Bonferroni	Benjamini	FDR
29	3409	14116	0,43	1,00	1,00	100,00
29	3670	14116	0,40	1,00	1,00	100,00
29	3778	14116	0,39	1,00	1,00	100,00

Functional category	Functional Annotation Cluster	Enrichment Score
**Up-regulated**		
Tissue development and maintenance	Development	3,781
	Muscle development	3,298
	Nervous system development	3,007
	Skeletal muscle development	2,167
	Muscle cell differentiation	2,042
	Neuron development	1,857
	Neuron signaling	1,543
	Response to stimulus	1,190
	Regulation of neuron development	1,102
	Respiratory development	0,889
	Negative regulation of differentiation and development	0,823
	Synaptic transmission	0,756
	Bone development	0,625
	Response to stimulus	0,586
	Neurological system process	0,570
	Circulatory system process	0,526
	Axon	0,444
	Response to hormones	0,434
	Neurological system processes	0,300
	Embryonic development	0,234
	Reproductive process	0,098
Protein metabolism	Protein metabolism	1,897
	sh3 domain	0,888
	Regulation of translation	0,505
	Regulation of protein metabolism	0,457
	Ank repeat	0,453
	Peptidase activity	0,300

	Post-translational protein modification	0,288
	Protein metabolism	0,280
	Protein modification process	0,238
	Protein activity	0,235
	Protein metabolism	0,182
	Proteolysis	0,066
	Protein localization	0,036
Transcription	Regulation of transcription	0,861
	Positive regulation of transcription and biosynthesis	0,788
	Regulation of transcription	0,561
	Chromosome	0,512
	Regulation of transcription	0,500
	Regulation of transcription and biosynthesis	0,471
	RNA binding	0,452
	Transcription activity and binding	0,362
	Transcription regulation	0,325
	Nucleotide binding	0,273
	DNA binding	0,145
Cell, organelles and extracellular region	Intracellular organelle	1,531
	Plasma membrane	1,294
	Fibronectin	1,261
	Cytoskeleton organization	1,016
	Apical part of cell	0,932
	Cytoskeleton	0,839
	Organelle organization	0,753
	Cellular fractions	0,690
	Extracellular region	0,605
	Mitochondrion	0,593
	Organelle organization	0,580
	Golgi apparatus	0,571
	Membrane	0,556

	Membrane	0,502
	Organelle	0,477
	Membrane	0,466
	Extracellular matrix	0,406
	Membrane	0,398
	Mitochondrion	0,329
	Organelle	0,303
	Organelle	0,291
	Vesicle	0,254
	Organelle	0,021
Cellular processes	Negative regulation of biosynthetic process	1,130
	Negative regulation of metabolic process	0,994
	Biological regulation	0,991
	Positive regulation of cell communication	0,814
	Positive regulation of cell differentiation and development vs. negative regula	0,761
	Carboxilic acid metabolism	0,647
	Cell adhesion	0,632
	Calcium ion binding	0,611
	Coenzyme and cofactor metabolic process	0,572
	Regulation of molecular function	0,492
	Cell cycle	0,478
	Regulation of apoptosis	0,470
	Kinase activity	0,466
	Metal ion binding	0,383
	Cell adhesion	0,371
	Cell development, morphogenesis and differentiation	0,343
	Cell division	0,330
	Cell motility	0,291
	Localization and transport	0,268
	Metal ion binding	0,210
	Cellular homeostasis	0,181
	Oxidoreductase activity	0,169

	Metabolic process	0,166
	Catabolic process	0,052
	Apoptosis	0,045
	Transmembrane transporter activity	0,026
Immune system processes	Immune system process	0,838
	Immunoglobulin	0,658
	Response to virus and biotic stimulus	0,556
	Immune system development and activation	0,395
	Immune response	0,352
	Cellular response to stress and DNA damage stimulus	0,244
	Response to stimulus	0,239
Fatty acid metabolism	Fatty acid metabolic process	0,420

Functional category	Functional Annotation Cluster	Enrichment Score
**Down-regulated**		
Fatty acid metabolism	Fatty acid metabolism	2,041
	Reproductive process	0,598
	Sensory perception	0,220
Tissue development and maintenance	Development	0,078
Transcription	Nucleotide binding	0,284
	Regulation of transcription	0,025
	Transcription	0,001
Cell, organelles and extracellular region	Mitochondrion	1,232
	Membrane	0,820

	Cytoskeleton	0,476
	Organelle	0,464
	Cytoplasm	0,416
	Organelle	0,229
	Membrane	0,020
Cellular processes	Oxidoreductase activity	0,819
	Protein transport and localization	0,726
	Transport and localization	0,721
	Protein transport and localization	0,641
	Catabolic activity	0,624
	Catabolic activity	0,607
	Proteolitic activity	0,410
	Apoptosis	0,246
	Metabolic process	0,204
	Transport and localization	0,181
	Biological regulation	0,030
	Metal ion binding	0,003
