# Supplementary material for: Dietary Inulin Supplementation Modifies Significantly the Liver Transcriptomic Profile of Broiler Chickens
Source: PLoS One. 2014 Jun 10;9(6):e98942. doi: 10.1371/journal.pone.0098942 (PMC4051581; doi:10.1371/journal.pone.0098942)
Supplement: Table S4 — List of differentially expressed genes encoding proteins of known function but not included in the DAVID FAC analysis, and genes encoding proteins of unknown function. (PDF) [file pone.0098942.s004.pdf]

Table S4. List of differentially expressed genes encoding proteins of known function but not included in the DAVID FAC analysis and genes encoding proteins of unknown function.

Probe Set ID	Ratio With vs. Without	*P*	Public ID	Gene Symbol	Gene Name	Biological process[1]
Gga.5846.1.S1_at	3.8	0.07	BX540658	*PRR13*	Proline rich 13	Transcriptional regulator of thrombospondin-1 (THBS1), gene that mediates cell-to-cell and cell-to-matrix interactions.
Gga.17147.1.S1_s_at	1.8	0.04	BU309463	*CCDC127*	Coiled-coil domain containing 127	No information available
Gga.3693.1.A1	1.5	0.07	AI982495	*B-G*	MHC B-G antigen	The B-G antigens are highly polymorphic antigens encoded by genes located within the major histocompatibility complex (MHC) of the chicken, the B system (Goto et al., 1988).
Gga.16473.1.S1_s_at	1.5	0.00	CR385148.1	*MAP7D2*	MAP7 domain containing 2	MAP7 is a microtubule-associated protein involved in microtubule dynamics, which is essential for cell polarization and differentiation.
GgaAffx.21582.1.S1_at	1.5	0.01	CR523033.1	*SPECC1L (CYTSA)*	Sperm antigen with calponin homology and coiled-coil domains 1-like	May play a critical role in actin-cytoskeletal reorganization during facial morphogenesis.
Gga.6778.2.S1_a_at	1.4	0.02	BX264871	*LOC431317*	Similar to Scale keratin (S-ker) (sKer)	No information available
Gga.9481.1.S1_s_at	1.4	0.09	NM_204932.1	*LOC395772*	Otokeratin	No information available
Gga.772.1.S1_at	1.4	0.04	BI394262	*NNF1*	Nnf1 protein	No information available
GgaAffx.4394.1.S1_at	1.4	0.08	ENSGALT00000011376.1	*LOC416622*	Transmembrane protein 180-like	Similar to putative endoplasmic reticulum protein family member, with at least 7 transmembrane domains, of ancient origin (53.1 kD) (1F495)

GgaAffx.25180.2.A1_at	0.6	0.03	ENSGALT00000027689.1	*LOC771527*	Hypothetical protein LOC771527	No information available
Gga.16376.1.S1_x_at	0.6	0.09	CR386477.1	*LOC101750364*	uncharacterized LOC101750364	No information available
GgaAffx.7666.1.S1_at	0.6	0.07	ENSGALT00000019740.1	*C4H4ORF32 (C4orf32)*	Chromosome 4 open reading frame, human C4orf32	No information available
GgaAffx.25266.1.S1_at	0.6	0.08	ENSGALT00000023473.1	*TTC38*	Tetratricopeptide repeat protein 38-like	No information available
Gga.10636.1.S1	0.6	0.04	BX932098.2	*C21orf7 (TAKL)*	Chromosome 21 open reading frame 7	TAK1-like (TAKL) gene shared a homology with human, mouse, and Xenopus TGF-beta activated kinase (TAK1), which plays a critical role in the TGF-beta signaling transduction pathway (Li et al., 2004). TAK1 controls a variety of cell functions including transcription regulation, apoptosis and, in response to IL-1, the cell response to environmental stresses.
Gga.14439.1.S1_at	0.6	0.06	BX933041.2	*Near NCAPG2*	Non-SMC condensin II complex, subunit G2	Plays essential roles in mitotic chromosome assembly and segregation
GgaAffx.8171.3.S1_s_a	0.6	0.04	ENSGALT00000021054.1	*C5orf22*	Chromosome 5 open reading frame 22	No information available
GgaAffx.10545.1.S1_at	0.6	0.06	ENSGALT00000026579.1	*XPAX*	Similar to egg envelope component ZPAX	Zona pellucida protein
GgaAffx.21915.1.S1_at	0.4	0.09	ENSGALT00000010311.1	*IFIT5*	Interferon-induced protein with tetratricopeptide repeats 5	Immune system processes

[1] Gene information from the public database NCBI (National Center for Biotechnology Information, http://www.ncbi.nlm.nih.gov/gene, August 2013) or the indicated reference

References:

Goto R, Miyada CG, Young S, Wallace RB, Abplanalp H, Bloom SE, Briles WE, Miller MM. (1988) Isolation of a cDNA clone from the B-G subregion of the chicken histocompatibility (B) complex. Immunogenetics. 27, 102-9.

Li J, Ji C, Yang Q, Chen J, Gu S, Ying K, Xie Y, Mao Y. (2004) Cloning and characterization of a novel human TGF-beta activated kinase-like gene. Biochem Genet. 42, 129-37.
