# Supplementary figures and images for: Dietary Inulin Supplementation Modifies Significantly the Liver Transcriptomic Profile of Broiler Chickens
Source: PLoS One. 2014 Jun 10;9(6):e98942. doi: 10.1371/journal.pone.0098942 (PMC4051581; doi:10.1371/journal.pone.0098942)

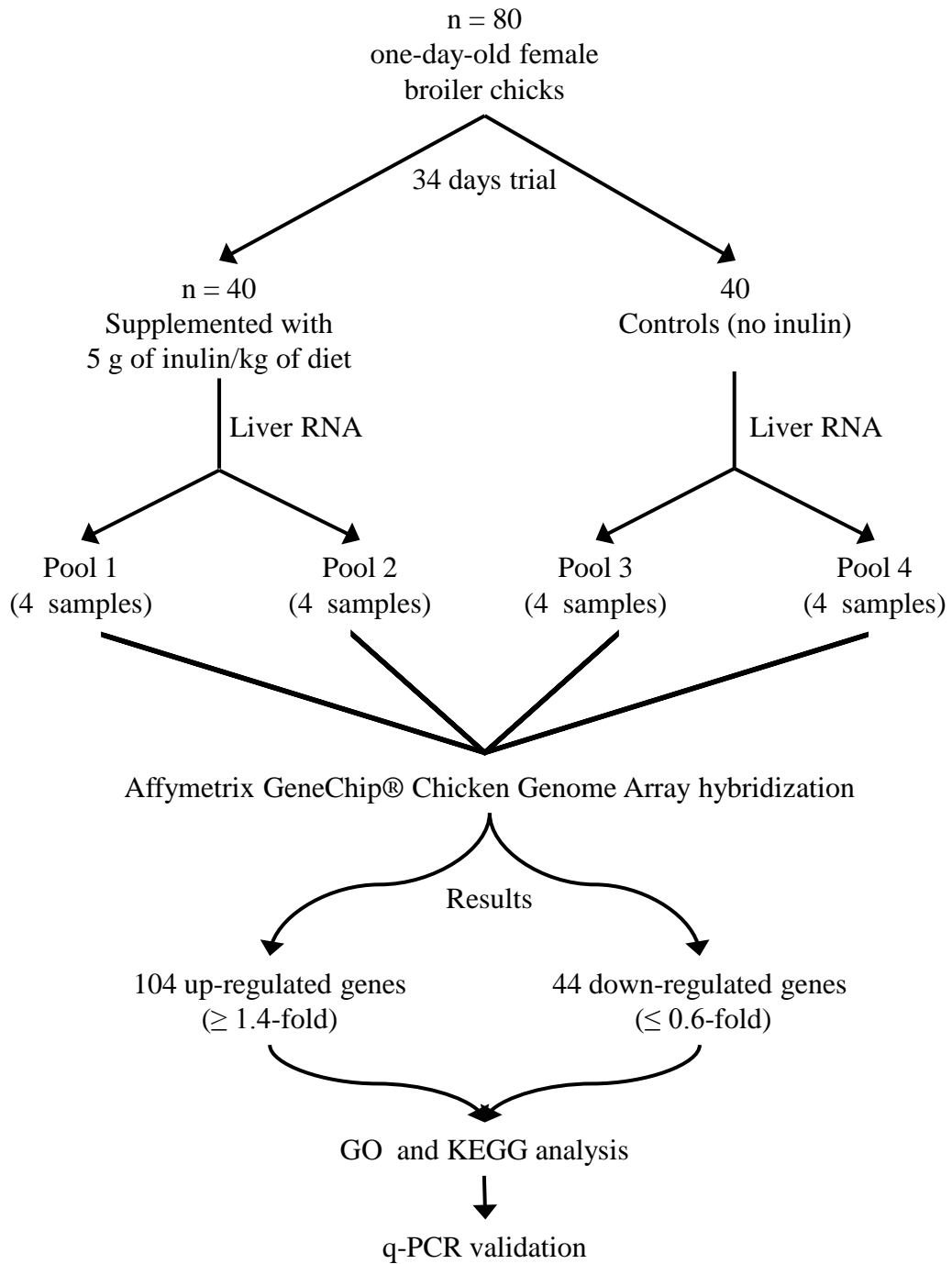

Supplement: Figure S1 — Flow diagram of study design and results. (PDF) [file pone.0098942.s005.pdf]
