# Supplementary material for: Dietary Inulin Supplementation Modifies Significantly the Liver Transcriptomic Profile of Broiler Chickens
Source: PLoS One. 2014 Jun 10;9(6):e98942. doi: 10.1371/journal.pone.0098942 (PMC4051581; doi:10.1371/journal.pone.0098942)
Supplement: Figure S2 — KEGG pathway maps of chicken differentially expressed genes involved in common pathways and processes. KEGG pathway tools were utilized through DAVID online tools and the analysis were conducted on two independent gene lists containing 95 up-regulated genes (≥ 1.4-fold) and 35 down-regulated genes (≤ 0.6-fold) and P ≤ 0.09. (a) Adipocytokine Signaling Pathway; (b) Glycosphingolipid Biosynthesis - Ganglio Series; (c) Glutathione Metabolism; (d) Drug Metabolism - Cytochrome P450; (e) Metabolism of Xenobiotics by Cytochrome P450. Red boxes indicate up-regulated and down-regulated homologs. All chicken homologs identified on the KEGG maps are shown in Table 2. (PDF) [file pone.0098942.s006.pdf]

(a)

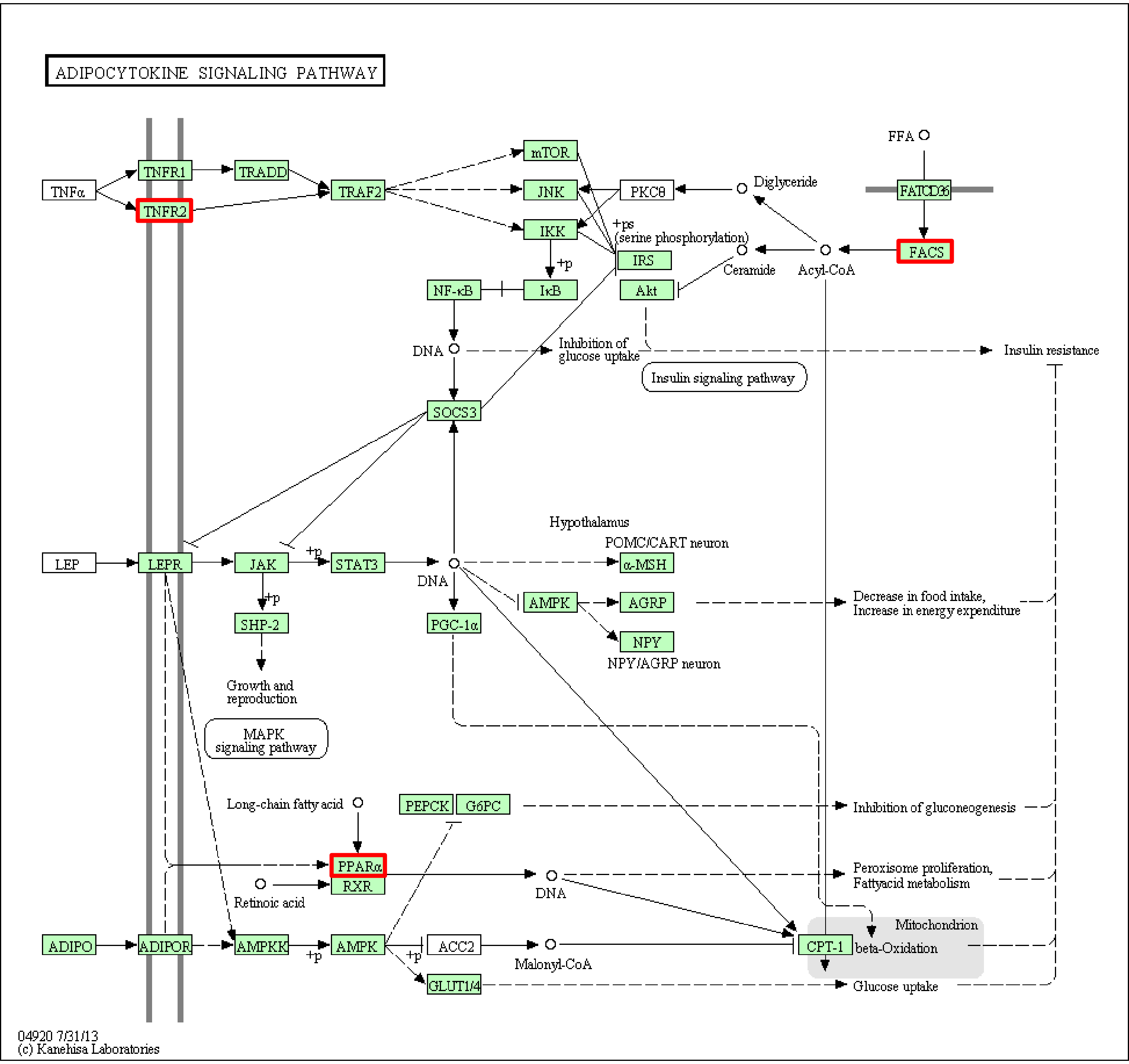
ADIPOCYTOKINE SIGNALING PATHWAY
TNFα
TNFR1
TNFR2
TRADD
TRAF2
mTOR
JNK
IKK
PKCθ
Diglyceride
FFA
FATCD36
FACS
+ps
(serine phosphorylation)
IRS
Akt
Ceramide
Acyl-CoA
+p
NF-κB
IκB
DNA
Inhibition of
glucose uptake
Insulin signaling pathway
Insulin resistance
SOCS3
Hypothalamus
POMC/CART neuron
LEP
LEPR
JAK
+p
STAT3
α-MSH
DNA
AMPK
AGRP
Decrease in food intake,
Increase in energy expenditure
+p
SHP-2
PGC-1α
NPY
NPY/AGRP neuron
Growth and
reproduction
MAPK
signaling pathway
Long-chain fatty acid
PEPCK
G6PC
Inhibition of gluconeogenesis
PPARα
RXR
Retinoic acid
DNA
Peroxisome proliferation,
Fattyacid metabolism
Mitochondrion
ADIPO
ADIPOR
AMPKK
AMPK
ACC2
Malonyl-CoA
CPT-1
beta-Oxidation
+p
+p
GLUT1/4
Glucose uptake
04920 7/31/13
(c) Kanehisa Laboratories

(b)

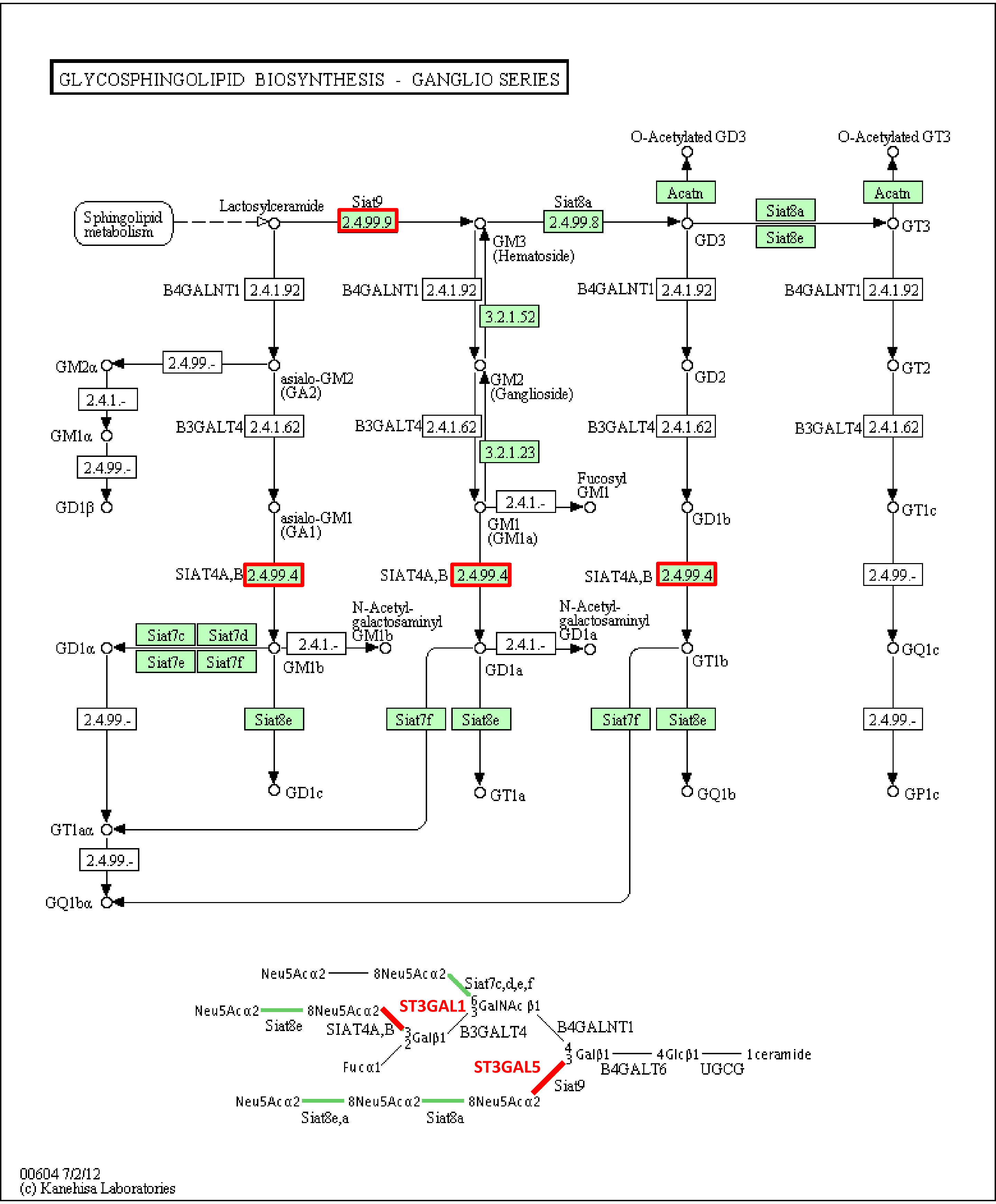

GLYCOSPHINGOLIPID BIOSYNTHESIS - GANGLIO SERIES
Sphingolipid metabolism
Lactosylceramide
Siat9
2.4.99.9
GM3 (Hematoside)
Siat8a
2.4.99.8
GD3
O-Acetylated GD3
Acatn
Siat8a
Siat8e
GT3
O-Acetylated GT3
B4GALNT1 2.4.1.92
3.2.1.52
GM2α
2.4.99.-
asialo-GM2 (GA2)
GM2 (Ganglioside)
GD2
GT2
2.4.1.-
GM1α
B3GALT4 2.4.1.62
3.2.1.23
2.4.99.-
GD1β
asialo-GM1 (GA1)
GM1 (GM1a)
Fucosyl GM1
GD1b
GT1c
SIAT4A,B 2.4.99.4
N-Acetyl-galactosaminyl GM1b
N-Acetyl-galactosaminyl GD1a
GD1α
Siat7c
Siat7d
Siat7e
Siat7f
GM1b
GD1a
GT1b
GQ1c
Siat8e
Siat7f
GD1c
GT1a
GQ1b
GP1c
GT1aα
GQ1bα
Neu5Acα2 — 8Neu5Acα2
Siat7c,d,e,f
Neu5Acα2 — 8Neu5Acα2
Siat8e
ST3GAL1
6 3 GalNAc β1
SIAT4A,B
3 2 Galβ1
B3GALT4
B4GALNT1
Fucα1
4 3 Galβ1 — 4Glcβ1 — 1ceramide
B4GALT6
UGCG
ST3GAL5
Siat9
Neu5Acα2 — 8Neu5Acα2 — 8Neu5Acα2
Siat8e,a
Siat8a
00604 7/2/12
(c) Kanehisa Laboratories

(c)

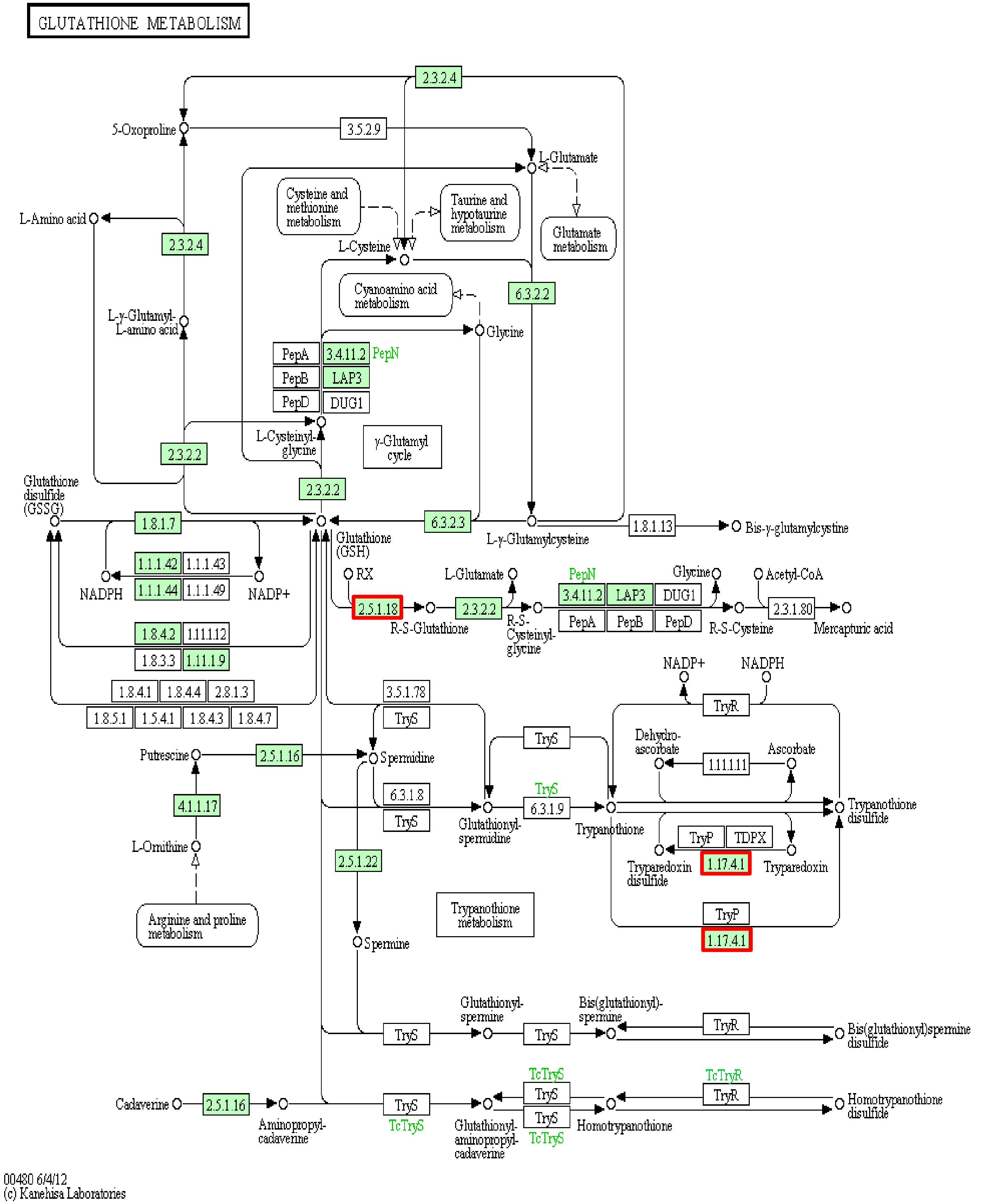
GLUTATHIONE METABOLISM
2.3.2.4
5-Oxoproline
3.5.2.9
L-Glutamate
Cysteine and methionine metabolism
Taurine and hypotaurine metabolism
Glutamate metabolism
L-Amino acid
2.3.2.4
L-Cysteine
Cyanoamino acid metabolism
6.3.2.2
L-γ-Glutamyl-L-amino acid
Glycine
PepA
3.4.11.2
PepN
PepB
LAP3
PepD
DUG1
L-Cysteinyl-glycine
γ-Glutamyl cycle
2.3.2.2
2.3.2.2
Glutathione disulfide (GSSG)
1.8.1.7
6.3.2.3
1.8.1.13
Bis-γ-glutamylcystine
Glutathione (GSH)
L-γ-Glutamylcysteine
1.1.1.42
1.1.1.43
1.1.1.44
1.1.1.49
NADPH
NADP+
RX
L-Glutamate
PepN
Glycine
Acetyl-CoA
2.5.1.18
2.3.2.2
3.4.11.2
LAP3
DUG1
2.3.1.80
PepA
PepB
PepD
R-S-Glutathione
R-S-Cysteinyl-glycine
R-S-Cysteine
Mercapturic acid
1.8.4.2
1.11.1.12
1.8.3.3
1.11.1.9
1.8.4.1
1.8.4.4
2.8.1.3
1.8.5.1
1.5.4.1
1.8.4.3
1.8.4.7
3.5.1.78
TryS
NADP+
NADPH
TryR
TryS
Dehydro-ascorbate
Ascorbate
1.11.1.11
Putrescine
2.5.1.16
Spermidine
6.3.1.8
TryS
6.3.1.9
4.1.1.17
TryS
Glutathionyl-spermidine
Trypanothione
Trypanothione disulfide
TryP
TDPX
L-Ornithine
2.5.1.22
Tryparedoxin disulfide
1.17.4.1
Tryparedoxin
Trypanothione metabolism
TryP
1.17.4.1
Arginine and proline metabolism
Spermine
Glutathionyl-spermine
Bis(glutathionyl)-spermine
TryR
TryS
TryS
Bis(glutathionyl)spermine disulfide
TcTryS
TcTryR
TryS
TryR
Cadaverine
2.5.1.16
TryS
TryS
Homotrypanothione disulfide
Aminopropyl-cadaverine
TcTryS
Glutathionyl-aminopropyl-cadaverine
TcTryS
Homotrypanothione
00480 6/4/12
(c) Kanehisa Laboratories

(d)

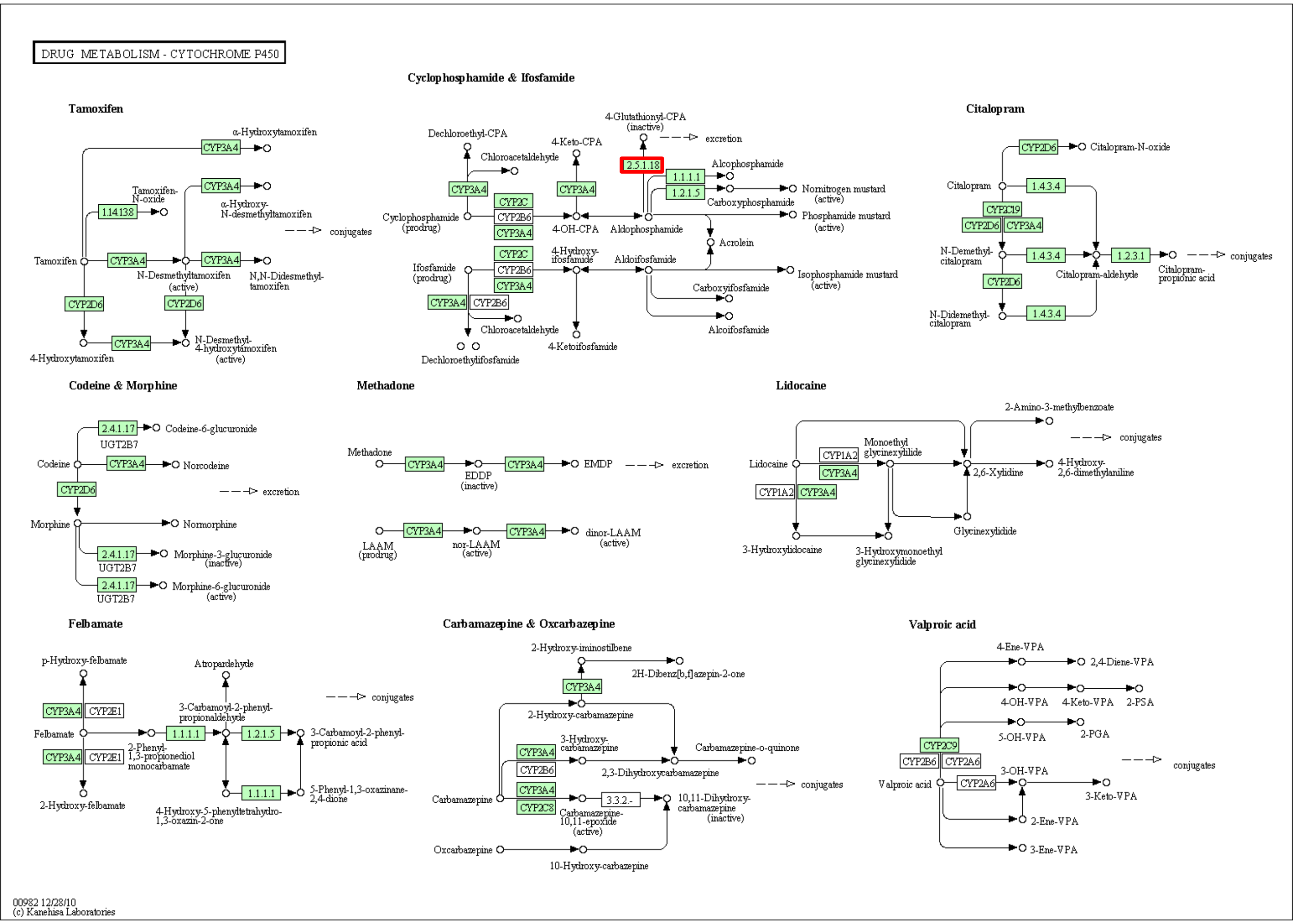

DRUG METABOLISM - CYTOCHROME P450
Cyclophosphamide & Ifosfamide
Tamoxifen
α-Hydroxytamoxifen
CYP3A4
Tamoxifen-N-oxide
1.14.13.8
α-Hydroxy-N-desmethyltamoxifen
conjugates
Tamoxifen
N-Desmethyltamoxifen (active)
N,N-Didesmethyl-tamoxifen
CYP2D6
4-Hydroxytamoxifen
N-Desmethyl-4-hydroxytamoxifen (active)
Dechloroethyl-CPA
Chloroacetaldehyde
4-Keto-CPA
4-Glutathionyl-CPA (inactive)
excretion
2.5.1.18
1.1.1.1
1.2.1.5
Alcophosphamide
Nornitrogen mustard (active)
Carboxyphosphamide
Phosphamide mustard (active)
Cyclophosphamide (prodrug)
CYP2C
CYP2B6
4-OH-CPA
Aldophosphamide
Acrolein
Ifosfamide (prodrug)
4-Hydroxy-ifosfamide
Aldoifosfamide
Isophosphamide mustard (active)
Carboxyifosfamide
Alcoifosfamide
4-Ketoifosfamide
Dechloroethylifosfamide
Citalopram
CYP2D6
Citalopram-N-oxide
1.4.3.4
CYP2C19
N-Demethyl-citalopram
1.2.3.1
Citalopram-aldehyde
Citalopram-propionic acid
N-Didemethyl-citalopram
Codeine & Morphine
2.4.1.17
Codeine-6-glucuronide
UGT2B7
Codeine
Norcodeine
excretion
Morphine
Normorphine
Morphine-3-glucuronide (inactive)
Morphine-6-glucuronide (active)
Methadone
EDDP (inactive)
EMDP
LAAM (prodrug)
nor-LAAM (active)
dinor-LAAM (active)
Lidocaine
2-Amino-3-methylbenzoate
Monoethyl glycinexylidide
CYP1A2
2,6-Xylidine
4-Hydroxy-2,6-dimethylaniline
Glycinexylidide
3-Hydroxylidocaine
3-Hydroxymonoethyl glycinexylidide
Felbamate
p-Hydroxy-felbamate
Atropaldehyde
3-Carbamoyl-2-phenyl-propionaldehyde
CYP2E1
2-Phenyl-1,3-propionediol monocarbamate
3-Carbamoyl-2-phenyl-propionic acid
5-Phenyl-1,3-oxazinane-2,4-dione
2-Hydroxy-felbamate
4-Hydroxy-5-phenyltetrahydro-1,3-oxazin-2-one
Carbamazepine & Oxcarbazepine
2-Hydroxy-iminostilbene
2H-Dibenz[b,f]azepin-2-one
2-Hydroxy-carbamazepine
3-Hydroxy-carbamazepine
Carbamazepine-o-quinone
2,3-Dihydroxycarbamazepine
CYP2C8
3.3.2.-
Carbamazepine
Carbamazepine-10,11-epoxide (active)
10,11-Dihydroxy-carbamazepine (inactive)
Oxcarbazepine
10-Hydroxy-carbamazepine
Valproic acid
4-Ene-VPA
2,4-Diene-VPA
4-OH-VPA
4-Keto-VPA
2-PSA
5-OH-VPA
2-PGA
CYP2C9
CYP2A6
3-OH-VPA
3-Keto-VPA
2-Ene-VPA
3-Ene-VPA
00982 12/28/10
(c) Kanehisa Laboratories

(e)

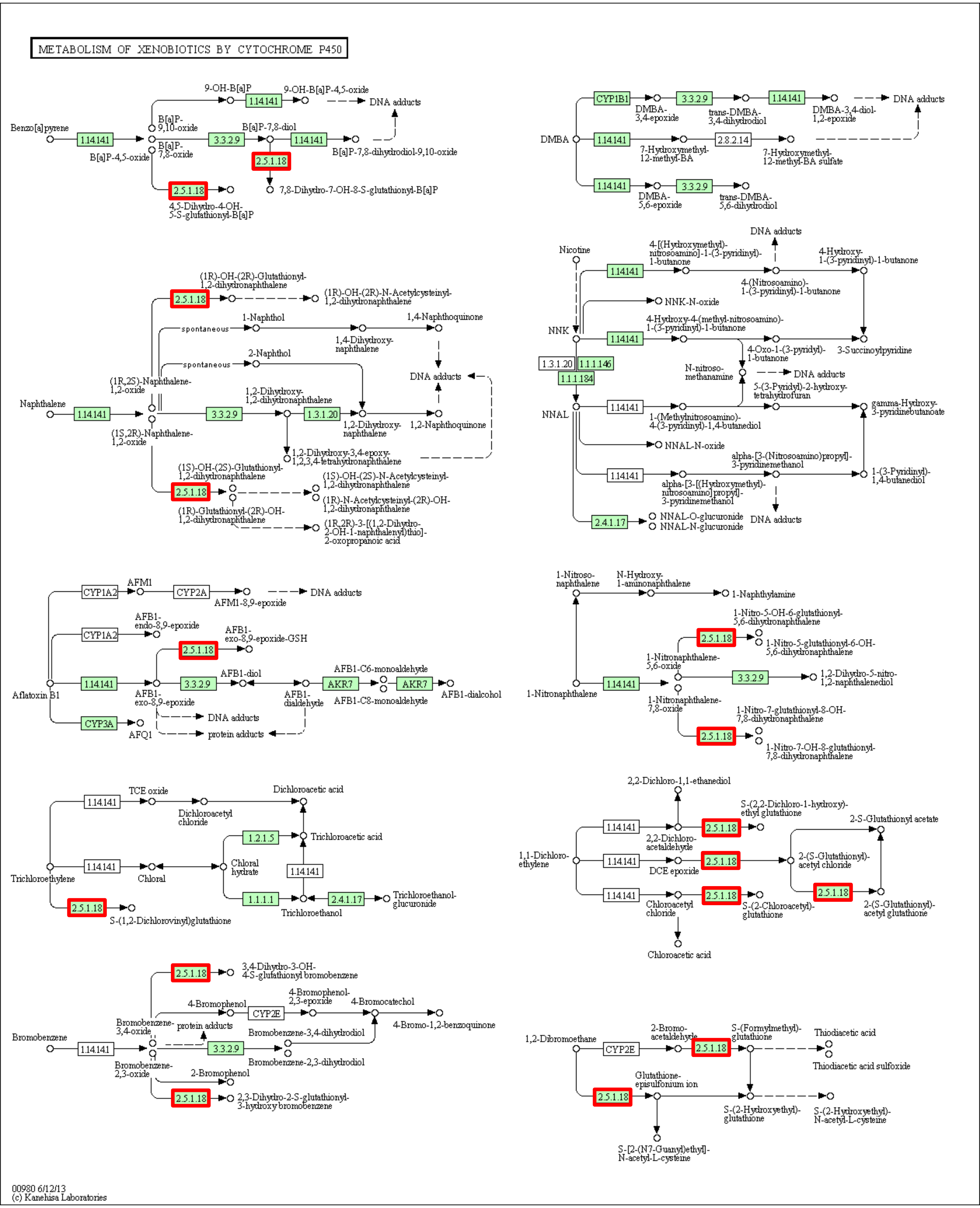

METABOLISM OF XENOBIOTICS BY CYTOCHROME P450
Benzo[a]pyrene
1.14.14.1
B[a]P-9,10-oxide
B[a]P-7,8-oxide
B[a]P-4,5-oxide
9-OH-B[a]P
9-OH-B[a]P-4,5-oxide
DNA adducts
3.3.2.9
B[a]P-7,8-diol
B[a]P-7,8-dihydrodiol-9,10-oxide
2.5.1.18
7,8-Dihydro-7-OH-8-S-glutathionyl-B[a]P
4,5-Dihydro-4-OH-5-S-glutathionyl-B[a]P
DMBA
CYP1B1
DMBA-3,4-epoxide
trans-DMBA-3,4-dihydrodiol
DMBA-3,4-diol-1,2-epoxide
7-Hydroxymethyl-12-methyl-BA
2.8.2.14
7-Hydroxymethyl-12-methyl-BA sulfate
DMBA-5,6-epoxide
trans-DMBA-5,6-dihydrodiol
Naphthalene
(1R,2S)-Naphthalene-1,2-oxide
(1S,2R)-Naphthalene-1,2-oxide
(1R)-OH-(2R)-Glutathionyl-1,2-dihydronaphthalene
(1R)-OH-(2R)-N-Acetylcysteinyl-1,2-dihydronaphthalene
spontaneous
1-Naphthol
1,4-Naphthoquinone
1,4-Dihydroxy-naphthalene
2-Naphthol
1,2-Dihydroxy-1,2-dihydronaphthalene
1.3.1.20
1,2-Dihydroxy-naphthalene
1,2-Naphthoquinone
1,2-Dihydroxy-3,4-epoxy-1,2,3,4-tetrahydronaphthalene
(1S)-OH-(2S)-Glutathionyl-1,2-dihydronaphthalene
(1S)-OH-(2S)-N-Acetylcysteinyl-1,2-dihydronaphthalene
(1R)-Glutathionyl-(2R)-OH-1,2-dihydronaphthalene
(1R)-N-Acetylcysteinyl-(2R)-OH-1,2-dihydronaphthalene
(1R,2R)-3-[(1,2-Dihydro-2-OH-1-naphthalenyl)thio]-2-oxopropanoic acid
Nicotine
NNK
NNAL
1.3.1.20
1.1.1.146
1.1.1.184
4-[(Hydroxymethyl)-nitrosoamino]-1-(3-pyridinyl)-1-butanone
4-(Nitrosoamino)-1-(3-pyridinyl)-1-butanone
4-Hydroxy-1-(3-pyridinyl)-1-butanone
NNK-N-oxide
4-Hydroxy-4-(methyl-nitrosoamino)-1-(3-pyridinyl)-1-butanone
4-Oxo-1-(3-pyridyl)-1-butanone
3-Succinoylpyridine
N-nitroso-methanamine
5-(3-Pyridyl)-2-hydroxy-tetrahydrofuran
1-(Methylnitrosoamino)-4-(3-pyridinyl)-1,4-butanediol
gamma-Hydroxy-3-pyridinebutanoate
NNAL-N-oxide
alpha-[3-(Nitrosoamino)propyl]-3-pyridinemethanol
alpha-[3-[(Hydroxymethyl)-nitrosoamino]propyl]-3-pyridinemethanol
1-(3-Pyridinyl)-1,4-butanediol
2.4.1.17
NNAL-O-glucuronide
NNAL-N-glucuronide
Aflatoxin B1
CYP1A2
AFM1
CYP2A
AFM1-8,9-epoxide
AFB1-endo-8,9-epoxide
AFB1-exo-8,9-epoxide-GSH
AFB1-exo-8,9-epoxide
AFB1-diol
AFB1-dialdehyde
AKR7
AFB1-C6-monoaldehyde
AFB1-C8-monoaldehyde
AFB1-dialcohol
CYP3A
AFQ1
protein adducts
1-Nitroso-naphthalene
N-Hydroxy-1-aminonaphthalene
1-Naphthylamine
1-Nitronaphthalene
1-Nitronaphthalene-5,6-oxide
1-Nitronaphthalene-7,8-oxide
1-Nitro-5-OH-6-glutathionyl-5,6-dihydronaphthalene
1-Nitro-5-glutathionyl-6-OH-5,6-dihydronaphthalene
1,2-Dihydro-5-nitro-1,2-naphthalenediol
1-Nitro-7-glutathionyl-8-OH-7,8-dihydronaphthalene
1-Nitro-7-OH-8-glutathionyl-7,8-dihydronaphthalene
Trichloroethylene
TCE oxide
Dichloroacetyl chloride
Dichloroacetic acid
1.2.1.5
Trichloroacetic acid
Chloral
Chloral hydrate
1.1.1.1
Trichloroethanol
Trichloroethanol-glucuronide
S-(1,2-Dichlorovinyl)glutathione
1,1-Dichloro-ethylene
2,2-Dichloro-1,1-ethanediol
2,2-Dichloro-acetaldehyde
S-(2,2-Dichloro-1-hydroxy)-ethyl glutathione
DCE epoxide
2-(S-Glutathionyl)-acetyl chloride
2-S-Glutathionyl acetate
Chloroacetyl chloride
S-(2-Chloroacetyl)-glutathione
2-(S-Glutathionyl)-acetyl glutathione
Chloroacetic acid
Bromobenzene
Bromobenzene-3,4-oxide
Bromobenzene-2,3-oxide
3,4-Dihydro-3-OH-4-S-glutathionyl bromobenzene
4-Bromophenol
CYP2E
4-Bromophenol-2,3-epoxide
4-Bromocatechol
4-Bromo-1,2-benzoquinone
protein adducts
Bromobenzene-3,4-dihydrodiol
Bromobenzene-2,3-dihydrodiol
2-Bromophenol
2,3-Dihydro-2-S-glutathionyl-3-hydroxy bromobenzene
1,2-Dibromoethane
2-Bromo-acetaldehyde
S-(Formylmethyl)-glutathione
Thiodiacetic acid
Thiodiacetic acid sulfoxide
Glutathione-episulfonium ion
S-(2-Hydroxyethyl)-glutathione
S-(2-Hydroxyethyl)-N-acetyl-L-cysteine
S-[2-(N7-Guanyl)ethyl]-N-acetyl-L-cysteine
00980 6/12/13
(c) Kanehisa Laboratories
